# G9a Inhibition Promotes Neuroprotection through GMFB Regulation in Alzheimer’s Disease

**DOI:** 10.14336/AD.2023.0424-2

**Published:** 2024-02-01

**Authors:** Aina Bellver-Sanchis, Qizhi Geng, Gemma Navarro, Pedro A. Ávila-López, Júlia Companys-Alemany, Laura Marsal-García, Raquel Larramona-Arcas, Lluisa Miró, Anna Perez-Bosque, Daniel Ortuño-Sahagún, Deb Ranjan Banerjee, Bhanwar Singh Choudhary, Francesc X Soriano, Coralie Poulard, Mercè Pallàs, Hai-Ning Du, Christian Griñán-Ferré

**Affiliations:** ^1^Department of Pharmacology and Therapeutic Chemistry, Institut de Neurociències-Universitat de Barcelona, 08028 Barcelona, Spain.; ^2^Hubei Key Laboratory of Cell Homeostasis, Frontier Science Center for Immunology and Metabolism, RNA Institute, College of Life Sciences, Wuhan University, Wuhan 430072, China.; ^3^Centro de Investigación en Red, Enfermedades Neurodegenerativas (CIBERNED), Instituto de Salud Carlos III, Madrid, Spain.; ^4^Department Biochemistry and Physiology, Faculty of Pharmacy. Universitat de Barcelona, 08028 Barcelona, Spain.; ^5^Department of Biochemistry and Molecular Genetics, Feinberg School of Medicine, Northwestern University, Chicago, Illinois, USA.; ^6^Department of Biochemistry, McGill University, Montréal, Québec, Canada.; ^7^Rosalind and Morris Goodman Cancer Institute, McGill University, Montréal, Québec, Canada.; ^8^Department of Cell Biology, Physiology, and Immunology, Celltec-UB, University of Barcelona, Barcelona, Spain, and Institute of Neurosciences, University of Barcelona, 08028 Barcelona, Spain.; ^9^Departament de Bioquímica i Fisiologia, Facultat de Farmàcia i Ciències de l'Alimentació and Institut de Nutrició i Seguretat Alimentària, Universitat de Barcelona, 08028 Barcelona, Spain.; ^10^Laboratorio de Neuroinmunología Molecular, Instituto de Investigación de Ciencias Biomédicas (IICB) CUCS, Universidad de Guadalajara, Jalisco 44340, México.; ^11^Department of Chemistry, National Institute of Technology Durgapur, India.; ^12^Department of Pharmacy, Central University of Rajasthan, Ajmer, Rajasthan, India.; ^13^Shree S. K. Patel College of Pharmaceutical Education and Research, Ganpat University, Mehsana, Gujarat, India.; ^14^Cancer Research Cancer Lyon, Université de Lyon, F-69000 Lyon, France.; ^15^Inserm U1052, Centre de Recherche en Cancérologie de Lyon, F-69000 Lyon, France.; ^16^CNRS UMR5286, Centre de Recherche en Cancérlogie de Lyon, F-69000 Lyon, France.

**Keywords:** cognitive decline, aging, epigenetics, G9a inhibition, GMFB, SAMP8

## Abstract

Epigenetic alterations are a fundamental pathological hallmark of Alzheimer’s disease (AD). Herein, we show the upregulation of G9a and H3K9me2 in the brains of AD patients. Interestingly, treatment with a G9a inhibitor (G9ai) in SAMP8 mice reversed the high levels of H3K9me2 and rescued cognitive decline. A transcriptional profile analysis after G9ai treatment revealed increased gene expression of glia maturation factor β (GMFB) in SAMP8 mice. Besides, a H3K9me2 ChIP-seq analysis after G9a inhibition treatment showed the enrichment of gene promoters associated with neural functions. We observed the induction of neuronal plasticity and a reduction of neuroinflammation after G9ai treatment, and more strikingly, these neuroprotective effects were reverted by the pharmacological inhibition of GMFB in mice and cell cultures; this was also validated by the RNAi approach generating the knockdown of *GMFB*/*Y507A.10* in *Caenorhabditis elegans*. Importantly, we present evidence that GMFB activity is controlled by G9a-mediated lysine methylation as well as we identified that G9a directly bound GMFB and catalyzed the methylation at lysine (K) 20 and K25 *in vitro*. Furthermore, we found that the neurodegenerative role of G9a as a GMFB suppressor would mainly rely on methylation of the K25 position of GMFB, and thus G9a pharmacological inhibition removes this methylation promoting neuroprotective effects. Then, our findings confirm an undescribed mechanism by which G9a inhibition acts at two levels, increasing GMFB and regulating its function to promote neuroprotective effects in age-related cognitive decline.

## INTRODUCTION

Over the last decade, several *in vitro* and *in vivo* studies have demonstrated that G9a inhibition reduces neurodegeneration and cognitive impairment [3, 12]. Indeed, inhibition of G9a by BIX01294 prevented the amyloid-β (Aβ) oligomer, and induced late-long term potentiation (LTP), and synaptic plasticity by increasing the gene expression of *brain-derived neurotrophic factor* (*Bdnf)*, which was previously repressed [3, 7, 12, 19]. Subsequently, three selective G9a inhibitors (G9ai) (A-366, UNC0638, and UNC0642) demonstrated neuroprotective effects [20]. Recently, our group demonstrated that pharmacological inhibition of G9a by UNC0642 produced significant neuroprotective effects in an early-onset AD mouse model [12]. However, the precise mechanism by which G9a inhibition promotes neuroprotection remains elusive.

Several pieces of evidence thus indicate the wide involvement of H3K9me2 in the central nervous system (CNS) function and disease. In the present study, we confirmed the high levels of H3K9me2 in samples of AD human patients. Importantly, we focused on undescribed modulated pathways involving G9a inhibition that underlie how H3K9me2 promotes changes in the transcriptome of the CNS. Then, RNA-seq data analysis was performed to further explore differential gene expression under G9a inhibition. Finally, inspection of H3K9me2 chromatin immunoprecipitation assays with sequencing (ChIP-seq) revealed that H3K9me2 is enriched in genes associated with neural functions in cells treated with a G9ai.

Our analysis showed that pharmacological G9a inhibition induces changes in different molecular pathways. Unexpectedly, Glia maturation factor β (GMFB) increased after G9a inhibition in senescence-accelerated mice prone 8 (SAMP8), suggesting its transcriptional regulation by G9a. GMFB is expressed predominantly in the CNS [21]. It is a highly evolutionarily conserved protein and is expressed in a wide range of animals [22], including invertebrates [21]. Interestingly, GMFB participates in several inflammatory and immune conditions [23]. As mentioned above, PTMs play a crucial role in almost all biological processes [23], and one example is the SUMOylation, which has been considered to play a role in inflammatory processes [24]. A recent study reported that GMFΒ can be mono-SUMOylated at multiple site by covalent addition of a single SUMO1 protein, fostering the stability and trans-localization of GMFB [23]. Another important clue is that GMFB is implicated in the regulation of neuronal and glial growth and differentiation [25-27]. Furthermore, GMFB expression is altered in several neurodegenerative diseases, suggesting that GMFB may be a suitable disease biomarker [28].

Of note, G9a and GMFB are associated with neuroinflammation, increasing pro-inflammatory cytokines. Likewise, they are known to play a key role in several neurodegenerative diseases [29, 30], and participate in neuronal plasticity, among other degenerative pathways [23, 31, 32]. However, neither the transcriptional regulation of GMFB by G9a nor the G9a-mediated methylation of GMFB have been described. Thus, here we present a previously undescribed mechanism by which G9a inhibition increases GMFB and mediates changes in its function, at the same time directly bound, which in turn mediates the neuroprotective effects.

## MATERIAL AND METHODS

### Animals and treatment

For the in vivo study, 24-week-old female and male SAMP8 and SAMR1[33, 34] mice (n = 79) were used to perform cognitive and molecular studies. We divided these mice randomly into five groups: SAMR1 (n = 24), SAMP8 control (n = 24), SAMP8 G9ai (UNC0642, 5 mg/Kg); n = 17), SAMP8 treated with GMFBi ((1H-Indazol-4-yl)methanol[35], 12mg/Kg), and SAMP8 treated with G9ai (UNC0642, 5 mg/Kg) + GMFBi ((1H-Indazol-4-yl)methanol, 12mg/Kg) (n = 7). The sample size for the intervention was chosen following previous studies in our laboratory and using one of the available interactive tools (http://www.biomath.info/power/index.html). Experimental groups either received a daily dose of vehicle (2% w/v, (2-hydroxypropyl)-β-cyclodextrin) or a dose of 5 mg/Kg/day of UNC0642 and/or a dose of 12 mg/Kg/day (1H-Indazol-4-yl)methanol dissolved in 2% 2-hydroxypropyl-β-cyclodextrin via oral gavage for 4 weeks. Animals had free access to food and water and were kept under standard temperature conditions (22 ± 2°C) and 12h:12h light-dark cycles (300 lux/0 lux). After the treatment period, cognitive tests were performed on the animals.

*C. elegans* were cultured according to standard procedures [36], unless otherwise noted. N2 (WT) (Bristol) strain, and the transgenic AD strains (CL2006, CL2355, and its control, CL2122) were used for this study. They were obtained from the Caenorhabditis Genetics Center (CGC), University of Minnesota, Minneapolis, MN, USA. Moreover, KDs of set-25 and Y50D7A.10 strains were generated in this work using the RNAi method in CL2122, CL2355 and CL2006 strains. WT nematodes were propagated at 20°C, while transgenic AD strains were maintained at 16°C in a temperature-controlled incubator on solid nematode growth medium (NGM) seeded with Escherichia coli (E. coli) OP50. To obtain synchronized animals, young adults’ laid embryos for 24 hours before being removed from the plates.

### RNAi

RNAi was performed using the feeding method as previously described [37]. Bacteria HT115(DE3) carrying IPTG-inducible were incubated at 37°C for 7-8 hours with 100 mg/mL ampicillin. NGM plates were then seeded with 25 mg/mL carbenicillin and 1 mM IPTG with the incubated cultures and left to grow overnight at room temperature. Young adults were plated onto RNAi bacteria at 16ºC. Silenced adults were transferred to fresh OP50 plates to produce subsequent generations 7 days later.

### Behavioral and cognitive tests

*Chemotaxis assay:* Fifteen adult hermaphrodites were left to lay eggs for 24h and then removed from the plates. Eggs were incubated at 16°C for 36h, and then at 23°C for another 48h. Briefly, the assay was performed in 100 mm NGM plates, and 10 µL of control odorant (96% ethanol) was added to the “control” spot. On the opposite side of the plate, 10 µL of odorant (0.025% benzaldehyde in 96% ethanol) was added to the “attractant” spot. Adult worms were washed three times in M9 buffer, and at least 120 worms were placed towards the center of the plate. The test plates were incubated at 23°C for 1.5h, and worms were scored according to the chemotaxis index (CI) as follows: CI = (number of worms at attractant-number of worms at control)/total number of worms.

*Three-Chamber Test (TCT)*:The social behavior of the mice was evaluated in the TCT following a previously described protocol [38]. A box of transparent polyvinyl chloride (15x15x20 cm) divided into three equally dimensioned rooms between openings among them was used. First, each mouse was placed in the center of the box and allowed to explore the three chambers for 5 min (habituation). Afterwards, an intruder (same-sex and age) was placed in a metal cage in one of the rooms, and the behavior was recorded for 10 min. The time spent in each room and interacting with the intruder (sniffing time) was measured manually. The TCT apparatus was cleaned with 70% ethanol between the trials to eliminate olfactory cues.

*Novel object recognition test (NORT):* Short- and long-term recognition memory involving cortical areas and the hippocampus was evaluated by NORT. The experimental apparatus used for this test was a 90-degree, two-arm, 25-cm-long, 20-cm-high maze of black polyvinyl chloride. Light intensity in the middle of the field was 30 lux. First, mice were individually habituated to the apparatus for 10 min per day for 3 days. On day 4, the animals were allowed to freely explore two identical objects (A and A or B and B) placed at the end of each arm for a 10 min acquisition trial (first trial-familiarization). Then, a 10-min retention trial (second trial) was carried out 2 h (short-term memory) or 24 h (long-term memory) later. During the Short-term memory retention test, the times that the animal spent exploring the new object (TN) and the old object (TO) was recorded. Twenty-four hours after the acquisition trial, the mice were tested again, with a new object and an object identical to the new one in the previous trial (A and C, or B and C). TN and TO were measured from the video recordings from each trial session. A Discrimination index (DI) was defined as (TN-TO)/(TN+TO). The maze, the surface, and the objects were cleaned with 70% ethanol between the animals’ trials to eliminate olfactory cues.

*Object Location Test (OLT)*: The OLT evaluated the spontaneous tendency of rodents to spend more time exploring a novel object location than a familiar object location and recognizing when an object has been relocated. OLT was performed using a white plywood apparatus (50 × 50 × 25 cm), in which three walls were white and one was black. On the first day, animals were just habituated to the empty open field arena for 10 minutes. On the second day, two objects were placed in front of the black wall, equidistant from each other and the wall. The objects were 10 cm high and identical. The animals were placed into the open field arena and allowed to explore both objects and surroundings for 10 minutes. Afterward, animals were returned to their home cages, and the OLT apparatus was cleaned with 70% ethanol. On the third day, one object was moved in front of the opposite white wall to test spatial memory. Trials were recorded using a camera mounted above the open field area, and the total exploration time was determined by scoring TN and TO. DI was calculated, defined as (TN-TO)/(TN+TO).

### Biochemical experiments

*Brain processing*: SAMP8 and SAMR1 mice were euthanized 3 days after completion of the behavioral test by cervical dislocation. Brains were immediately removed from the skull. Cortex and hippocampus were then isolated and frozen on powdered dry ice. They were maintained at -80 °C for biochemical experiments. For the Golgi staining protocol, see the procedure in the section “Spine density and Golgi staining protocol”.

*Human cases*: Tissue samples were obtained from the Institute of Neuropathology-IDIBELL Brain Bank, Hospitalet de Llobregat, following the guidelines of Spanish legislation on this matter and the approval of the local ethics committee (Supplementary Table 1). The postmortem interval between death and tissue processing was 1 to 10 h and samples were processed to minimize postmortem delay artifacts. The brain tissue was immediately frozen on metal plates over dry ice, placed in individual air-tight plastic bags, and maintained at -80 °C for biochemical experiments. The average age in the ND group is 69.16 ± 15.06, and in the AD group is 81.13 ± 7.03. The neuropathologic diagnosis of AD was based on the classification of Braak[39] (Supplementary Table 1).

*Reagents*: All the reagents and kits used for the protocols described below are listed in Supplementary Table 2.

### Protein levels determination by Western blotting (WB)

Protein, histone extraction, and immunoblot analysis were performed as in Vasilopoulou et al. [38] (n = 6 hippocampal samples from mice, or at least 6 samples from human brain per experimental group). Proteins were separated by SDS-PAGE (8-20%) and transferred onto PVDF membranes. Afterwards, membranes were blocked in 5% BSA in 0.1% Tween20-TBS (TBS-T) for 1 h at room temperature (RT), followed by overnight incubation at 4 ºC with the primary antibodies presented in Supplementary Table 3.

Immunoreactive proteins were viewed with a chemiluminescence-based detection kit, following the manufacturer’s protocol and digital images were acquired using a ChemiDoc XRS+System. Semi-quantitative analyses were carried out using ImageLab Software and the results were expressed in Arbitrary Units (AU), considering the control mice group as 100%. Protein loading was routinely monitored by immunodetection of GAPDH.

### Human Aβ levels quantification by ELISA

Amyloid-β40 and amyloid-β42 protein levels were measured with the human amyloid-β40 ELISA Kit (Invitrogen, #KHB3481) and human amyloid-β42 Ultrasensitive ELISA Kit (Invitrogen, #KHB3544), respectively. The samples were diluted using standard dilution buffer at a percentage of 50% and all procedures followed the manufacturer’s instructions. At least 6 human brain samples per experimental group were used for the quantification.

### Mouse Aβ levels quantification by ELISA

Mice brains (n= 6 per experimental group) were homogenized in cold 5 M guanidine-HCl/50 mM Tris buffer containing a protease inhibitor cocktail. The quantification of amyloid-β40 (Invitrogen, #KMB3481) and amyloid-β42 (Invitrogen, #KMB3441) was performed using the mouse Aβ40 and Aβ42 ELISA Kits, following the manufacturer’s instructions.

### RNA extraction and gene expression determination

Total RNA isolation from cortical samples (n = 6 per experimental group) was carried out using TRIsureTM reagent following the manufacturer’s instructions (Bioline Reagents, #BIO-38032). The yield, purity, and quality of the RNA were determined spectro-photometrically with a NanoDropTM ND-1000 apparatus and an Agilent 2100B Bioanalyzer. RNAs with 260/280 ratios and RIN higher than 7.5, respectively, were selected.

Reverse transcription-polymerase chain reaction (RT-PCR) was performed using the high-capacity cDNA Reverse Transcription kit. SYBR® Green real-time PCR was performed to quantify the mRNA expression of a set of genes listed in Supplementary Table 4 on a Step One Plus Detection System (Applied-Biosystems, Foster City, CA, USA).

To evaluate the relative changes in gene expression, data were analyzed utilizing the comparative cycle threshold (Ct) method (ΔΔCt). Housekeeping gene level was used to normalize differences in sample loading and preparation. Normalization of expression levels was performed with *β-actin* for the SYBR® Green-based real-time PCR results. Each sample was analyzed in triplicate, and the results represented the n-fold difference of the transcript levels among different groups.

### RNA-sequencing

Pools of 4 hippocampal tissue samples from the SAMP8 control and SAMP8 UNC0642 mice (5 mg/Kg) were aligned with the reference genome using Bowtie2[40], and the gene expression level was estimated using RSEM [41]. Differentially expressed genes were identified using the edgeR program[42]. Genes showing altered expression with p<0.05 and more than 1.3-fold changes were considered differentially expressed.

KEGG, Gene Ontology, and GSEA [43] were used to perform the enrichment and pathway analysis by using Enrichr database [44]. Raw data were deposited at the Gene Expression Omnibus (accession GSE189250), raw fastq files for RNA-seq on mouse hippocampus SAMP8 (accession GSE189249).

### ChIP-seq analysis

Publicly available H3K9me2 ChIP-seq data corresponding to AML12 cells (murine hepatocyte cell line) treated with the selective G9a inhibitor, UNC0638[45] were obtained from the Genome Sequence Archive (GSA: CRA002762)[46]. ChIP-seq analysis was performed as previously described[47]. Briefly, quality control was analyzed in FastQC v0.11.8. The reads were mapped to the reference genome (Mus musculus mm10) with Bowtie2 v2.4.2 using default parameters [40]. The unmapped and duplicate reads were filtered with SAMtools v1.11 [48]. The H3K9me2 peaks were determined with MACS2 v2.1.1.2[49]. The ChIP-seq signal was visualized with deepTools2 v3.3.2[50] and the bigwig files were viewed in the IGV genome browser v2.8.12 [51].

To determine the annotated genomic region of H3K9me2 peaks and distribution of transcription factors, we used the ChIPseeker package [52]. To determine TF binding at promoters of the genes regulated by H3K9me2 in AML12 cells treated with UNC0638, we used the ENCODE and ChEA Consensus TFs from ChIP-X tool [53]. The detection of transcription factor binding motifs at H3K9me2 peaks was performed with the MEME-ChIP database [54]. Motifs with an E-value<0.05 were considered statistically significant. The protein-protein interaction network between G9a (EHMT2) and transcription factors was determined using the STRING database.

### Dendritic length, spine density and Golgi staining protocol

Mice were sacrificed by cervical dislocation and brains were removed from the skull (n=6 whole brain per experimental group). Then, the Golgi staining protocol was developed using the FD Rapid GolgiStain kit according to the manufacturer’s instructions (FD NeuroTechnologies, incs, #PK401). For dendritic branching analysis, images of neurons were collected at 20x magnification in an Olympus BX61 microscope coupled to an Olympus DP70 camera. Measurement of neurite length and complexity was performed using NeuronJ macros and Advanced Sholl Analysis. The number of intersections (branch points) within concentric circles of 10 µm radius was measured and compared between groups. Images for analyzing the spine density were acquired using brightfield microscopy with a 50x oil-objective. All neurites analyzed were around 18 µm and they were at a maximum distance of 150 µm from the soma.

### Thioflavin-S staining Aβ aggregation

Adult worms were fixed in 4 % Paraformaldehyde/ Phosphate-buffered saline (PBS) (pH 7.5), for 24 hours at 4°C. On the following day, worms were permeabilized in 5 % fresh β-mercaptoethanol, 1 % Triton X-100, 125 mm Tris (pH 7.5), at 37°C for another 24 hours. Then, nematodes were stained with 0.125% Thioflavin-S (ThS) (Sigma, CAS# 1326-12-1) in 50 % ethanol (EtOH) for 2 min, destained in 50 % EtOH for 2 min, washed 3 times with PBS. Approximately 10 L of Fluoromount G was used to prepare the glass slide for microscopy (Electron Microscopy Sciences, CAT#17984-25). Fluorescence images were acquired using a 20Å~ objective of a fluorescence microscope, and Aß in the head region of worms were quantified blindly by counting the number of positive Th-S spots using ImageJ.

### Primary cell culture

Primary mixed culture of neurons and microglia was prepared from the cortex and striatum of fetuses from 18-19-day-old C57/BL6 pregnant mice. In brief, samples were dissected, carefully stripped of their meninges, and digested with 0.25% trypsin for 30 min at 37ºC. Cells were brought to a single cell suspension by repeated pipetting followed by passage through a 100 µm-pore mesh and pelleted (7 min, 200g). Neurons and glial cells were resuspended in medium and seeded at a density of 400.000 cells/ml in 6-well plates. Cultures were maintained at 37ºC in a humidified 5% CO2 atmosphere and neurobasal medium supplemented with 2 mM L-glutamine, 5% (v/v) FBS, 100 U/ml penicillin/ streptomycin, and 2% (v/v) B27 supplement (Gibco) in a 6-well plate for 12 days.

*Cell viability*: Then cell viability assay is based on the principle that living cells maintain intact cell membranes that exclude certain dyes, like trypan blue 0.4%. For quantification of live cells, cortical and striatal neurons were gently detached and mixed with an equal volume of trypan blue (0.4%). Neurons (%) were counted with a TC20™ Automated Cell Counter.

*Immunocytochemistry*: Primary microglial culture cells seeded in coverslips were fixed in 4% paraformaldehyde for 15 min and washed twice with PBS containing 20mM glycine, before permeabilization with PBS-glycine containing 0.2% Triton X-100. Microglial cells were treated for 1 h with PBS containing 1% BSA and labelled with the antibodies listed in Supplementary Table 3. Samples were washed several times and mounted with 30% Mowiol. Samples were observed in a Leica SP2 confocal microscope (Leica Microsystems). Negative control slides stained without primary and/or secondary antibodies were used to identify potential nonspecific, background fluorescence. Scale bar: 30 µm. Fluorescence was quantified using Fiji-Image J software.

### Plasmids

The gene encoding the human GMFB was amplified by PCR using the following primers: 5′-CTACGCTGG CCGGCCAGAATTCATGAGTGAGTCTTTGGTTGTTTGTG -3′ (forward) and 5′- CGACTCACTATAGTTCT AGACTAGTGAAAAAATCCAAGTTTCTCACG-3′ (reverse) from HeLa cDNA. The PCR products were cloned into a pCS2-3xHA vector.

Plasmids used to generate the KD of the GMDB methylation sites are listed in the Supplementary Table 5.

### Cell culture, transfection, and treatment

HEK 293T and HeLa S3 cell lines (from passages 1-10) were cultured in DMEM (Dulbecco’s modified Eagle’s medium) medium, supplemented with 10% fetal bovine serum, 2 mM L-glutamine, penicillin (100 U/mL), and streptomycin (100 µg/mL). The HeLa S3 G9a-KO cell line was maintained in selective medium containing puromycin (0.5 µg/ml). Cells were maintained at 37°C with 5% CO2. Transfections were performed using Lipofectamine 2000 according to the manufacturer’s instruction. Cells were harvested 48 hours after transfection and analyzed by immunoprecipitation and WB.

### Co-immunoprecipitation and WB

Cells were collected and lysed in immunoprecipitation (IP) buffer [25 mM tris-HCl (pH 7.4), 150 mM NaCl, 5% glycerol, 1 mM EDTA, and 1% NP-40] containing a protease inhibitor cocktail (BioTool) for 30 min on ice. The Flag resin or HA resin was added into the lysates and incubated at 4°C for 4 hours or overnight with rotation. After washing three times with IP buffer, the resins were resuspended into SDS sample buffer, boiled, and centrifuged. Then, samples were subjected to SDS-polyacrylamide gel electrophoresis (SDS-PAGE), transferred to polyvinylidene difluoride membrane, and immunoblotted with the indicated antibodies (Supplementary Table. 6).

### Data analysis

The statistical analysis was conducted using GraphPad Prism version 9.2 statistical software. Group size may differ depending on power analysis and expertise of the authors and statistical analysis was conducted only for studies where the size of each group was at least n = 5-6 samples per group for in vivo studies; and n = 3-5 replicates for in vitro studies. First, Grubb’s test was performed to detect outliers, in addition to the Shapiro-Wilk test to verify data normality for all groups. Data were expressed as the mean ± Standard Error of the Mean (SEM). For normally distributed data, means were compared in One-Way or Two-Way ANOVA analysis of variance, (ANOVA), followed by Tukey’s post-hoc analysis. Comparison between groups was also performed by two-tailed Student’s t-test for independent samples. In contrast, the Mann-Whitney or Kruskal-Wallis test followed by Dunn’s post-hoc analysis was used for non-normally distributed data. Statistical significance was considered when p-values were <0.05. The statistical outliers were carried out with Grubss’ test and subsequently removed from the analysis. For behavioral tests, a blinded analysis was performed.

## RESULTS

### G9a overexpression in AD patients’ brains and H3K9me2 in both AD patients’ and C. elegans AD transgenic strains correlates with cognitive impairment.

**Figure 1.**
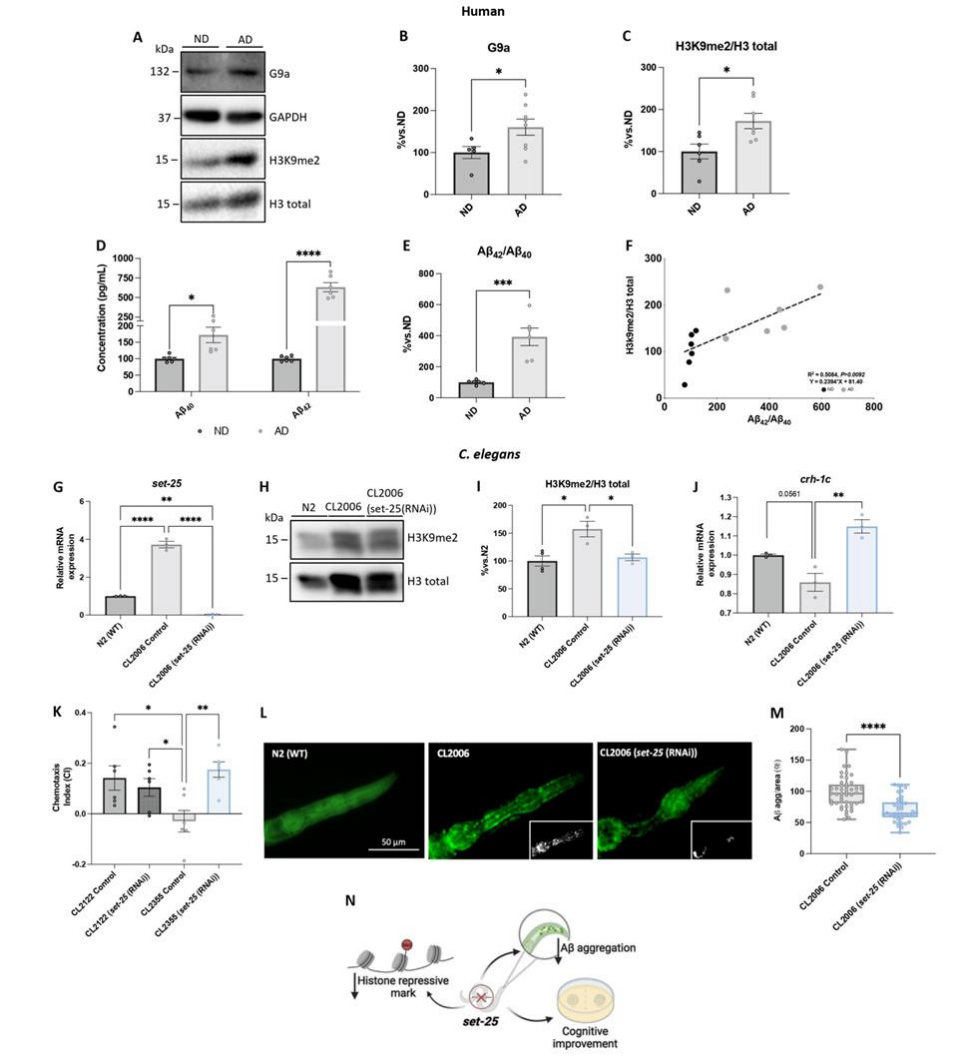
**Overexpression of G9a in AD patients’ brains and H3K9me2 in both AD patients’ and *C. elegans* AD transgenic strains**. (**A**) and (B) Representative WB, and quantification of G9a (EHMT2), and (C) for histone H3K9me2 in human patients’ brains. (**D**) Levels of Aβ_40_ and Aβ_42_. (**E**) The ratio of Aβ_42_/Aβ_40_ by ELISA in human patients’ brains. Values presented are mean ± SEM; (N = 2 groups (ND n = 6, and AD n = 8); Student’s t-test; *p<0.05; ***p<0.001). (**F**) Correlation between Aβ_42_/Aβ_40_ ratio and H3K9me2 (slope = 0.07444). R^2^ and *p*-values are indicated on graphs. (**G**) Representative gene expression of *set-25* in *C. elegans*. (**H**) and (I) Representative WB, and quantification of histone H3K9me2 in N2 (WT), CL2006, and CL2006 (*set-25* (RNAi)). (**J**) Representative gene expression of *chr-1c* in *C. elegans*. Gene expression levels were determined by real-time PCR. Values presented are the mean ± SEM; (N = 3 groups; N2 (WT) n = 3, CL2006 Control n = 3, and CL2006 (*set-*25 (RNAi)) n = 3; Each biological replicate with at least 350 worms in each group from whole petri dish); One-Way ANOVA and post-hoc Tukey’s test; *p<0.05; **p<0.01; ****p<0.0001).(K) Chemotaxis index (CI) calculated for CL2122 (non Aβ strain), CL2355 neuronal Aβ strain), and (CL2355 (*set-25* (RNAi)). Values presented are CI mean ± SEM (N = 4 groups. CL2122 Control n = 5, CL2122 (*set-*25 (RNAi)) n = 6, CL2355 Control n = 6, and CL2355 (*set-*25 (RNAi)) n = 6; Each biological replicate with at least 200 worms for each analysis); (One-Way ANOVAand post-hoc Tukey’s test; *p< 0.05; **p<0.01). (**L**) and (M) Representative images from each group and quantification of Thioflavin S-positive particles in the head region of CL2006 strain and CL2006 (*set-25* (RNAi)) strain. Values presented are mean ± SEM (N = 2 groups; CL2006 Control n = 49; CL2006 (*set-*25 (RNAi)) n = 40; Student’s t-test; ****p<0.0001). (**N**) Representative scheme of the outcomes found in *set-25*-KD. Source data are provided as a Source Data file.

Alterations in the histone methylation marks and their enzymes are implicated in senescence, age-related cognitive decline and neurodegenration [1]. However, the precise mechanism remails elusive. As mentioned above, G9a has been implicated in AD, but to date the precise mechanism by which promotes neurodegeneration is not well described. To characterize G9a role in AD, we thus carried out several experiments and bioinformatic analyses (Supplementary Fig. 1) and examined G9a protein levels by WB in human AD patients’ brains. We found higher levels of this protein in AD patients’ brains compared to the human non-demented (ND) patients’ brains (Figs. 1A and B, Supplementary Table 1). Since G9a is a KMT, we also evaluated H3K9me2, the repressive histone mark. Significantly higher H3K9me2 levels were found in human AD patients than in the ND group (Figs. 1A and C). Furthermore, as expected, the ratio of Aβ_42_/Aβ_40_ was significantly increased in AD patients in comparison with the human ND group (Figs. 1D and E). Of relevance, we also found a strong positive correlation between H3K9me2 levels and Aβ concentration in AD patients (Fig. 1F). Thus, the data reveal that high levels of H3K9me2 are correlated with increased levels of Aβ_42_/Aβ_40_ in human AD patients’ brains, indicating the role of H3K9me2 in the neurodegenerative process of AD.

In *C. elegans* the putative methyltransferase that targets H3K9me2 is SET-25 [55], which is homologous to the mammalian G9a protein (28.8% identity, 44.6% similarity) [56]. Thus, to evaluate the role of SET-25 in aging-related diseases, and more specifically in AD, we created the *set-25* knockdown (KD) using the RNAi approach in *C. elegans*. We used the CL2006 strain, which expresses human Aβ_1-42_ under the control of a muscle-specific promoter, and also presents paralysis with age-worsening [57]. First, we confirmed that the gene expression of *set-25* was higher in the transgenic AD strain, CL2006, than in N2 (WT) (Fig. 1G). As expected, *set-25-*KD in CL2006 (*set-25* (RNAi) were almost completely reduced compared to the CL2006 Control group, and this finding is consistent with the observed reduction in H3K9me2 levels (Figs. 1H and I). Then, we examined the effect of *set-25*-KD on cognition in the CL2355 *C. elegans* strain. This strain expresses Aβ in neuronal cells and has a significantly lower chemotaxis index (CI), a memory parameter [57], than the Control strain, CL2122. It is worth noting that *set-25*-KD fostered the restoration of impaired learning and memory in the CL2355 strain, with a similar CI as in Control worms (Fig. 1K, Supplementary Fig. 2). Accordingly, CL2006 had reduced levels of *crh-1c/*cAMP-response element-binding protein (CREB) [58], which plays an important role in development, lifespan, and learning and memory in *C. elegans* and humans [59, 60] . In our study, increased gene expression of *crh-1c* was observed in the CL2006 (*set-25* (RNAi)) strain (Fig. 1J). Next, we showed a reduction in Aβ aggregation in CL2006 (*set-25* (RNAi)) relative to the CL2006 Control group (Figs. 1L and M), suggesting that *set-25* modulates memory formation by reducing AD pathology. Taken together, these results indicate G9a mediates increased H3K9me2 and cognitive impairment, participating in AD pathophysiology (Fig. 1N).

### Pharmacological G9a inhibition induced a transcriptional profile that allowed beneficial effects on cognitive performance.

**Figure 2.**
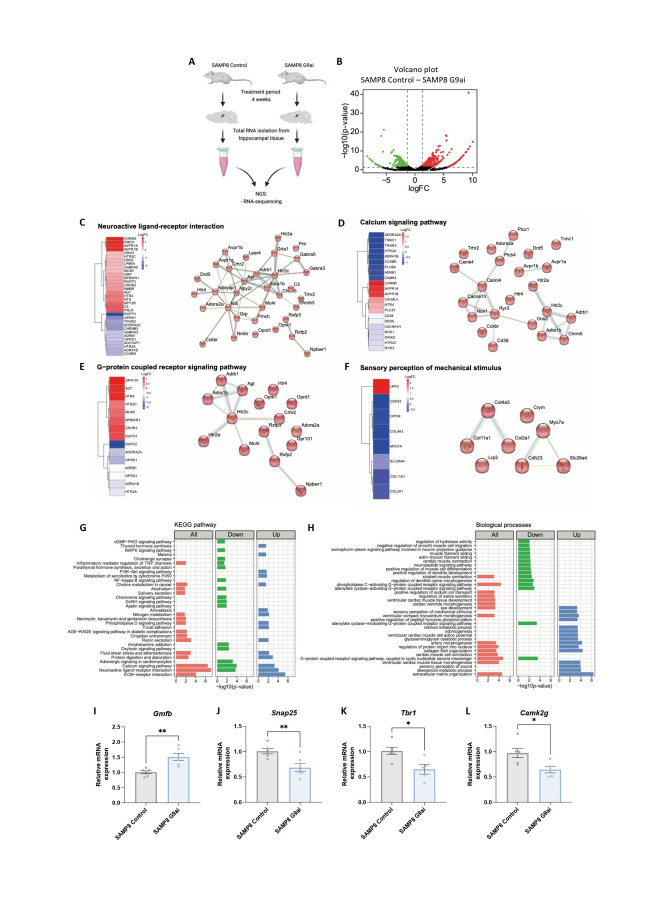
**G9a inhibition treatment induced a transcriptional profile that confirmed its neuroprotective effects in the hippocampus of SAMP8 mice**. (**A**) Treatment, hippocampal extraction, and RNA-sequencing in SAMP8 Control and SAMP8 G9ai. (**B**) Volcano plot showing the DEGs (fold change cutoff of ≥ 1.3, *p*-value < 0.05). Downregulated in green and upregulated in red. (**C**) Heatmaps and interaction network of genes associated with affected processes in G9ai-treated SAMP8 mice, such as the neuroactive ligand-receptor interaction. (**D**) Calcium signaling pathway. (**E**) G-protein coupled receptor signaling pathway. (**F**) Sensory perception of mechanical stimulus. The heatmaps show the logFC. (**G**) KEGG pathways regulated by SAMP8 G9ai. (**H**) Biological processes altered by DEGs in SAMP8 mice treated with G9ai (UNC0642, 5mg/Kg). (**I**) Gene expression levels of *Gmfb*, (J) *Snap25*, (K) *Tbr1*, and (L) *Camk2g*. Gene expression levels were determined by RT-qPCR. Values presented are the mean ± SEM; (N = 2 groups (SAMP8 Control n = 6, and SAMP8 G9ai (UNC0642, 5mg/Kg) n = 6); Student’s t-test; *p<0.05; **p<0.01). Source data are provided as a Source Data file.

To characterize the transcriptional profile associated with the pharmacological inhibition of G9a, we analyzed the transcriptome by RNA sequencing (RNA-seq) in the SAMP8 control and SAMP8 G9ai groups (Fig. 2A, Supplementary Table 7). Differential expression analysis identified 697 differentially expressed genes (DEG) (fold change cutoff of ≥ 1.3, *p*-value < 0.05), among which 217 were reduced, and 480 were increased in G9ai-treated SAMP8 mice (Fig. 2B).

Functional analysis showed that these DEGs regulate processes such as neuroactive ligand-receptor interactions, the calcium signaling pathway and the G-protein coupled receptor signaling pathway (Figs. 2C-E and H), all of which are important in regulating the function and plasticity of neural networks in the CNS[61]. Interestingly, we identified an increase in the expression of genes associated with the sensory perception of mechanical stimulus (Figs. 2F and H), which explains the beneficial effects on behavior and cognition induced by G9ai in the SAMP8 mice. On the other hand, we found a reduction in genes associated with the NF-κB signaling pathway (Fig. 2G), hinting that G9a inhibition could reduce neuroinflammation in the late-onset AD mouse model.

A more detailed analysis performed with Gene Set Enrichment Analysis (GSEA) showed enrichment of processes associated with sensory perception in SAMP8 mice treated with G9ai (Supplementary Figs. 3A). These processes include the sensory processing of sound, sensory perception of the light stimulus, and phototransduction of visible light (Supplementary Figs. 3A and B), supporting the idea that G9ai treatment in the SAMP8 model activates genes that promote beneficial effects on behavior and cognition. Interestingly, we found an increase in interleukin 37 (IL-37) signaling in G9ai-treated SAMP8 mice (Supplementary Figs. 3A and B). IL-37 is an anti-inflammatory cytokine that suppresses immune responses and inflammation in different tissues such as the brain [62]. Of note, we observed the enrichment of processes associated with chromatin methylation, such as the regulation of histone modification and H3K9 methylation (Supplementary Fig. 3), confirming the effect of G9ai on histone methylation status. To validate the RNA-seq, we performed RT-qPCR for some DEGs that are known to be associated with G9a and neurodegeneration, such as *synaptosomal-associated protein 25* (*Snap25*)[63], *T-box brain transcription factor 1* (*Tbr1*) [64], and *calcium/Calmodulin dependent protein kinase II gamma* (*Camk2g*) the expression of which was significantly reduced. Furthermore, significantly increased gene expression of *Gmfb* was found (Fig. 2I-L). Interestingly, this transcriptional enhancement of GMFB mediated by G9a inhibition has not been described previously. As mentioned earlier, GMFB is predominantly expressed in the CNS and plays an important role in neuroinflammation, leading to the overproduction of pro-inflammatory cytokines [30]. However, GMFB is a highly conserved brain-enriched protein implicated in neuroplasticity since a peak in these protein levels was correlated with learning and memory formation [65].

Taken together, these data suggest that treatment with G9ai induces a transcriptional profile that has beneficial effects on behavior and cognition.

### H3K9me2 enrichment regulates pathways associated with the neuronal system after G9a inhibition.

**Figure 3.**
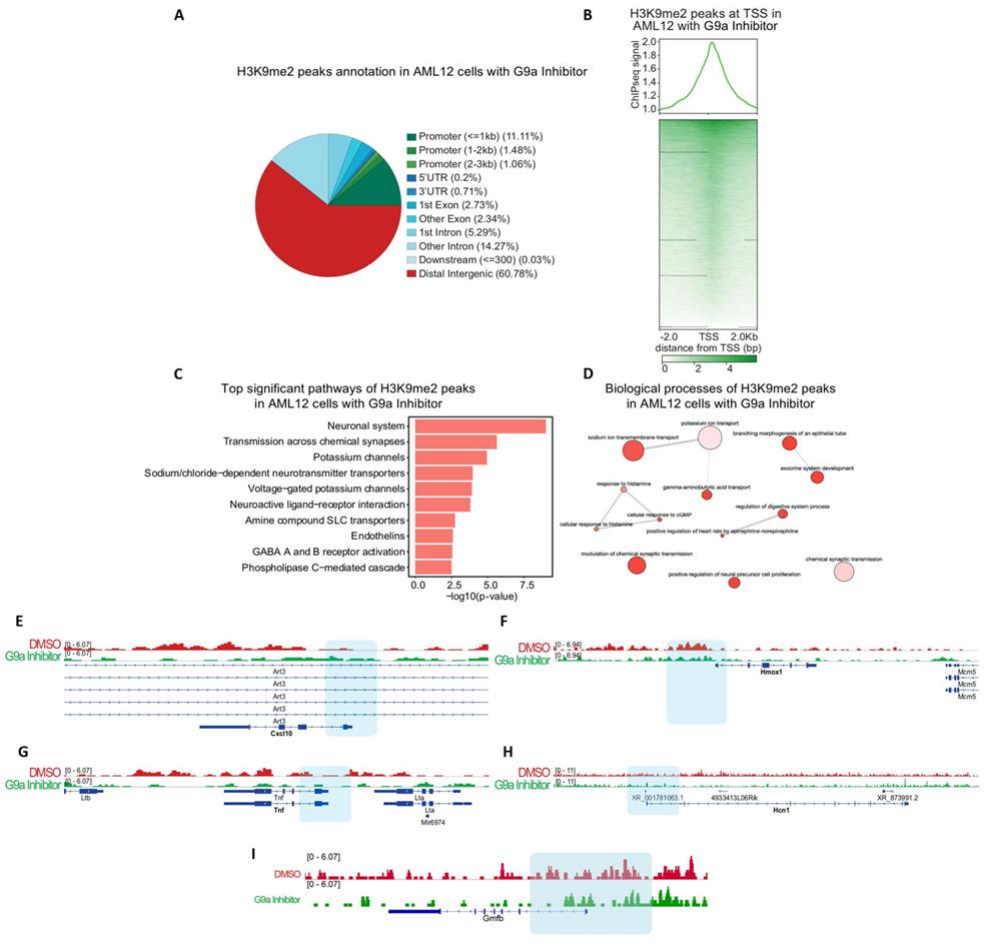
**H3K9me2 enrichment in genes associated with the neuronal system after G9a modulation**. (**A**) Genome region annotation of H3K9me2 peaks in AML12 cells treated with the G9ai (UNC0638). (**B**) Heatmap of H3K9me2 enrichment around the TSS regions of the mouse genome (mm10). (**C**) Pathways regulated by the H3K9me2 enriched genes in AML12 cells treated with G9ai. (**D**) Revigo visualization of biological processes analyzed by Gene Ontology (GO). (**E**)-(I) IGV tracks of H3K9me2 enrichment at the promoters of *Cxcl10, Hmox-1*, *Tnf-α*, *Hcn1*, and *Gmfb* in AML12 cells treated with G9ai and DMSO as a control. The blue area defines the promoters.

We next sought to further characterize the dynamics of the histone mark H3K9me2 after treatment with a G9ai. We used public H3K9me2 ChIP-seq data corresponding to AML12 cells (murine hepatocyte cell line) treated with UNC0638, a selective G9ai[45] with the same specificity as UNC0642. Analysis of H3K9me2 enrichment revealed the presence of this histone mark in distal regions (60.78%) and promoters (13.65%) in cells treated with the G9ai (Fig. 3A). Furthermore, H3K9me2 has an enrichment around TSS (Fig. 3B), suggesting a role in transcriptional regulation.

Interestingly, functional analysis showed that H3K9me2 is enriched at promoters of genes associated with nervous tissue such as the neuronal system, transmission across chemical synapses, the neuroactive ligand-receptor interaction, positive regulation of neural precursor cell proliferation, and gamma-aminobutyric acid transport, among others (Figs. 3C and D). Figs. 3E-G shows the H3K9me2 enrichment in cells treated with G9ai compared to the control group treated with dimethyl sulfoxide (DMSO) in the genes *C-X-C motif chemokine ligand 10* (*Cxcl10*), *tumor necrosis factor-alpha* (*Tnf-α*), *heme oxygenase-1* (*Hmox-1*), *hyperpolarization activated cyclic nucleotide-gated potassium channel 1* (*Hcn1*), and *Gmfb.*

The distribution of the transcription factor motifs relative revealed enrichment of binding motifs around TSS (Supplementary Fig. 5A, and Table 8). To evaluate which transcription factors are associated with H3K9me2, we performed a motif discovery analysis identifying motifs associated with *PR/SET Domain 6* (*Prdm6*), *cytoplasmic polyadenylation element binding protein 1* (*Cpeb1*), and *TATA-Box binding protein associated factor 1* (*Taf1*), among others (Supplementary Fig. 5B). Additionally, an ENCODE and ChEA Consensus TF analysis identified an association with *SUZ12 polycomb repressive complex 2 subunit* (*Suz12*), *enhancer of zeste 2 polycomb repressive complex 2 subunit* (*Ezh2*), *RE1 silencing transcription factor* (*Rest*) and SMAD family member 4 (*Smad4*) (Supplementary Fig. 5C), suggesting a regulatory network associated with transcriptional repression where G9a could be a central regulator (Supplementary Fig. 5D).

Previous results suggest that following G9ai treatment, the H3K9me2 could be reduced, allowing the gene expression. To address this hypothesis in human

brain samples, we performed RT-qPCR for several genes, including *CXCL10, TNF-α*, *HMOX-1*, and *HCN1*, which were significantly altered in AD patients (Figs. 6A). Likewise, we evaluated *Cxcl10, Tnf-α*, and *Hmox-1* in G9ai-treated SAMP8 mice, and no changes were found in *Hcn-1* gene expression in the treated group relative to the Control group (Fig. 6B).

Notably, and consistent with our RNA-seq results, we found a reduction of H3K9me2 at TSS and gene body of GMFB after G9a inhibition (Figs. 2I and 3I). This could indicate that G9a inhibition could reduce the levels of the repressive mark H3K9me2 in the GMFB gene which could promote an increase in its expression. Together, these results demonstrate an H3K9me2 enrichment in genes associated with neural function, suggesting that after treatment with a G9ai, the H3K9me2 reduction could allow the expression of genes associated with the correct function of the CNS would be enhanced.

### Pharmacological G9a inhibition leads to a reduction in H3K9me2 and AD hallmarks, restoring dendritic spine density in SAMP8 mice.

**Figure 4.**
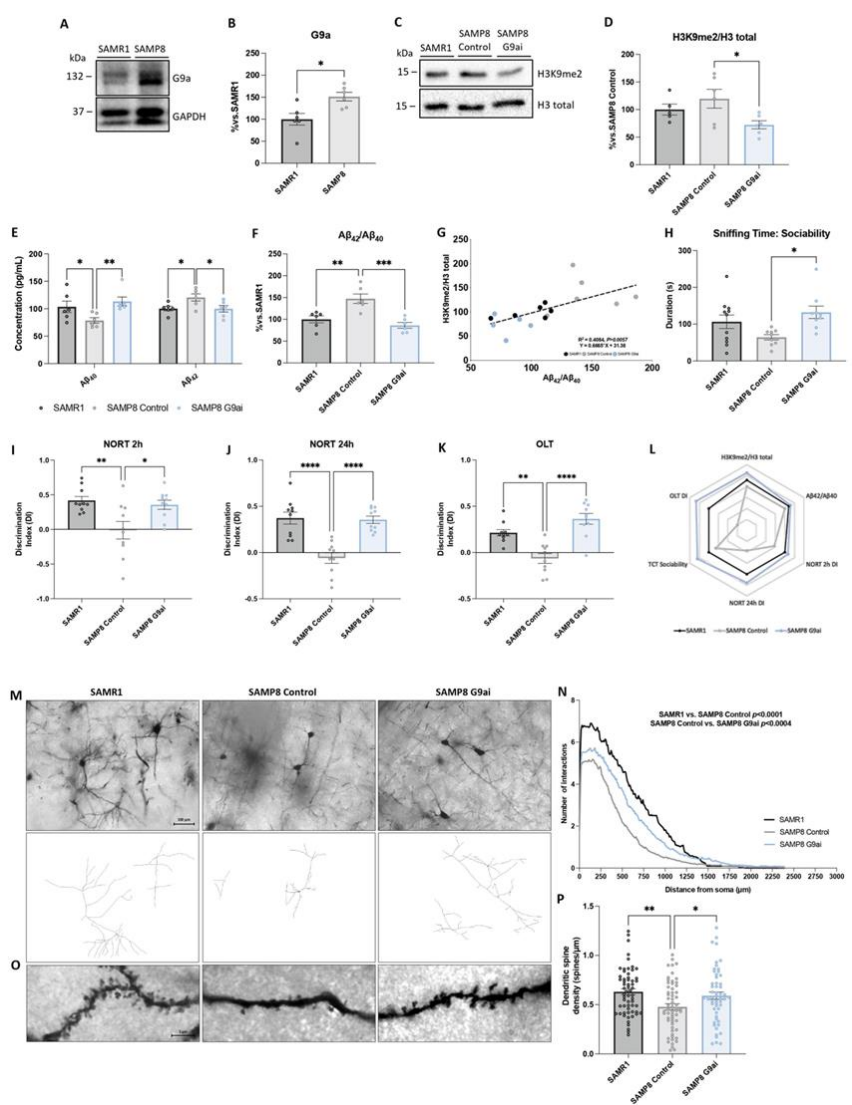
**Pharmacological G9a inhibition leads to a reduction in H3K9me2 and AD hallmarks, restoring dendritic spine density in SAMP8 mice**. (**A**) and (B) Representative WB, and quantification of G9a in the hippocampus of SAMR1 and SAMP8 mice. (**C**) and (D) Representative WB, and quantification of H3K9me2 in SAMR1, SAMP8 and SAMP8 mice treated with G9ai. (**E**) Levels of Aβ40 and Aβ42. (**F**) Ratio of Aβ42/Aβ40 by ELISA. Values presented are mean ± SEM; (N = 3 groups (SAMR1 n = 6, SAMP8 Control n = 6, and SAMP8 G9ai (UNC0642, 5mg/Kg) n = 6); Parametric tests: Student’s t-test analysis, or One-Way ANOVA and post-hoc Tukey’s test, Non-parametric tests: Mann-Whitney test, or Kruskal-Wallis and post-hoc Dunn’s test; *p<0.05; **p<0.01; ***p<0.001). (**G**) Correlation between Aβ42/Aβ40 ratio and H3K9me2 (slope = 0.2071). R2 and p-values are indicated on graphs. For TCT: (H) Sniffing time: sociability with the intruder animal. For NORT: (I) Short-term memory evaluation after 2 h, and (J) long-term evaluation after 24 h in the acquisition trial by Discrimination Index (DI) after exposure of SAMR1, SAMP8 and SAMP8 mice G9ai to novel objects. For OLT: (K) OLT evaluation by DI after changing the location of one object. Values presented are the mean ± SEM; (N = 3 groups (SAMR1 n = 10, SAMP8 Control n = 10, and SAMP8 G9ai (UNC0642, 5mg/Kg) n = 10); One-Way ANOVA and post-hoc Tukey’s test; *p<0.05; **p<0.01; ***p<0.001; ****p<0.0001). (**L**) The polygonal graph presents complete parameters obtained by TCT, NORT, WB and ELISA. (**M**) Representative images and tracings of Golgi-stained neurons from the different experimental groups (scale bar = 100 µm). (**N**) Number of neuronal intersections. Values presented are the mean ± SEM; (N = 2 groups (SAMR1 n = 89, SAMP8 Control n = 98, and SAMP8 G9ai (UNC0642, 5mg/Kg) n = 100); Kruskal-Wallis and post-hoc Dunn’s test; *p*-values represented in the graph). (**O**) Representative images of the spine density of the different experimental groups by Golgi staining. (**P**) Spine density quantification of neurons. Values presented are the mean ± SEM; (N = 3 groups; SAMR1 n= 60, SAMP8 Control n = 60, and SAMP8 G9ai (UNC0642, 5mg/Kg) n = 56 dendrites (of different neurons) were analyzed from 6 mice per group; One-Way ANOVA and post-hoc Tukey’s test; *p<0.05; **p<0.01). Source data are provided as a Source Data file. Data information: Note that experiments in Figs 4N and 5N, and 4P and 5P were performed at the same time, so the values of SAMR1 and SAMP8 Control are the same but the figures have been split in two for the sake of linearity.

Because aging is the main risk factor for AD[66], we determined the G9a protein levels in the SAMP8 strain, a well-established AD model for investigating the key mechanisms of age-related cognitive decline with accelerated aging and generated through phenotypic selection from the AKR/J strain of mice[33, 34]. First, we examined protein levels by WB, including in Senescent-Accelerated Resistant 1 mice (SAMR1)[33], a normal aging control. G9a protein expression was higher in the SAMP8 Control group in comparison with

the SAMR1 Control group (Figs. 4A and B). Likewise, significantly higher levels of H3K9me2 were only observed in the SAMP8 Control group compared to the SAMP8 mice treated with G9ai (Figs. 4C and D). Interestingly, our results showed a significant increase in Aβ_40_ levels in the SAMR1 and SAMP8 G9ai groups compared to the SAMP8 group, whereas for Aβ_42_ levels,

the opposite was observed (Fig. 4E). Therefore, the high Aβ_42_/Aβ_40_ ratio levels in SAMP8 mice were reduced by G9a inhibition (Fig. 4F). Notably, we found a strong positive correlation between H3K9me2 levels and Aβ levels in SAMP8 mice (Fig. 4G). In agreement with AD patients’ data (Fig. 1F), SAMP8 mice data suggest that high H3K9me2 is correlated with increased levels of Aβ in SAMP8, indicating a contribution of the G9a and H3K9me2 to the age-related cognitive decline presented by this AD rodent model.

Aβ levels are associated with behavioral abnormalities and cognitive decline, so we next performed behavioral studies to determine whether pharmacological G9a inhibition could revert them in SAMP8 mice. Firstly, we used the TCT to assess general sociability behavior in mice. In the sociability phase, in all mice groups, the presence of an intruder significantly increased the time spent in the intruder chamber instead of the empty cup chamber on all groups (Supplementary Fig. 7A). Remarkably, we only found a significant increase in the time spent sniffing the intruder mouse in the treated SAMP8 mice, confirming the improvement in social behavior after G9a inhibition (Fig. 4H). To evaluate their working and spatial memories, mice were assessed using the NORT and OLT tests, respectively. During the familiarization phase of the NORT task (Supplementary Fig. 7B) the exploration time was unchanged by G9ai treatment. The task results revealed that SAMP8 treated with G9ai exhibited a significant amelioration in cognitive deficits in both short- and long-term memories (Figs. 4I and J), relative to the SAMP8 Control mice group.

Regarding the OLT results, exploration during the habituation phase (Supplementary Fig. 7C) was unaffected by treatment. The task results showed that SAMP8 treated with G9ai exhibited an increase in the DI compared to the SAMP8 Control group (Fig. 4K), suggesting an improvement in spatial memory. Finally, the polygonal graph depicts differences among SAMR1 and SAMP8 Control, and treated SAMP8 groups in several TCT parameters, the DI of NORT and OLT, and the molecular ratios of H3K9me2 and Aβ_42_/Aβ_40_ (Fig. 4L). In addition, SAMP8 showed a decrease in the number of neuronal intersections compared to the SAMR1 group. However, this was reverted after G9ai treatment in SAMP8, and this increase was most apparent at intermediate and more distal distances from the soma in a Sholl analysis (Figs. 4M and N). Besides, SAMP8 is known pathologically to present a reduction in neuronal spine density[67]. Accordingly, we found a significant increase in spine density in the brains of SAMP8 mice treated with G9ai, as well as in the SAMR1 group, compared to the SAMP8 Control group, showing the potential of G9a inhibition for improving neuronal plasticity (Figs. 4O and P). This data suggests that G9a inhibition promotes beneficial effects on behavior and cognition.

### G9a inhibition increased the GMFB transduction pathway, which in turn mediated the neuroprotective effects in SAMP8 mice.

**Figure 5.**
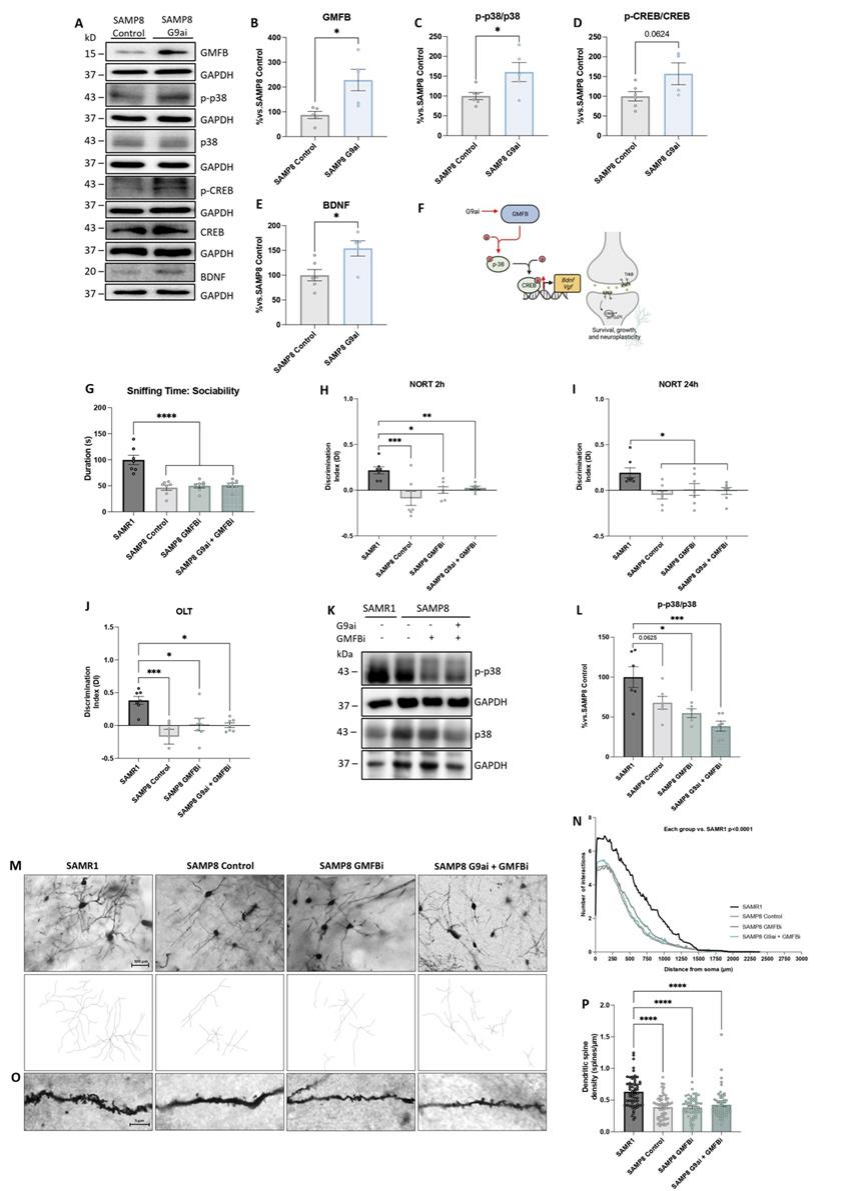
**G9a inhibition increased the GMFB transduction pathway, which in turn mediated the neuroprotective effects in SAMP8 mice**. (**A**) and (B)-(E). Representative WB, and quantifications of GMFB, the p-p38/p38 ratio, the p-CREB/CREB ratio and BDNF. Values presented are the mean ± SEM; (N = 2 groups (SAMP8 Control n = 6, SAMP8 G9ai (UNC0642, 5mg/Kg) n = 6); Student's t-test analysis; *p<0.05; ***p<0.001). (**F**) Representative scheme of the GMFB molecular signaling modulated after G9ai treatment in SAMP8. For TCT: (G) sniffing time: sociability with the intruder animal. For NORT: (H) Short-term memory evaluation after 2 h and (I) long-term evaluation after 24 h in the acquisition trial by DI after exposure to novel objects. For OLT: (J) OLT evaluation by DI after changing the location of one object. (**K**) and (L) Representative WB, and quantification of p-p38/p38 protein levels. Values presented are the mean ± SEM; (N = 4 groups (SAMR1 n = 6-7, SAMP8 Control n = 6-7, SAMP8 GMFBi ((1H-Indazol-4-yl)methanol, 12mg/Kg) n= 6-7, and SAMP8 G9ai (UNC0642, 5mg/Kg) + GMFBi ((1H-Indazol-4-yl)methanol, 12mg/Kg) n = 6-7; Parametric test: One-Way ANOVA and post-hoc Tukey’s test; Non-parametric test: Kruskal-Wallis and post-hoc Dunn’s test; *p<0.05; **p<0.01; ***p<0.001; ****p<0.0001). (**M**) Representative images and tracings of Golgi-stained neurons (scale bar = 100 µm). (**N**) Reduction in the number of neuronal intersections of the SAMP8 control group and both GMFBi treatments compared to the SAMR1 control group. Values presented are the mean ± SEM; (N = 4 groups (SAMR1 n = 89, SAMP8 Control n = 98, SAMP8 GMFBi ((1H-Indazol-4-yl) methanol, 12mg/Kg) n = 93, and SAMP8 G9ai (UNC0642, 5mg/Kg) + GMFBi ((1H-Indazol-4-yl)methanol, 12mg/Kg) n = 99; One-Way ANOVA and post-hoc Tukey’s test). (**O**) Representative images of the spine density by Golgi staining. (**P**) Spine density quantification of neurons. Values presented are the mean ± SEM; (N = 4 groups; SAMR1 n = 60, SAMP8 Control n = 60, SAMP8 GMFBi ((1H-Indazol-4-yl)methanol, 12mg/Kg) n = 60, and SAMP8 G9ai (UNC0642, 5mg/Kg) + GMFBi ((1H-Indazol-4-yl)methanol, 12mg/Kg) n = 60 (of different neurons) were analyzed from 6 mice per group; Kruskal-Wallis and post-hoc Dunn’s test ****p<0.0001). Source data are provided as a Source Data file. Data information: Note that experiments in Figs 4N and 5N, and 4P and 5P were performed at the same time, so the values of SAMR1 and SAMP8 Control are the same but the figures have been split in two for the sake of linearity.

GMFB is implicated in neuronal plasticity. Thus, to further confirm GMFB pathway activation after G9a inhibition in SAMP8, we examined GMFB protein levels and the downstream effector proteins using WB. Strikingly, a significant increase in GMFB was observed in SAMP8 mice treated with G9ai (UNC0642, 5mg/Kg) in comparison with the SAMP8 Control mice (Figs. 5A and B). Furthermore, as GMFB is an enhancer of p38[68] that plays important roles in synaptic plasticity[69], we evaluated the p-p38/p38 ratio and CREB protein levels. A significant increase in p-p38 protein levels was found in the SAMP8 mice treated with G9ai (Figs. 5A and C). Likewise, the p-CREB/CREB ratio was also augmented but did not differ significantly between groups (Figs. 5A and D). Finally, we also evaluated the protein levels of BDNF and its receptor, tropomyosin-related kinase B (TrkB).

Regarding the ratio of protein levels of p-TrkB/TrkB, we observed a significant increase in treated SAMP8 mice compared to the SAMP8 Control group (Supplementary Figs. 8A and B). Accordingly, an increase in BDNF protein levels was found in the SAMP8 G9ai mice (Figs. 5A and E, Supplementary Fig. 8C). These findings suggest, for the first time, that G9a inhibition promotes neuronal plasticity through modulation of the GMFB molecular pathway (Fig. 5F). Thus, to demonstrate that GMFB mediates the neuroprotective effects promoted by G9ai, we evaluated cognition, neuronal plasticity and neuroinflammation status after dual treatment with GMFB and G9a inhibitors in SAMP8 mice. First, we evaluated the effect of GMFB inhibitor (GMFBi) and dual treatment on behavior and cognitive impairment. In TCT, social interaction was significantly decreased in treated SAMP8 group, as it was in the SAMP8 Control group compared to the SAMR1 Control group (Fig. 5G, Supplementary Fig. 9A). Similarly, the DI of short- or long-term memory was lower in GMFBi-treated groups (Figs. 5H and E, Supplementary Fig. 9B). Correspondingly, GMFBi-treated showed a decrease in OLT DI compared to the SAMR1 Control group (Fig. 5J, Supplementary Fig. 9C).

Moreover, since we had previously found that GMFB orchestrated the enhancement of synaptic plasticity by increasing p-p38, this marker was also evaluated. We found a reduction in p-p38/p38 levels in the SAMP8 Control group and the GMFBi-treated groups (Figs. 5K and L). Last but not least, dendritic spine length (Figs. 5M and N) and density length (Figs. 5O and P) in the brain of GMFBi-treated and dual-treated mice showed a similar profile to the SAMP8 group, presenting lower levels compared to the SAMR1 group in both quantifications.

### G9a inhibition ameliorated neuroinflammation, modulating the NF-kB signaling pathway via GMFB.

**Figure 6.**
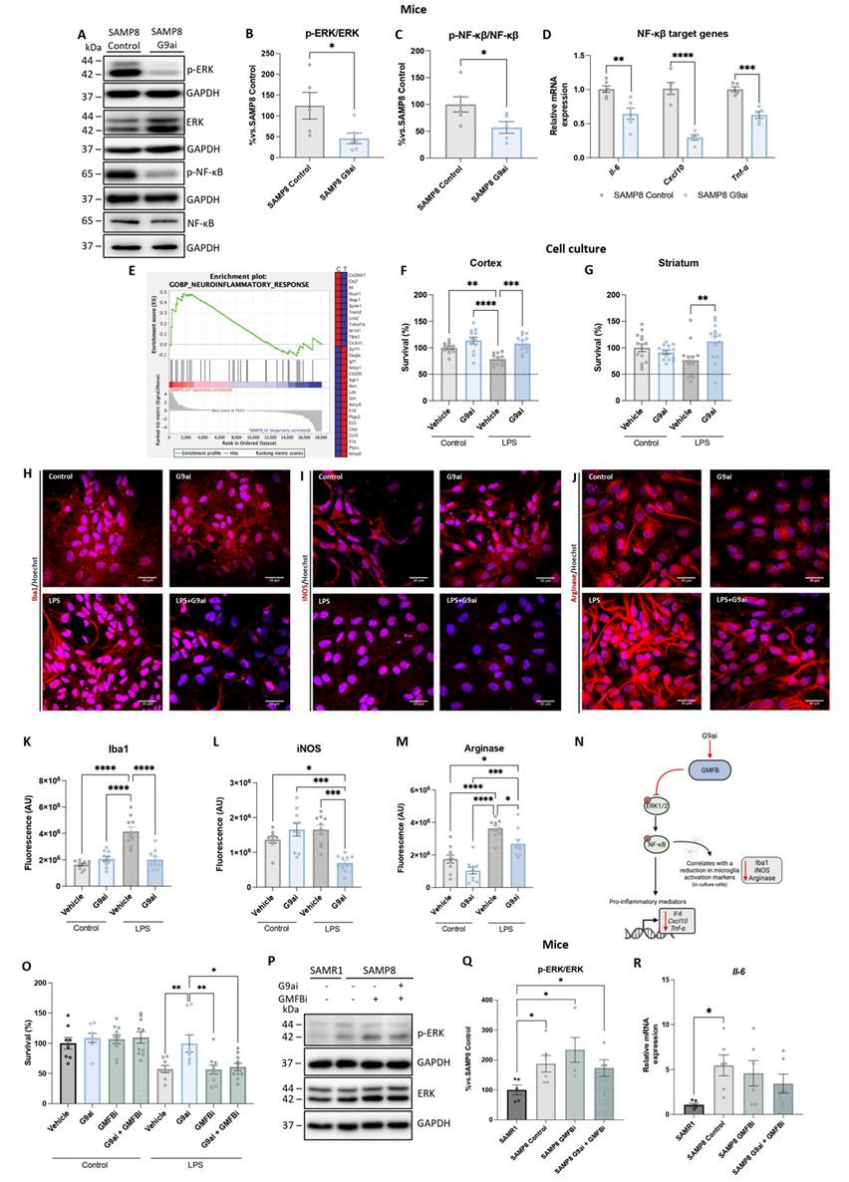
**G9ai treatment reduced the inflammatory markers through the modulation of the NF-κB molecular pathway by GMFB**. (**A**) and (B)-(C) Representative WB, and quantification of p-ERK/ERK, and NF-κB protein levels in SAMP8 mice. (**D**) Representative gene expression of pro-inflammatory markers of *Il-6, Cxcl10*, and *Tnf-α*. Gene expression levels were determined by real-time PCR. Values represented are the mean ± SEM; (N = 2 groups (SAMP8 control n = 6, SAMP8 G9ai (UNC0642, 5mg/Kg) n = 6)); Student’s t-test analysis; **p<0.01; ***p<0.001; ****p<0.0001). (**E**) GSEA plot of neuroinflammatory response up-regulated in SAMP8 control. (**F**) Neuronal viability of primary mixed culture of neurons and microglia from to cortex and (G) striatum stimulated with G9ai (UNC0642, 1 µM) or vehicle, and subsequently treated every 48 h with 1 mM LPS and 200 U/mL IFN-γ for 2 weeks. Values represented are the mean ± SEM; (N= 4 groups (Control Vehicle n = 12; Control G9ai n = 12; LPS Vehicle n = 12; LPS G9ai n = 12)); Two-Way ANOVA and post-hoc Tukey’s test; **p<0.01; ***p<0.001; ****p<0.0001). (**H**) and (K) IHC of Iba1, (I) and (L) iNOS, (J) and (M) Arginase were performed in mice microglial primary cultures treated or not with G9ai (UNC0642, 1 µM). The confocal microscopy images are shown (superimposed sections) in which Iba1, iNOS and Arginase appear as red over Hoechst-stained nuclei. Scale bars = 30 µm. The bars graph shows the number of the red dots/cell (r). The cell nuclei were stained with Hoechst (blue) in all cases. Values presented are the mean ± SEM; (N= 4 groups (Control Vehicle n = 9-10; Control G9ai n = 10; LPS Vehicle n = 10; LPS G9ai n = 10)); One-Way ANOVA and post-hoc Tukey’s test; *p< 0.05; ***p<0.001; ****p<0.0001). (**N**) Representative scheme of the proposed neuroinflammatory pathway modulated after G9ai treatment in SAMP8. (**O**) Neuronal viability was quantified in primary mixed culture of neurons and microglia was stimulated with GMFBi ((1H-Indazol-4-yl)methanol, 0.67µM) or G9ai (UNC642, 1µM) + GMFBi ((1H-Indazol-4-yl)methanol, 0.67µM), and subsequently treated every 48 h with 1 mM LPS and 200 U/mL IFN-γ for 2 weeks; Values presented are the mean ± SEM; (N = 8 groups (Control Vehicle n = 8; Control G9ai n = 8; Control GMFBi n = 10, Control G9ai + GMFBi n = 10, LPS Vehicle n = 8, LPS G9ai n = 8, LPS GMFBi n = 10, and LPS G9ai + GMFBi n = 10; Kruskal-Wallis and post-hoc Dunn’s test; and Mann-Whitney test;*p<0.05; **p<0.001). (**P**) and (Q) Representative WB, and quantification of p-ERK/ERK protein levels. (**R**) Representative gene expression of pro-inflammatory markers for *Il-6*. Gene expression levels were determined by real-time PCR. Values presented are the mean ± SEM; (N = 4 groups (SAMR1 n = 5-6, SAMP8 Control n = 6, SAMP8 GMFBi ((1H-Indazol-4-yl) methanol, 12mg/Kg) n = 5-6, and SAMP8 G9ai (UNC0642, 5mg/Kg) + GMFBi ((1H-Indazol-4-yl)methanol, 12mg/Kg) n = 6); One-Way ANOVA and post-hoc Tukey’s test*p<0.05). Source data are provided as a Source Data file. Data information: Note that experiments in Supplementary Fig. 6B and Fig. 6D were performed at the same time, so the values regarding gene expression of *Cxcl10* and *Tnf-α* are the same but the figures have been split in two for the sake of linearity.

We have previously shown an increased neuro-inflammatory process in SAMP8 [70]. Interestingly, GMFB may also modulate neuroinflammation via the extracellular signal-regulated kinase (ERK) and nuclear factor kappa B (NF-κB) axis [32]. Of note, the RNA-seq showed a reduction in the activity of the NF-κB signaling pathway (Fig. 3G) and an increase in the anti-inflammatory cytokine *Il-37* in G9ai-treated SAMP8 mice (Supplementary Figs. 4A and B), which suggests that treatment with G9ai could reduce the levels of neuroinflammation in the SAMP8 model. To demonstrate this, we evaluated neuroinflammation status after G9ai treatment. NF-κB is a transcription factor related to the inflammatory response and a master commander in the expression of pro-inflammatory genes, and its signaling is an important mediator of brain inflammation in AD[71]. To investigate the activation levels of NF-κB in the treated SAMP8 mice, we first evaluated the levels of ERK protein, which plays a critical role by inducing NF-κB expression. Strikingly, a significant reduction in the ratio of p-ERK/ERK ratio was found in G9ai-treated SAMP8 mice (Figs. 6A and B), correlating with the ratio of p-NF-κB/ NF-κB protein levels (Figs. 6A and C). Next, we assessed the gene expression of some pro-inflammatory NF-κB targets, such as *interleukin-6 (Il-6), Cxcl10*, and *Tnf-α*. Significantly lower expression of these pro-inflammatory genes was observed in G9ai-treated SAMP8 mice relative to the SAMP8 mice group (Fig. 6D). Additionally, a GSEA analysis demonstrated a reduction in the enrichment of genes associated with the neuroinflammatory response in G9ai-treated SAMP8 mice (Fig. 6E).

Analysis of cell death in a primary mixed culture of neurons and microglia from the cortex and striatum revealed that pretreatment with G9ai significantly decreased cell death after activation with lipopoly-saccharide (LPS) and interferon-gamma (IFN-γ) from the cortex and striatum (Fig. 6F and G). Thus, LPS and IFN-γ induced microglial activation, detected as an increase in fluorescence after one week of treatment. Interestingly, G9ai pretreatment blocked this effect (Figs. 6H-M). Taken together, these data suggest that G9ai treatment enhances neuroinflammation, possibly via GMFB orchestrating transcriptional changes in the NF-κB signaling pathway (Fig. 6N). Our results showed that G9ai treatment was able to reduce these inflammatory markers mainly in pathological conditions (LPS), and thus, we wondered whether GMFB plays an important role in this modulation.

Notably, GMFB is an enhancer of p38, and also orchestrated a reduction in inflammation through the ERK pathway[68, 72]. We thus analyzed cell death by treating cells with a GMFBi, and the combination of a G9ai and a GMFBi. Under normal conditions, no difference was observed between the treated groups and the Control group. However, under LPS exposure, cell survival in those groups treated with a GMFBi was reduced in the same way as in the Control group, the G9ai-treated group being the only one with a significant increase in survival (Fig. 6O). On the other hand, GMFBi treatment increased p-ERK/ERK levels (Figs. 6P and Q). Further investigation of the role of GMFB led us to evaluate the levels of *Il-6* as a pro-inflammatory marker, and an increased in its gene expression levels was observed in the

SAMP8 GMFBi group, in the SAMP8 dual treatment group and in the SAMP8 Control group, relative to the SAMR1 group (Fig. 6R). Therefore, we demonstrated that GMFB modulates changes in the neuroinflammation status after G9a inhibition.

### GMFB is methylated by G9a triggering neurodegeneration.

**Figure 7.**
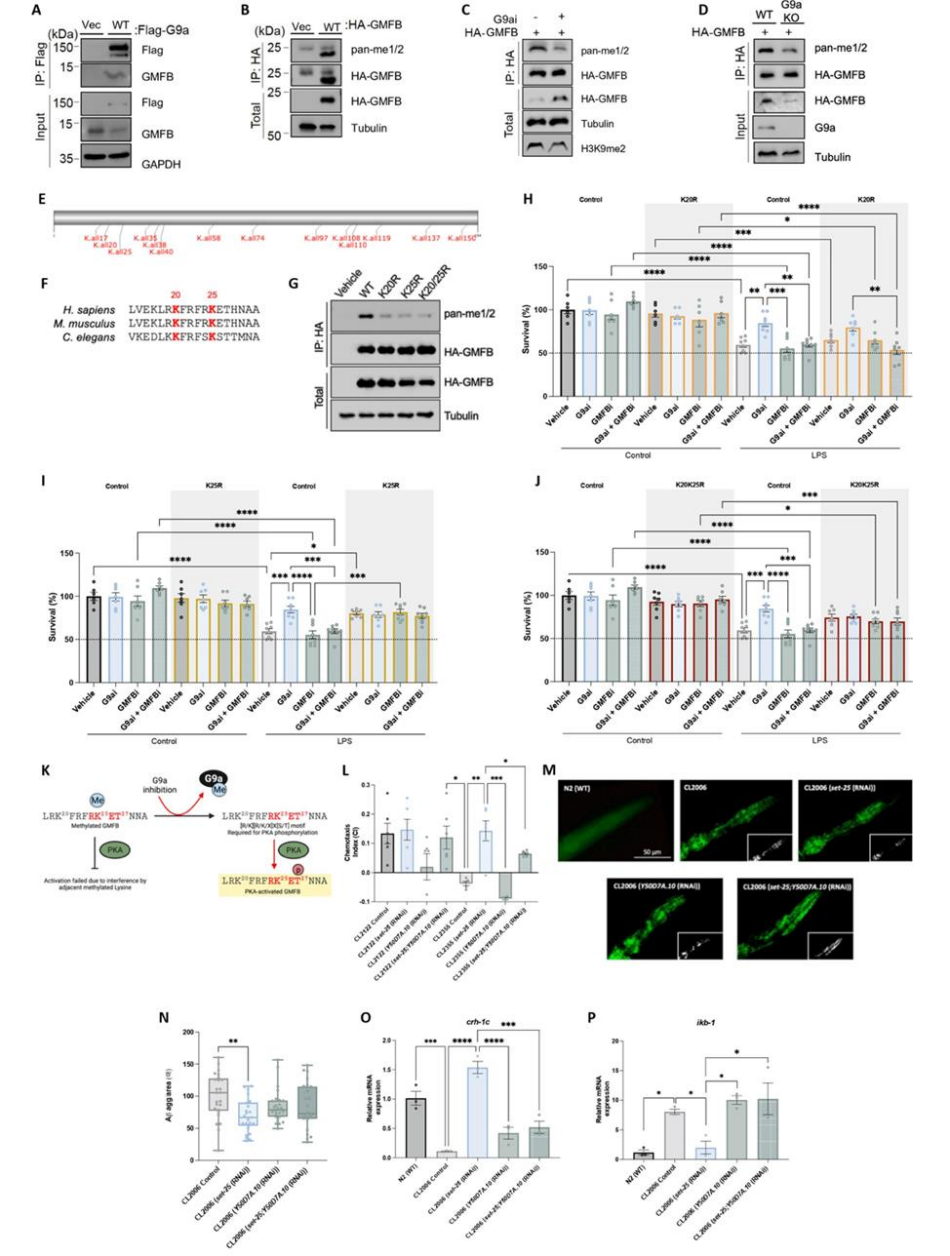
**GMFB is lysine methylated by G9a triggering neurodegeneration**. (**A**) Co-IP assay was performed to show that Flag-G9a interacts with GMFB in HEK 293T cells. (**B**) GMFB is capable of being methylated. WB was performed to examine the methylation state of GMFB in HEK 293T cells. (**C**) Inhibition of G9a decreased methylated level of GMFB. (**D**) G9a-KO decreased methylated level of GMFB. (**E**) Predictive lysine methylation sites in GMFB by GSP-MSP method. (**F**) Sequence alignment of human GMFB with selected homologs. (**G**) Methylation state of the different GMFB sites by G9a in the generated HEK 293T cell lines (K20R, K25R, and 2KR mutants). (**H**) Neuronal viability of K20R-KD, (I) K25R-KD, and (J) 2KR-KD HEK 293T cells with G9ai (UNC0642, 1 µM), GMFBi ((1H-Indazol-4-yl)methanol, 0.67µM), G9ai + GMFBi, or vehicle, and subsequently treated every 48 h with 1 mM LPS (N = 32 groups (Control Vehicle n = 6, Control G9ai n= 7, Control GMFBi n = 6, Control G9ai + GMFBi n = 6, LPS Vehicle n = 8; LPS G9ai n = 8; LPS GMFBi n = 8; LPS G9ai + GMFBi n =8, Control Vehicle K20R-KD n = 6, Control G9ai K20R-KD n = 6, Control GMFBi K20R-KD n = 6, Control G9ai + GMFBi K20R-KD n = 6, LPS Vehicle K20R-KD n = 8; LPS G9ai K20R-KD n = 8, LPS GMFBi K20R-KD n = 8, LPS G9ai + GMFBi K20R-KD n = 8, Control Vehicle K25R-KD n = 6, Control G9ai K25R-KD n = 6, Control GMFBi K25R-KD n = 6, Control G9ai + GMFBi K25R-KD n = 6, LPS Vehicle K25R-KD n = 7, LPS G9ai K25R-KD n = 7, LPS GMFBi K25R-KD n = 8, LPS G9ai + GMFBi K25R-KD n = 8, Control Vehicle K20RK25R-KD n = 6, Control G9ai K20RK25R-KD n = 6, Control GMFBi K20RK25R-KD n = 6, Control G9ai + GMFBi K20RK25R-KD n = 6, LPS Vehicle K20RK25R-KD n = 7, LPS G9ai K20RK25R-KD n = 8, LPS GMFBi K20RK25R-KD n = 8, and LPS G9ai + GMFBi K20RK25R-KD n = 8); Two-Way ANOVA and post-hoc Tukey’s test; *p<0.05; **p<0.01;***p<0.001; ****p<0.0001). (**K**) Representative scheme of the hypothesis proposed for the activation of the GMFB. (**L**) CI calculated for CL2122 (non Aβ strain), CL2355 (neuronal Aβ strain), and double and/or single mutant in CL2355 and CL2122. Values presented are CI mean ± SEM; (N = 8 groups (CL2122 Control n = 6, CL2122 (*set-25* (RNAi)) n = 6, CL2122 (*Y50D7A.10* (RNAi)) n = 4, CL2122 (*set-25;Y50D7A.10* (RNAi)) n = 6, CL2355 Control n = 5, CL2355 (*set-25* (RNAi)) n =6, CL2355 (*Y50D7A.10* (RNAi)) n = 5, and CL2355 (*set-25;Y50D7A.10* (RNAi)) n = 6); Each biological replicates with at least 200 worms for each analysis; One-Way ANOVA and post-hoc Tukey’s test; *p<0.05; **p<0.01). (**M**) and (N) Representative images from each group and quantification of Thioflavin S-positive particles in the head region of CL2006 Control, CL2006 (*set-25* (RNAi)), CL2006 (*Y50D7A.10* (RNAi)) and CL2006 (*set-25;Y50D7A.10* (RNAi)). Values presented are the mean ± SEM. (N = 4 groups (CL2006 Control n = 25, CL2006 (*set-25* (RNAi)) n = 26, CL2006 (*Y50D7A.10* (RNAi)) n = 26, CL2006 (*set-25;Y50D7A.10* (RNAi)) n = 25); Kruskal-Wallis and post-hoc Dunn’s test; **p<0.01). (**O**) and (P) Representative gene expression of chr-1c, and ikb-1 in C. elegans. Gene expression levels were determined by real-time PCR. Values presented are mean ± SEM. (N = 5 groups (N2(WT) n = 3, CL2006 Control n = 3, CL2006 (*set-25* (RNAi)) n = 3, CL2006 (*Y50D7A.10* (RNAi)) n = 3, CL2006 (*set-25;Y50D7A.10* (RNAi)) n = 3; Each biological replicate with at least 300 worms in each group); One-Way ANOVA and post-hoc Tukey’s test; *p<0.05;***p<0.001;***p<0.0001). Source data are provided as a Source Data file.

We generated an exogenous G9a construct to perform the co-immunoprecipitation assay. Either empty vector or a Flag-tagged human G9a construct were transfected into HEK 293T cells. The interaction of G9a and GMFB was detected in the Flag-G9a-transfected sample but not in the Control sample (Fig. 7A). Therefore, as G9a can interact with GMFB, and inhibition of G9a affects GMFB functions, we speculate that G9a might methylate GMFB directly. To test this hypothesis, we first examined whether GMFB could be methylated in cells. HA-tagged GMFB was transfected into HEK 293T cells, and protein was enriched by immunoprecipitation using anti-HA antibody. As expected, methylated forms of GMFB were observed using the anti-pan methyl lysine antibody, recognizing both mono and dimethyl substrates (Fig. 7B). To determine whether G9a is the methyltransferase responsible for GMFB methylation, two approaches were utilized. A G9ai (BIX01294, 10 µM) was added to HEK 293T cells that express HA-GMFB and incubated for 3 days. Upon drug incubation, we observed lower levels of methylated GMFB compared with that in DMSO-treated cells (Fig. 7C). Consistently, G9a knockout (KO) also significantly reduced the methylated levels of GMFB compared with that of WT cells (Fig. 7D).

To further identify the potential methylation sites of GMFB by G9a, we first search a consensus methylation site encompassing RK/ARK, which has been indicated to be a preferred target by G9a [13]. The GPS-MSP method was used to predict all possible methylation sites in GMFB (Fig. 7E, Supplementary Table 9) [73, 74]. In fact, only two proposed residues of GMFB potentially fit the above criteria to be targeted by G9a: Lys20 (K20) and Lys25 (K25) (Fig. 7F). To validate whether they can be methylated by G9a, HA-tagged constructs bearing either a single lysine-to-arginine (K20R, K25R) mutation or double mutations (K20RK25R, referred as 2KR) were generated, and their methylation status by G9a were examined by immunoprecipitation of HA-GMFB followed by WB with anti-pan methyl antibody in HEK 293T cells. Interestingly, we noticed that either K20R, K25R or 2KR mutant significantly reduced methylation levels of GMFB, compared with that of WT construct (Fig. 7G). The methylation signals were specific, as overexpression of empty vectors in cells did not detect any methylated bands (Fig. 7G). Of note, the remaining methylation level of 2KR mutant suggested that there may exist other methylation sites. Nevertheless, we found that K20R and K25R are the two main sites methylated by G9a.

We next sought to investigate the neuroprotective effect of pharmacological G9a inhibition on GMFB binding sites by evaluating cell survival upon LPS exposure in the different cell lines generated. On the one hand, we observed that when we KD K20R in HEK 293T cells, cell viability was only restored after LPS in the G9ai treated group (Fig. 7H). In contrast, the vehicle group as well as the GMFBi treated group did not show any improvement in cell viability, and even exhibited levels similar to those of the vehicle group of control cells. As expected, the group with both treatments showed no cytoprotective effect (Fig. 7H). On the other hand, when K25 of GMFB was mutated in R in the cells, both in the vehicle group and in the G9ai-treated group, a recovery of cell viability was observed upon LPS exposure (Fig. 7I). All together these results agreed with the levels observed in the double-mutated cells (2KR) (Fig. 7J). In conclusion, these findings suggest that the neurodegenerative role of G9a as a GMFB suppressor would mainly rely on methylation of the K25 position of GMFB, and thus its pharmacological inhibition removes this methylation promoting neuroprotective effects. Nevertheless, methylation at the K20R position of GMFB by G9a is thought to play a secondary role in the neuroprotective effect.

It is also relevant to note that the activation of GMFB depends on protein kinase A (PKA). GMFB phosphorylated by PKA enhances p-38 activity (almost 60-folds) and inhibits ERK1/ERK2 (IC_50_ = 3 nM) [68, 75, 76]. Interestingly, the consensus motif in PKA substrates (96%) is [R/K][R/K/X]X[S/T][77] and the sequence analysis of GMFB shows that the motif only fits LRKFRFRK25ET27 sequence where the PKA phosphorylation site (T27) is neighboring to G9a methylation site (K25). Moreover, it has been reported before [78] that methylated lysine interferes with the subsequent phosphorylation of GMFB by PKA. Therefore, GMFB gets phosphorylated by PKA under G9a inhibition to activate and perform its subsequent functions (Fig. 7K).

Finally, to demonstrate the potential effects of G9a on GMFB, we generated a KD of *Y50D7A.10* (ortholog of human *Gmfb*) in CL2355 *C. elegans* strain, using the RNAi approach. As expected, our results showed that CL2355 (*set-2*5; *Y50D7A.10* (RNAi)) and CL2355 (*Y50D7A.10* (RNAi)) were not able to restore impaired learning and memory (Fig. 7L, Supplementary Fig. 10). In addition, we showed that the single or double KD of *Y50D7A.10* in the CL2006 *C. elegans* strain failed to reduce Aβ aggregation when compared to the CL2006 Control group (Figs. 7M and N). According to the above results in mice, we also evaluated the potential effects of GMFB on several well-established target genes of G9a. The gene expression of the *crh-1c* decreased (Fig. 7O), while that of *ikb-1* increased (a homolog of human IκB) (Fig. 7P), in those groups that presented *Y50D7A.10*-KD in the same way as in the CL2006 Control group, compared to the N2 group and G9ai-treated group. Thus, *Y50D7A.10*-KD in *C. elegans* reversed all cognitive and behavioral improvements exhibited by the *set-25*-KD strain, indicating a possible role of GMFB in the beneficial effects of G9ai treatment. And for the first time, these results support the hypothesis that G9a interacts with GMFB (regulating it secondarily) to promote neurodegeneration.

## DISCUSSION

In the present study, we unraveled an undescribed molecular pathway based on the GMFB transcriptional repression controlled by G9a, which in turn mediates neuroprotection after G9a inhibition. Secondarily, we presented, for the first time, that G9a directly methylates GMFB. We also demonstrated the relevance of G9a and its repressive histone mark H3K9me2 in AD patients, which correlates with an increased levels of the Aβ_42_/Aβ_40_ ratio, an important hallmark of the disease. First, we used the RNAi method in *C. elegans* as a much cleaner approach than pharmacological inhibition to reduce the off-target effect and elucidate how G9a/SET-25 contributes to the AD pathogenesis. Then, in *C. elegans* AD strains, we showed Aβ aggregation and epigenetic alterations similar to those observed in AD patients that were reversed after *set-25-*KD. These findings confirmed our premise concerning the contribution of G9a to AD-like pathology.

To support our hypothesis that the G9a inhibition would be a promising therapy for AD, we performed an exploratory ChIP-seq analysis, revealing an H3K9me2 enrichment the distal and promoter regions, suggesting a transcriptional regulation by G9a. In line with our findings, it has been reported that TG2576 mice presented high levels of Aβ_40_ and Aβ_42_ with increased H3K9me2 levels in the cortex and hippocampus [17]. This suggests that treatment with G9ai could restore neural alterations associated with Aβ oligomers by reducing H3K9me2 levels. Consistent with these results, the RNA-seq data revealed alterations in molecular processes associated with chromatin methylation and H3K9me2, which indicates the participation of the proteins associated with the histone mark H3K9me2. This would support the results of the H3K9me2 ChIP-seq analysis in AML12 cells. Thus, based on these findings and previous reports [7], we suggest that G9a inhibition could improve cognition in AD patients.

**Figure 8.**
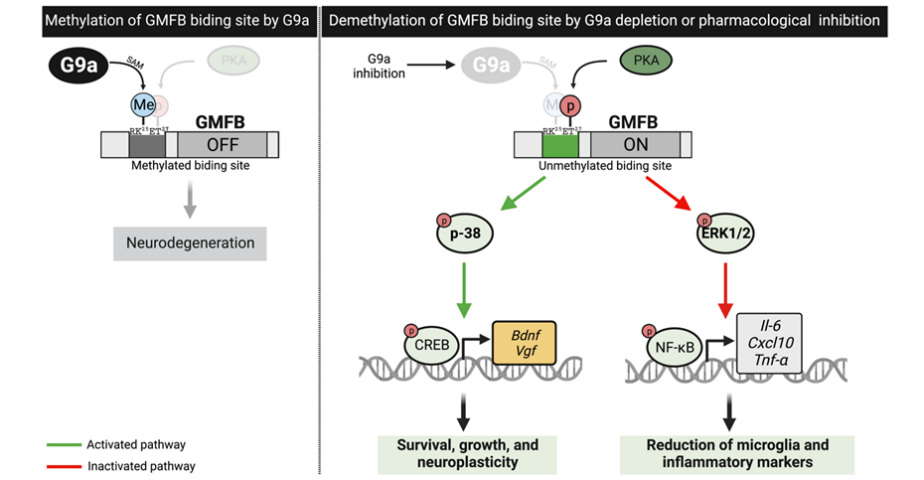
Scheme of the undescribed molecular mechanism, controlled by G9a-mediated methylation of GMFB, which in turn mediates the neuroprotection after G9a inhibition.

In this respect, we demonstrated in SAMP8 mice similar specific epigenetic modifications in G9a and H3K9me2 to those observed in AD patients. Furthermore, we found that the levels of Aβ_42_/Aβ_40_ ratio decreased after G9ai in SAMP8 mice. Although, we found increased Aβ_40_ levels in both SAMR1 and SAMP8 G9ai groups, it is well established that this amyloid fragment inhibits Aβ deposition *in vivo* [79], and therefore this would partly explain the mechanism by which Aβ aggregation could be reduced after G9a inhibition. However, the mechanism is not explored in depth in this work, but the reduction of Aβ load after G9ai could be as a consequence of direct modulation of non-amyloidogenic pathway genes, reducing cognitive impairment. Furthermore, other molecular pathways that are regulated by G9a could also be responsible, such as autophagy [80] and neuroinflammation [12], among others. More importantly, G9ai treatment restored cognitive status in SAMP8 mice in the same way as is reported in 5XFAD mice [3, 12]. Besides, aggressive behavior has a direct influence on social interaction, and in the same line, the inhibition of G9a has been associated with a decrease in anxiety-like behaviors in adult male mice[19]. Nevertheless, the beneficial effect of G9ai treatment on social performance has not been described previously. Thus, for the first time, we demonstrate that G9a inhibition improved social behavior in SAMP8 mice. Consistently, it has been reported that inhibitors of KTMs lead to the remarkable restoration of cognitive behaviors, such as recognition memory, spatial memory, and working memory [19, 81].

Aberrant gene expression is associated with epigenetic alterations [2], and although some relevant *in vivo* studies are being performed, few have described novel modulated pathways after G9a inhibition in detail [64]. Therefore, one of the main interests of this study was to identify unknown non-canonical pathways by which G9a might act, contributing to transcriptomic changes that lead to neuroprotection. Of note, we focused on validating the GMFB up-regulation in SAMP8 mice after G9ai treatment, because GMFB activation induces the synthesis induction of several important proteins in brain function and promotes neurotrophins production, thus suggesting a role in neuronal growth and regeneration [82], and is correlated with learning and memory formation. Strikingly, GMFB appears to trigger activation of p38 MAPK, followed by activation of the transcription factor CREB, which up-regulates BDNF expression levels, thus confirming the pathway activation in SAMP8 mice [68, 83]. It is noteworthy that p38 MAPK is highly expressed in brain regions that are crucial for learning and memory, and it has been reported that its activation is necessary for BDNF production in the rat hippocampus [69]. In addition, consistent with our results, the generation of GMFB-KO mice revealed its importance in normal cognitive functions since its loss promoted pathological neuroinflammatory disease progression[84]. However, no increase in the expression of neurotrophins, such as BDNF and NGF, expression was observed in the cerebellum of GMFB-KO mice in an *in vivo* study, which displayed impaired of motor and learning skills [84, 85]. Importantly, the activation of p38 MAPK might be correlated with morphological changes in neuronal dendritic spines [86]. Remarkably, Golgi staining analysis revealed an increase in neuronal complexity, which correlated with better cognitive performance after G9ai treatment as well as better cognitive performance in SAMP8 mice. Importantly, therapeutic strategies that would reduce neurodegeneration, and actively promote neuro-regeneration, to better functional phenotype improvement, are of great interest. In this context, G9a has been reported to play a role in axon formation, controlling the expression of genes associated with cAMP and Ca^2+^-dependent signaling[64]. Although our results on the GMFB-related signaling pathway were beneficial in SAMP8 mice, there are conflicting findings on how GMFB expression is triggered during neuroinflammation and neurodegeneration[29]. As mentioned earlier, GMFB activation promotes NF-κB expression, thus inducing an inflammatory response[87]. Hence, the precise molecular mechanism by which GMFB is involved in neurodegeneration remains unclear[30]. For this reason, the assessment of pro-inflammatory markers was of great importance in this work.

A wealth of evidence confirms the link between increased levels of inflammatory markers and AD pathogenesis, suggesting that neuroinflammation plays a relevant role in neurodegeneration. At first glance, up-regulation of GMFB might compromise the neuroinflammatory state; however, according to our validated *in vivo* results, we can confirm that ERK-NF-κB orchestrates a decrease in pro-inflammatory NF-kB target genes in AD progression in response to G9a inhibition. The ERK pathway triggers the proinflammatory state, thus inducing the NF-κB [88]. Although the relationship between NF-κB and G9a has already been described during an immune response [89], it is still unknown in the context of neuroinflammation [12, 90]. Consistent with our results, clinical research has shown a positive correlation between CXCL10 and cognitive impairment in AD patients [91]. Furthermore, IL-6 is a pleiotropic inflammatory cytokine secreted by activated glia in the CNS, and is involved in the aging process and the pathogenesis of neurodegeneration [92]. Besides, the pro-inflammatory cytokine TNF-α exacerbates both Aβ and tau pathology. Thus, anti-inflammatory strategies demonstrated amelioration of cognitive function in rodent models of AD [93], consistent with the results of G9ai treatment carried out in SAMP8 mice. Interestingly, clinical imaging studies reported that neuroinflammation in AD patients was characterized by negative correlations between microglial activation and structural integrity or functional activity in the hippocampus of AD patients. As shown by the *in vitro* model of inflammation induced by LPS treatment, the inhibition of G9a reduced microglial-specific marker expression, including Iba-1, iNOS, and Arginase expression. In line with our results, several studies identified increases in Iba-1 expression in AD patients compared with control groups, suggesting that this protein is crucial for activating microglial processes [94]. Moreover, iNOS is mainly expressed by microglia that are activated in different pathological situations, its overexpression being associated with the induction of neuronal death [95]. Thus, these results of lower levels of reactive microglial markers after G9ai treatment *in vitro* reaffirmed the *in vivo* results mentioned above and previously reported in an early-onset AD mouse model by us [12] .

Based on the above, we wondered whether GMFB plays a key role in this modulation. Therefore, we analyzed cell death by treating primary culture cells with a GMFBi, and the combination of a G9ai and a GMFBi. Under normal conditions, there were no differences between treated groups and the control group. Interestingly, we found a reduction in cell survival after neuroinflammatory challenge in those groups treated with a GMFBi compared to the G9ai-treated cell group, suggesting the involvement of the GMFB in this beneficial effect under LPS insult. To the best of our knowledge, this is the first study suggesting this undescribed mechanism and showing the relationship between G9a and GMFB.

We subsequently set out to go further and demonstrate that the beneficial effect following G9a inhibition *in vivo* was orchestrated by increased GMFB protein levels. Thereby, GMFBi treatment and co-treatment with a GMFBi plus G9ai were carried out in SAMP8 mice. Overall, both the SAMP8 group treated with GMFBi and the group treated with both inhibitors showed the same pattern in behavioral tests as that exhibited by the untreated SAMP8 mice. Next, given that GMFB is an enhancer of p38 and inhibitor of ERK, we evaluated their protein levels. In support of our proposed mechanism, our findings showed that SAMP8 groups treated with GMFBi (either alone or in combination) showed a reduction in the p-p38/p38 ratio levels. Accordingly, we found that there was no difference in cortex dendritic arborization and spine density were observed in GMFBi and dual treatment in SAMP8 mice relative to the SAMR1 group, and thus, both treated groups presented the same levels as the untreated SAMP8 group. On the other hand, an increase in the ERK-NF-kB signaling pathway and a subsequent increase in the NF-kB target gene *Il-6* in SAMP8 groups treated with GMFBi (either alone or in combination) were found, thus perpetuating the neuroinflammation in these treated SAMP8 groups. In view of the above, these findings indicate that the neuroprotective effect of G9a inhibition was mediated mainly by GMFB, which increases p38 MAPK activity, favoring synaptic plasticity while decreasing neuroinflammation through the ERK pathway.

In the next phase of our study, the direct protein-protein interaction between G9a and GMFB was evaluated to confirm our findings described above. As aforementioned, G9a can also methylate non-histone proteins [96]. Furthermore, recently it has been proposed a novel direction for the study of GMFB biofunction after being SUMOylated at multiple sites by SUMO1 [23]. But nothing has been described about the link between GMFB and methylation. Our data here observed a remarkable decrease in the methylation level of GMFB upon inhibition or removal of G9a, leaving relatively small amounts of methylated GMFB were. These data suggest that G9a is not the sole methyltransferase for GMFB methylation. It has been reported that G9a forms a heterodimer with G9a-like protein (GLP), and that both can catalyze histone H3K9 mono- and di-methylation. Thus, it is likely that GLP may also contribute to the methylation of GMFB. Further investigation is needed to examine this possibility. Regardless of this, our data supports that G9a is indeed required for GMFB methylation *in vivo*. Going deeper into this hypothesis, we decided to review the literature and found that GMFB has two sites, K20R and K25R, which may be preferentially targeted by G9 methylation [13]. Thus, we generated KDs of K20R, K25R, and 2KR cells to validate G9a's ability to methylate these sites. Our findings suggest that K20R and K25R are the top two sites methylated by G9a. Next, we evaluated the neuroprotective role between the GMFB and G9a binding sites by evaluating cell survival upon LPS exposure in the generated cell lines. Strikingly, our findings suggest that G9a's neurodegenerative role is mainly driven by its methylation at position K25 of GMFB. Conversely, demethylation at position K20R of GMFB by G9a may play a secondary role in the neuroprotective effect. Therefore, pharmacological inhibition of G9a would enhance neuroprotective effects by demethylating this position (K25R). This demethylation at position K25 is required for the phosphorylation of GMFB by PKA, an essential step for GMFB activation. Furthermore, it is noteworthy that GMFB phosphorylated by PKA increases p38 activity and inhibits the ERK pathway [68, 75, 76], as we observed in the SAMP8 group treated with G9ai.

Finally, we generated the *Y50D7A.10*-KD in *C. elegans* AD strains. Consistent with our previous results, we found that CL2355 (*Y50D7A.10* (RNAi)) and CL2355 (*set-25; Y50D7A.10* (RNAi)) were not able to restore cognitive impairment. Likewise, CL2006 (*Y50D7A.10* (RNAi)) and CL2006 (*set-25; Y50D7A.10* (RNAi)) showed no Aβ reduction. Moreover, single, and double KD of *Y50D7A.10* in CL2006 strains presented lower levels of *chr-1c*, and higher levels of *ikb-1*, both well-established target genes of G9a. Therefore, our findings demonstrate that G9a interacts with GMFB, and that the inhibition of G9a is crucial to promote the neuroprotective mechanism through GMFB.

Here, we have highlighted a novel G9a-mediated mechanism. Upon G9a inhibition, this mechanism exhibited two levels of regulation, activating GMFB and modulating its function, to lead to neuroprotection. Hence, we found that the beneficial effects of *set-25*-KD or pharmacological G9a inhibition on cognition, neuronal plasticity and neuroinflammation were abrogated by GMFB inhibition, pinpointing GMFB as a master key in the neuroprotective effects of G9ai. Finally, we also provided evidence showing that repressive regulation of GMFB by G9a methylation inhibits the activation of GMFB triggering neurodegeneration (Fig. 8).

## Supplementary Materials

The Supplementary data can be found online at: www.aginganddisease.org/EN/10.14336/AD.2023.0424-1.



## Data Availability

All data are available in the Source Data File.
