# Supplementary material for: G9a Inhibition Promotes Neuroprotection through GMFB Regulation in Alzheimer’s Disease
Source: Aging Dis. 2024 Feb 1;15(1):311–37. doi: 10.14336/AD.2023.0424-2 (PMC10796087; doi:10.14336/AD.2023.0424-2)
Supplement: Supplementary file 1 [file AD-15-1-311-s.pdf]

SUPPLEMENTARY DATA

G9a Inhibition Promotes Neuroprotection through GMFB Regulation in Alzheimer's Disease

Aina Bellver-Sanchis, Qizhi Geng, Gemma Navarro, Pedro A. Ávila-López, Júlia Companys-Alemany, Laura Marsal-García, Raquel Larramona-Arcas, Lluisa Miró, Anna Perez-Bosque, Daniel Ortuño-Sahagún, Deb Ranjan Banerjee, Bhanwar Singh Choudhary, Francesc X Soriano, Coralie Poulard, Mercè Pallàs, Hai-Ning Du, Christian Griñán-Ferré

© 2023. Bellver-Sanchis A. et al. Published online at http://www.aginganddisease.org/EN/10.14336/AD.2023.0424-2

SUPPLEMENTARY DATA

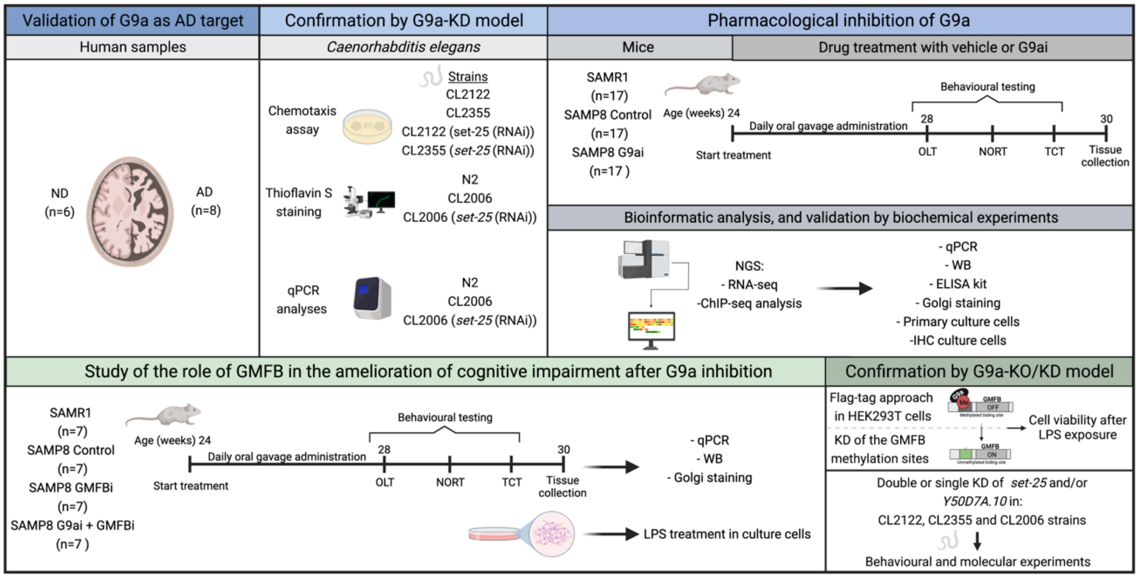

Supplementary Figure 1. Scheme of experimental procedures for *in vitro* and *in vivo* experiments.

© 2023. Bellver-Sanchis A. et al. Published online at http://www.aginganddisease.org/EN/10.14336/AD.2023.0424-2

SUPPLEMENTARY DATA

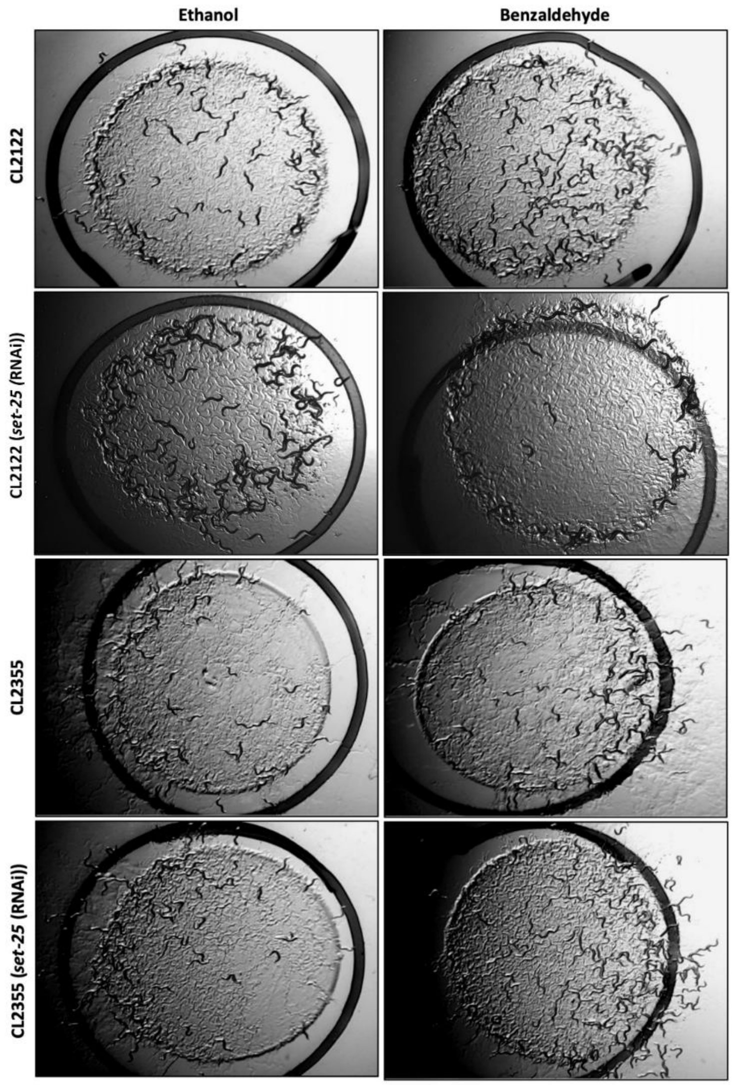

Supplementary Figure 2. Representative image of worms within the attractant (right) or control (left) spot on the chemotaxis assay plates of the different groups.

© 2023. Bellver-Sanchis A. et al. Published online at http://www.aginganddisease.org/EN/10.14336/AD.2023.0424-2

SUPPLEMENTARY DATA

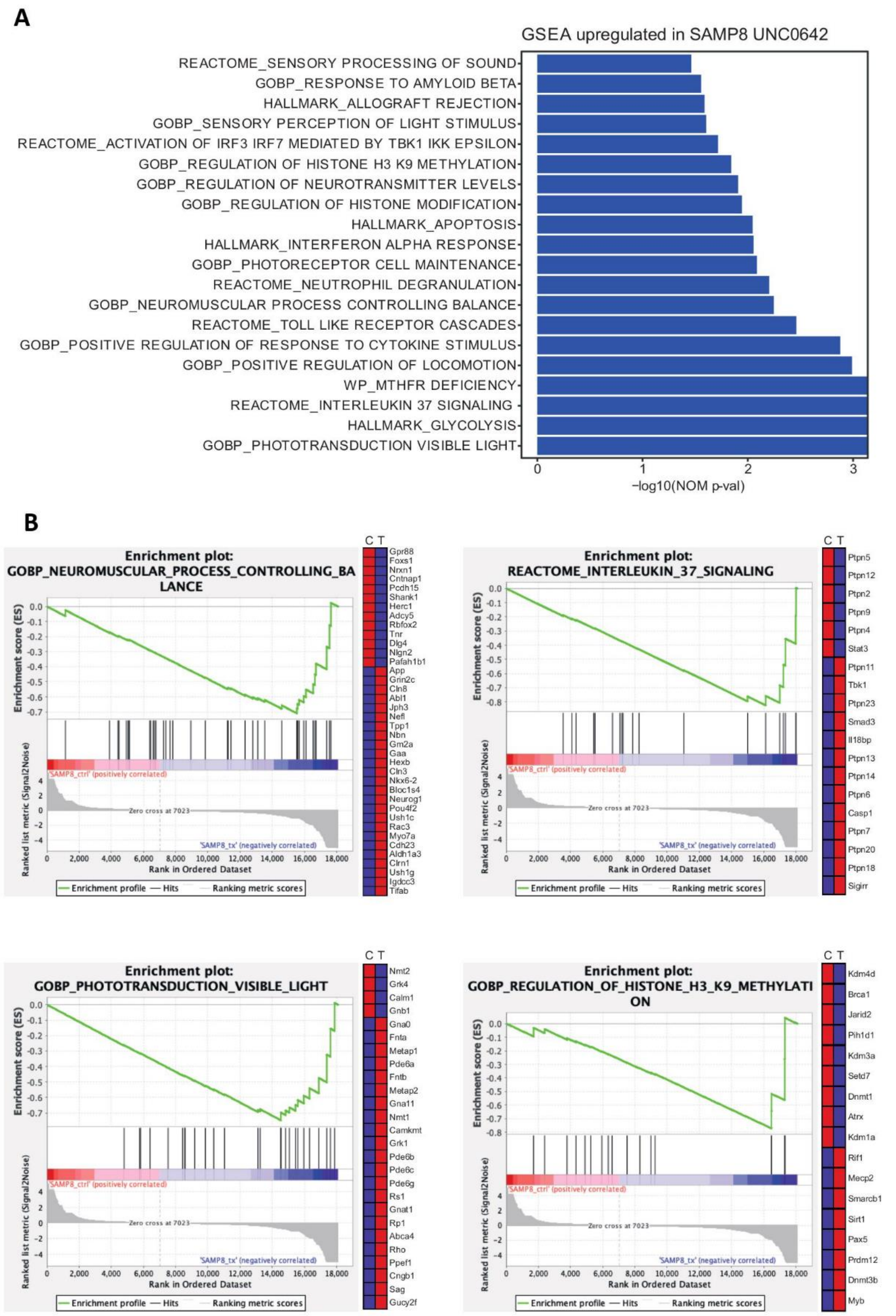

Supplementary Figure 3. (A) Bar graph shows the top GSEA pathways upregulated in SAMP8 UNC0642. NOM *p*-value<0.05 were considered statistically significant. **(B=** GSEA plots of the enriched processes in SAMP8 UNC0642.

© 2023. Bellver-Sanchis A. et al. Published online at http://www.aginganddisease.org/EN/10.14336/AD.2023.0424-2

SUPPLEMENTARY DATA

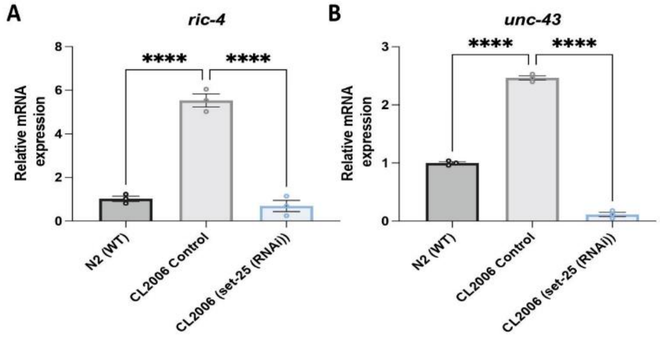

Supplementary Figure 4. (A) Representative gene expression of *ric-4*, **(B)**, and *unc-43*, in *C. elegans*. Gene expression levels were determined by real-time PCR. Values presented are the mean ± SEM; n = 3-5. Each replicate with at least 350 worms in each group from whole petri dish. Groups were compared by One-Way ANOVA and post-hoc Tukey's test; ****p< 0.0001).

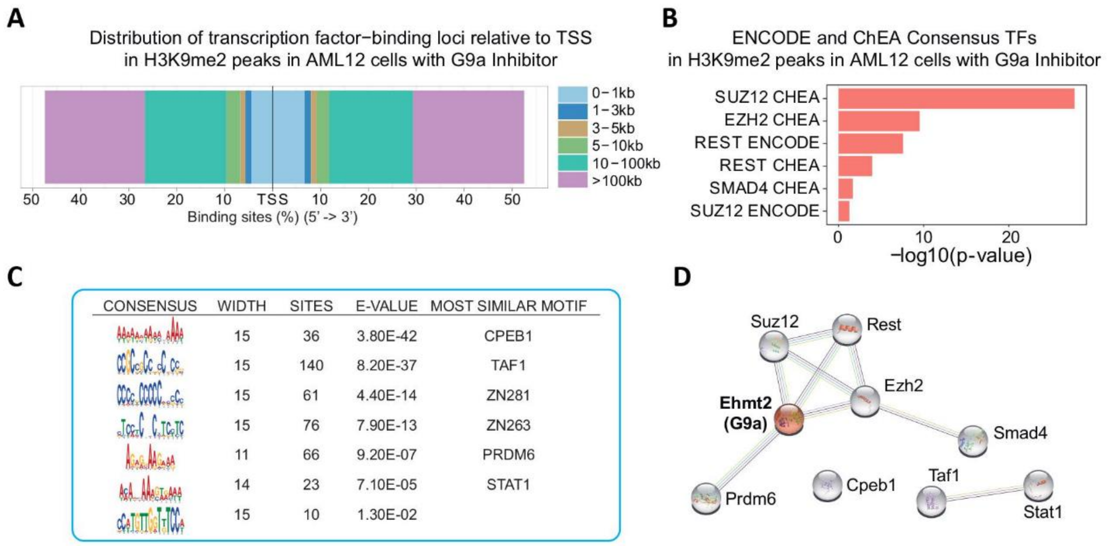

Supplementary Figure 5. (A) Distribution of transcription factor binding relative to TSS in AML12 cells treated with UNC0638 identified within the H3K9me2 peaks. **(B)** Transcription factor binding motifs at H3K9me2 peaks in AML12 cells treated with UNC0638. We considered an adjusted E-value<0.05 as statistically significant. **(C)** ENCODE and ChEA Consensus TFs associated with genes enriched with H3K9me2 at promoters. **(D)** Interaction network of TFs identified in parts (B) and (C).

© 2023. Bellver-Sanchis A. et al. Published online at http://www.aginganddisease.org/EN/10.14336/AD.2023.0424-2

SUPPLEMENTARY DATA

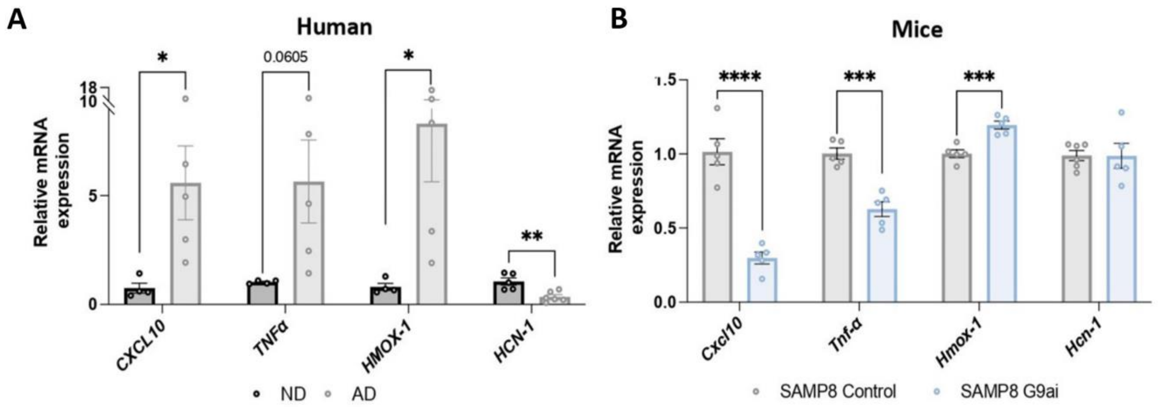

Supplementary Figure 6. (A) Representative gene expression of synaptic plasticity related genes such as CXCL*10*, *TNF-α*, *HMOX-1*, and *HCN-1* in human brain. Values presented are the mean ± SEM; (n = 11 (ND = 5, AD n = 6); Student t-test; *p<0.05, **p<0.01). **(B)** Representative gene expression of synaptic plasticity related genes such as *Cxcl10*, *Tnf-α*, *Hmox-1*, and *Hcn-1* in SAMP8 mice**.** Values presented are the mean ± SEM (n = 12 (SAMP8 Control = 6, SAMP8 G9ai (UNC0642, 5mg/Kg) n = 6); Student's t-test;***p<0.001; ***p<0.001). Data information: Note that experiments in Supplementary Fig. 6B and Fig. 6D were performed at the same time, so the values regarding gene expression of *Cxcl10* and *Tnf-α* are the same but the figures have been split in two for the sake of linearity.

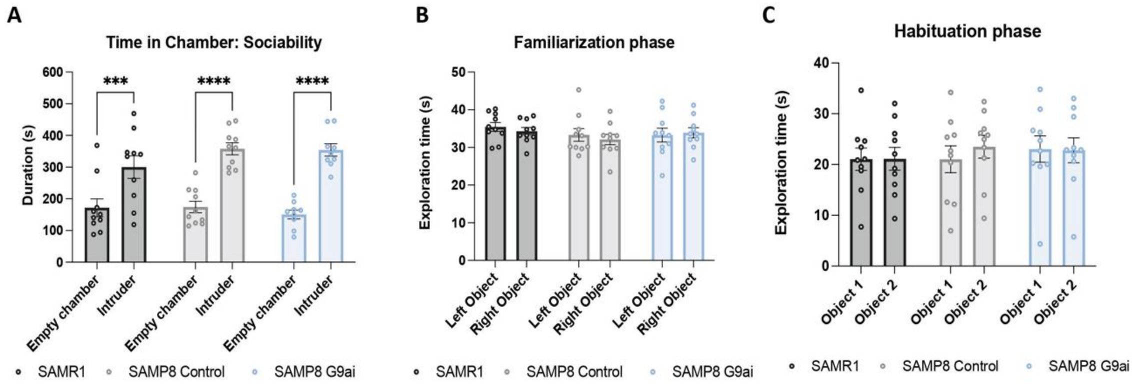

Supplementary Figure 7. For TCT: **(A)** Time spent in chamber. For NORT: **(B)** Familiarization phase. Fort OLT: **(C)** Habituation phase. Values presented are the mean ± SEM; (n = 30 (SAMR1 n = 10, SAMP8 Control n = 10, and SAMP8 G9ai (UNC0642, 5mg/Kg) n = 10); One-Way ANOVA and post-hoc Tukey's test; ***p<0.001; ****p<0.0001).

© 2023. Bellver-Sanchis A. et al. Published online at http://www.aginganddisease.org/EN/10.14336/AD.2023.0424-2

SUPPLEMENTARY DATA

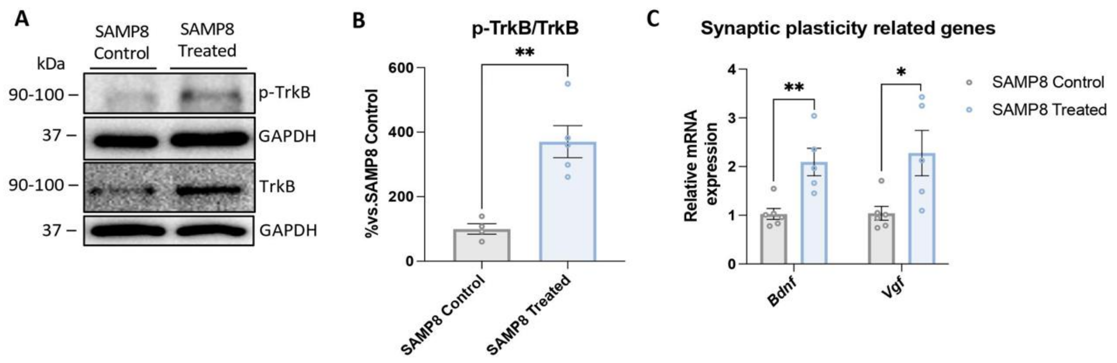

Supplementary Figure 8. (A) and **(B)** Representative WB and quantifications for the ratio of p-TrkB/TrkB. **(C)** Representative gene expression of synaptic plasticity related genes such as *Bdnf*, and *Vgf***.** Values presented are the mean ± SEM; (n = 12 (SAMP8 Control = 6, SAMP8 treated with UNC0642 (5mg/Kg) n = 6)). Groups were compared by Student t-test; **p*<0.05; ***p*<0.01 vs SAMP8 Control.

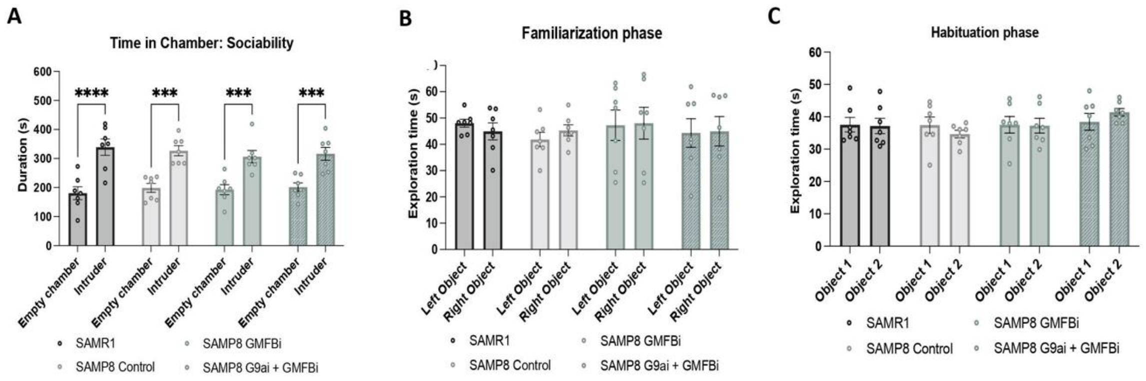

Supplementary Figure 9. For TCT: **(A)** Time spent in chamber. For NORT: **(B)** Familiarization phase. Fort OLT: **(C)** Habituation phase. Values presented are the mean ± SEM; (n = 28-24 (SAMR1 n = 6-7, SAMP8 Control n = 6-7, SAMP8 GMFBi ((1H-Indazol-4-yl)methanol, 12mg/Kg) n= 6-7, and SAMP8 G9ai (UNC0642, 5mg/Kg) + GMFBi ((1H-Indazol-4-yl)methanol, 12mg/Kg) n = 6-7); One-Way ANOVA and post-hoc Tukey's test; ****p*<0.001; *****p*<0.0001.

© 2023. Bellver-Sanchis A. et al. Published online at http://www.aginganddisease.org/EN/10.14336/AD.2023.0424-2

SUPPLEMENTARY DATA

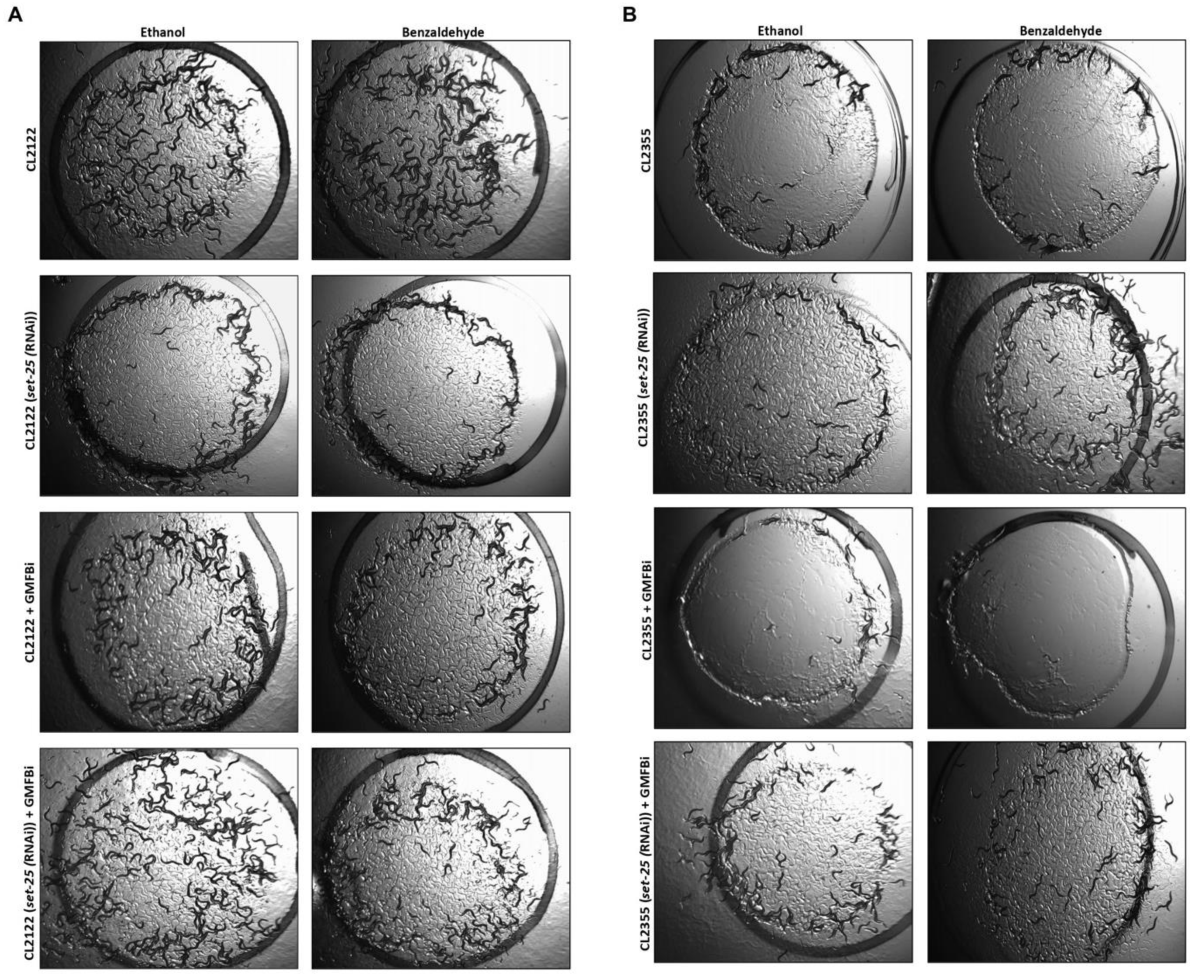

Supplementary Figure 10. Representative image of worms within the attractant (right) or control (left) spot on the chemotaxis assay plates of the different groups **(A)** CL2122 strain, and **(B)** CL2355 strain.

© 2023. Bellver-Sanchis A. et al. Published online at http://www.aginganddisease.org/EN/10.14336/AD.2023.0424-2

SUPPLEMENTARY DATA

Supplementary Table 1. Patients Data.

Gender	Age	Postmortem delay	Diagnosis
Male	85	5 h 45 min	Control
Male	78	2 h 15 min	Control
Male	79	7 h	Control
Male	52	3 h 20 min	Control
Female	72	2 h 45 min	Control
Female	49	5 h	Control
Male	93	7 h 20 min	AD BRAAK STAGE V/C
Male	85	5 h 15 min	AD BRAAK STAGE V/C
Male	74	6 h 30 min	AD BRAAK STAGE V/C
Female	83	3 h 20 min	AD BRAAK STAGE V/C
Female	82	1 h 45 min	AD BRAAK STAGE V/B
Female	72	9 h 30 min	AD BRAAK STAGE V/B
Female	75	4 h 15 min	AD BRAAK STAGE V/B
Female	85	2 h 10 min	AD BRAAK STAGE V/B

Supplementary Table 2. Reagents and kits used in this work.

Reagent, kits, and equipment	Source	Catalog number
Benzaldehyde	Sigma Aldrich	100-52-7
Chemiluminescence-based detection kit	ECL Kit, Millipore	WBKLS0500
EpiQuik Total Histone Extraction HT Kit	EpiGentek	OP-0007-192
Human amyloid-β_{40} ELISA Kit	Invitrogen™	KHB3481
Human amyloid-β_{42} Ultrasensitive ELISA Kit	Invitrogen™	KHB3544
Mouse Aβ_{42} ELISA Kit	Invitrogen™	KMB3441
Mouse Aβ_{40} ELISA Kit	Invitrogen™	KMB3481
TRIsure™	Bioline, Meridian Bioscience	BIO-38032
High Capacity cDNA Reverse Transcription kit	Applied Biosystems™	4368814
Maxima SYBR Green qPCR Master Mix (2X), ROX solution provided	Thermo Scientific™	K0253
mirVana *mi*RNA Isolation kit *with p*henol	Invitrogen™	AM1560
TaqMan miRNA Reverse Transcription kit	Applied Biosystems™	4366596
TaqMan™ Universal PCR Master Mix	Applied Biosystems™	4305719
FD Rapid GolgiStain kit	FD NeuroTechnologies, incs	PK401
Eukitt® Quick-hardening mounting medium	Sigma-Aldrich	25608-33-7
Trypan blue 0,4%	BioRad	1450021
Hoechst	Sigma Aldrich	33258
Thioflavin S	Sigma Aldrich	1326-12-1
Mowiol	Calbiochem	9002-89-5
TC20™ Automated Cell counter	Biorad	1450102

© 2023. Bellver-Sanchis A. et al. Published online at http://www.aginganddisease.org/EN/10.14336/AD.2023.0424-2

SUPPLEMENTARY DATA

Supplementary Table 3. Antibodies used in WB protocol, and in IHC analysis.

Antibody	Host	Source/Catalog	WB dilution
G9a	Rabbit	Abcam/ab40542	1:1000
H3K9me2	Rabbit	Epigentek/A-4035	1:500
Histone H3 total	Rabbit	Cell signaling/#9715	1:1000
GMFB	Mouse	Santa Cruz/ sc-134347	1:500
p38	Mouse	Santa Cruz/sc-81621	1:500
p-p38	Mouse	Santa Cruz/ sc-166182	1:500
CREB	Rabbit	Cell signaling/#4820	1:1000
p-CREB (Ser133)	Rabbit	Cell signaling/#9198	1:1000
BDNF	Rabbit	Bios/BS-4989R	1:1000
NF-κB	Rabbit	Cell signaling/#8242S	1:1000
p- NF-κB	Mouse	Santa Cruz/ sc-136548	1:500
ERK	Rabbit	Cell Signalling/9102	1:1000
p-ERK	Rabbit	Cell Signalling/9101	1:1000
p-TrkB	Mouse	Santa Cruz/sc-8052	1:500
TrkB	Mouse	Santa Cruz/sc-377218	1:500
GAPDH	Mouse	Millipore/MAB374	1:5000
Goat-anti-mouse HRP conjugated	Goat	Biorad/170-5047	1:2000
Goat-anti-rabbit HRP conjugated	Goat	Biorad/170-6515	1:2000
**Antibody**	**Host**	**Source/Catalog**	**ICH dilution**
iNOS	Mouse	Thermofisher/MA5-17139	1:100
Arginase	Mouse	BD Biosciences/ #610708	1:100
Iba1	Mouse	Abcam/Ab107159	1:100
Cy3-conjugated anti-rabbit	Donkey	Jackson ImmunoResearch/711-165-152	1:200
Cy3-conjugated anti-mouse	Donkey	Jackson ImmunoResearch/711-166-150	1:200

G9a: also called euchromatic histone-lysine N-methyltransferase 2 (EHMT2); H3K9me2: Histone 3 Lysine 9 di-methylated; GMFB: Glia maturation factor beta; p38: mitogen-activated protein kinase; CREB: cAMP response element-binding protein; BDNF: Brain Derived Neurotrophic Factor; NF-κB: Nuclear factor kappa-light-chain-enhancer of activated B cell; ERK: extracellular signal-regulated kinases; TrkB: Tropomyosin receptor kinase B; GAPDH: Glyceraldehyde 3-phosphate dehydrogenase; iNOS: Inducible nitric oxide synthase; Iba1: Ionized calcium binding adaptor molecule 1.

Supplementary Table 4. Primers used in qPCR studies.

Target	Forward primer (5'-3')	Reverse primer (3'-5')	Product length
CXCL10	GCTTCCAAGGATGGACCACA	GCAGGGTCAGAACATCCACT	253
HMOX-1	GTGGTTTTTGAGCCATGCGT	CCACTTCTCCTACCGAGCAC	213
TNF-α	GCTGCACTTTGGAGTGATCG	GGGAGAGTGGATGAAGGCTG	170
HCN-1	CACCAGATTGCTGGGTGTCT	ACTGGATTAAAGCGGTGGCA	210
GAPDH	GGGAGCCAAAAGGGTCAT	GAGTCCTTCCACGATACCAA	181
Gmfb	GGAGAGGTGTTGCAGATTGTTTG	GGAAACCATTTGTGGGAAGTGT	150
Snap25	ACGCATTGAGGAAGGGATGG	ATCTGCTCCCGTTCATCCAC	207
Bdnf	GGGAAATCTCCTGAGCCGAG	AGCTTTCTCAACGCCTGTCA	171
Il-6	ATCCAGTTGCCTTCTTGGGACTGA	TAAGCCTCCGACTTGTGAAGTGGT	134
Cxcl10	GGCTAGTCCTAATTGCCCTTGG	TTGTCTCAGGACCATGGCTTG	104
Tnf-α	TCGGGGTGATCGGTCCCCAA	TGGTTTGCTACGACGTGGGCT	139
Hmox-1	TGACACCTGAGGTCAAGCAC	GTCTCTGCAGGGGCAGTATC	184
Hcn-1	ATCTCATCGATCCCGGTGGA	ACGATACGAAGTGCTCTGGC	92
ß-actin	CAACGAGCGGTTCCGAT	GCCACAGGTTCCATACCCA	190
set-25	GACGAAAACGGATGATGGCG	CCGGGCTCGTAGTATGCAAT	288
*crh-1c**	ATGTCAGCGAAAGGTAACGGATC	TCCTCCGGCTCCTTCTTCATC	173
*ikb-1***	GTCCTCGCGCTCTTTTCCG	GCGATCACCAGCAGATGC	120
ric-4	TTCCAGAGGGTCTTGAGGCT	TCGCAACGCTCAAGTTGTTC	163
unc-43	CTACTGCCATCAGCGTGGAA	AACGGTGTTGGTCCTCATCC	170
daf-16	ATTGTGTTCATTTGCCCCGC	GAAGGGAGCCCATCAATGCTC	131
act-1	ATCACCGCTCTTGCCCCATC	GGCCGGACTCGTCGTATTCTTG	151

*Used for ERK phosphorylation assay in cell culture. Cxcl10: C-X-C motif chemokine ligand 10; Hmox-1: Heme Oxygenase-1; Tnf-α: Tumor necrosis factor-alpha; Hcn-1: Hyperpolarization Activated Cyclic Nucleotide Gated Potassium Channel 1; Gapdh: Glyceraldehyde 3-phosphate dehydrogenase; Gmfb: Glia maturation factor beta; Snap25: Synaptosome Associated Protein 25; Bdnf: Brain Derived Neurotrophic Factor; Il-6: Interleukin-6; set-25: SET (trithorax/polycomb) domain containing-25 (ortholog of human G9a); crh-1c: ortholog of human CREB; ikb-1: I Kappa B homolog (ortholog of human NFKB2 (nuclear factor kappa B subunit 2)); ric-4: Resistance to Inhibitors of Cholinesterase (ortholog of human SNAP25 (synaptosome associated protein 25)) ; unc-43: UNCoordinated (ortholog of human CAMK2D (calcium/calmodulin dependent protein kinase II delta)) ; daf-16: abnormal DAuer Formation (ortholog of human FOXO4 (forkhead box O4)); act-1: actin (ortholog of human ACTIN beta)

Supplementary Table 5. Plasmids to generate GMFB methylation site mutants.

Plasmid	Sequence
K20R-KD	CGCCATTCTGCCTGGGGACGTCGGAGCAAGCTTGATTTAGGTGACACTATAGAATACAAGCTACTTGTTCTTTTTGCAGGATCCATGTACCCATACGATGTTCCAGATTACGCTTATCCTTATGACGTACCTGACTATGCATACCCTTATGATGTACCAGACTACGCTGGCCGGCCAGAATTCATGAGTGAGTCTTTGGTTGTTTGTGATGTTGCCGAAGATTTAGTGGAAAAGCTGAGACGGTTTCGTTTTCGCAAAGAAACGAACAACGCTGCTATTATAATGAAGATTGACAAGGATAAACGCCTGGTGGTACTGGATGAGGAGCTTGAGGGCATTTCACCAGATGAACTTAAAGATGAACTACCTGAACGACAACCTCGCTTCATTGTGTATAGTTATAAATATCAACATGATGATGGAAGAGTTTCATATCCTCTGTGCTTTATTTTCTCCAGTCCTGTTGGATGTAAGCCTGAACAACAGATGATGTATGCTGGAAGTAAGAATAAGCTAGTCCAGACAGCTGAACTAACCAAGGTATTTGAAATAAGAAATACCGAAGACCTAACTGAAGAATGGTTACGTGAGAAACTTGGATTTTTTCACTAGTCTAGAACTATAGTGAGTCGTATTACGTAGATCCAGACATGATAAGATACATTGATGAGTTTGGACAAACCACAACTAGAATGCAGTGAAAAAAATGCTTTATTTGTGAAATTTGTGATGCTATTGCTTTATTTGTAACCATTATAAGCTGCAATAAACAAGTTAACAACAACAATTGCATTCATTTTATGTTTCAGGTTCAGGGGGAGGTGTGGGAGGTTTTTTAATTCGCGGCCGCGGCGCCAATGCATTGGGCCCGGTACCCAGCTTTTGTTCCCTTTAGTGAGGGTTAATTGCGCGCTTGGCGTAATCATGGTCATAGCTGTTTCCTGTGTGAAATTGTTATCCGCTCACAATTCCACACAACATACGAGCCGGAAGCATAAAGTGTAAAGCCTGGGGTGCCTAATGAGTGAGCTAACTCACATTAATTGCGTTGCGCTCACTGCCCGCTTTCCAGTCGGGAAACCTGTCGTGCCAGCTGCATTAATGAATCGGCCAACGCGCGGGGAGAGGCGGTTTGCGTATTGGGCGCTCTTCCGCTTCCTCGCTCACTGACTCGCTGCGCTCGGTCGTTCGGCTGCGGCGAGCGGTATCAGCTCACTCAAAGGCGGTAATACGGTTATCCACAGAATCAGGGGATAACGCAGGAAAGAACATGTGAGCAAAAGGCCAGCAAAAGGCCAGGAACCGTAAAAAGGCCGCGTTGCTGGCGTTTTTCCATAGGCTCCGCCCCCCTGACGAGCATCACAAAAATCGACGCTCAAGTCAGAGGTGGCGAAACCCGACAGGACTATAAAGATACCAGGCGTTTCCCCCTGGAAGCTCCCTCGTGCGCTCTCCTGTTCCGACCCTGCCGCTTACCGGATACCTGTCCGCCTTTCTCCCTTCGGGAAGCGTGGCGCTTTCTCATAGCTCACGCTGTAGGTATCTCAGTTCGGTGTAGGTCGTTCGCTCCAAGCTGGGCTGTGTGCACGAACCCCCCGTTCAGCCCGACCGCTGCGCCTTATCCGGTAACTATCGTCTTGAGTCCAACCCGGTAAGACACGACTTATCGCCACTGGCAGCAGCCACTGGTAACAGGATTAGCAGAGCGAGGTATGTAGGCGGTGCTACAGAGTTCTTGAAGTGGTGGCCTAACTACGGCTACACTAGAAGGACAGTATTTGGTATCTGCGCTCTGCTGAAGCCAGTTACCTTCGGAAAAAGAGTTGGTAGCTCTTGATCCGGCAAACAAACCACCGCTGGTAGCGGTGGTTTTTTTGTTTGCAAGCAGCAGATTACGCGCAGAAAAAAAGGATCTCAAGAAGATCCTTTGATCTTTTCTACGGGGTCTGACGCTCAGTGGAACGAAAACTCACGTTAAGGGATTTTGGTCATGAGATTATCAAAAAGGATCTTCACCTAGATCCTTTTAAATTAAAAATGAAGTTTTAAATCAATCTAAAGTATATATGAGTAAACTTGGTCTGACAGTTACCAATGCTTAATCAGTGAGGCACCTATCTCAGCGATCTGTCTATTTCGTTCATCCATAGTTGCCTGACTCCCCGTCGTGTAGATAACTACGATACGGGAGGGCTTACCATCTGGCCCCAGTGCTGCAATGATACCGCGAGACCCACGCTCACCGGCTCCAGATTTATCAGCAATAAACCAGCCAGCCGGAAGGGCCGAGCGCAGAAGTGGTCCTGCAACTTTATCCGCCTCCATCCAGTCTATTAATTGTTGCCGGGAAGCTAGAGTAAGTAGTTCGCCAGTTAATAGTTTGCGCAACGTTGTTGCCATTGCTACAGGCATCGTGGTGTCACGCTCGTCGTTTGGTATGGCTTCATTCAGCTCCGGTTCCCAACGATCAAGGCGAGTTACATGATCCCCCATGTTGTGCAAAAAAGCGGTTAGCTCCTTCGGTCCTCCGATCGTTGTCAGAAGTAAGTTGGCCGCAGTGTTATCACTCATGGTTATGGCAGCACTGCATAATTCTCTTACTGTCATGCCATCCGTAAGATGCTTTTCTGTGACTGGTGAGTACTCAACCAAGTCATTCTGAGAATAGTGTATGCGGCGACCGAGTTGCTCTTGCCCGGCGTCAATACGGGATAATACCGCGCCACATAGCAGAACTTTAAAAGTGCTCATCATTGGAAAACGTTCTTCGGGGCGAAAACTCTCAAGGATCTTACCGCTGTTGAGATCCAGTTCGATGTAACCCACTCGTGCACCCAACTGATCTTCAGCATCTTTTACTTTCACCAGCGTTTCTGGGTGAGCAAAAACAGGAAGGCAAAATGCCGCAAAAAAGGGAATAAGGGCGACACGGAAATGTTGAATACTCATACTCTTCCTTTTTCAATATTATTGAAGCATTTATCAGGGTTATTGTCTCATGAGCGGATACATATTTGAATGTATTTAGAAAAATAAACAAATAGGGGTTCCGCGCACATTTCCCCGAAAAGTGCCACCTAAATTGTAAGCGTTAATATTTTGTTAAAATTCGCGTTAAATTTTTGTTAAATCAGCTCATTTTTTAACCAATAGGCCGAAATCGGCAAAATCCCTTATAAATCAAAAGAATAGACCGAGATAGGGTTGAGTGTTGTTCCAGTTTGGAACAAGAGTCCACTATTAAAGAACGTGGACTCCAACGTCAAAGGGCGAAAAACCGTCTATCAGGGCGATGGCCCACTACGTGAACCATCACCCTAATCAAGTTTTTTGGGGTCGAGGTGCCGTAAAGCACTAAATCGGAACCCTAAAGGGAGCCCCCGATTTAGAGCTTGACGGGGAAAGCCGGCGAACGTGGCGAGAAAGGAAGGGAAGAAAGCGAAAGGAGCGGGCGCTAGGGCGCTGGCAAGTGTAGCGGTCACGCTGCGCGTAACCACCACACCCGCCGCGCTTAATGCGCCGCTACAGGGCGCGTCCCATTCGCCATTCAGGCTGCGCAACTGTTGGGAAGGGCGATCGGTGCGGGCCTCTTCGCTATTACGCCAGTCGACCATAGCCAATTCAATATGGCGTATATGGACTCATGCCAATTCAATATGGTGGATCTGGACCTGTGCCAATTCAATATGGCGTATATGGACTCGTGCCAATTCAATATGGTGGATCTGGACCCCAGCCAATTCAATATGGCGGACTTGGCACCATGCCAATTCAATATGGCGGACTTGGCACTGTGCCAACTGGGGAGGGGTCTACTTGGCACGGTGCCAAGTTTGAGGAGGGGTCTTGGCCCTGTGCCAAGTCCGCCATATTGAATTGGCATGGTGCCAATAATGGCGGCCATATTGGCTATATGCCAGGATCAATATATAGGCAATATCCAATATGGCCCTATGCCAATATGGCTATTGGCCAGGTTCAATACTATGTATTGGCCCTATGCCATATAGTATTCCATATATGGGTTTTCCTATTGACGTAGATAGCCCCTCCCAATGGGCGGTCCCATATACCATATATGGGGCTTCCTAATACCGCCCATAGCCACTCCCCCATTGACGTCAATGGTCTCTATATATGGTCTTTCCTATTGACGTCATATGGGCGGTCCTATTGACGTATATGGCGCCTCCCCCATTGACGTCAATTACGGTAAATGGCCCGCCTGGCTCAATGCCCATTGACGTCAATAGGACCACCCACCATTGACGTCAATGGGATGGCTCATTGCCCATTCATATCCGTTCTCACGCCCCCTATTGACGTCAATGACGGTAAATGGCCCACTTGGCAGTACATCAATATCTATTAATAGTAACTTGGCAAGTACATTACTATTGGAAGGACGCCAGGGTACATTGGCAGTACTCCCATTGACGTCAATGGCGGTAAATGGCCCGCGATGGCTGCCAAGTACATCCCCATTGACGTCAATGGGGAGGGGCAATGACGCAAATGGGCGTTCCATTGACGTAAATGGGCGGTAGGCGTGCCTAATGGGAGGTCTATATAAGCAATGCTCGTTTAGGGAAC
K25R-KD	CGCCATTCTGCCTGGGGACGTCGGAGCAAGCTTGATTTAGGTGACACTATAGAATACAAGCTACTTGTTCTTTTTGCAGGATCCATGTACCCATACGATGTTCCAGATTACGCTTATCCTTATGACGTACCTGACTATGCATACCCTTATGATGTACCAGACTACGCTGGCCGGCCAGAATTCATGAGTGAGTCTTTGGTTGTTTGTGATGTTGCCGAAGATTTAGTGGAAAAGCTGAGAAAGTTTCGTTTTCGCCGAGAAACGAACAACGCTGCTATTATAATGAAGATTGACAAGGATAAACGCCTGGTGGTACTGGATGAGGAGCTTGAGGGCATTTCACCAGATGAACTTAAAGATGAACTACCTGAACGACAACCTCGCTTCATTGTGTATAGTTATAAATATCAACATGATGATGGAAGAGTTTCATATCCTCTGTGCTTTATTTTCTCCAGTCCTGTTGGATGTAAGCCTGAACAACAGATGATGTATGCTGGAAGTAAGAATAAGCTAGTCCAGACAGCTGAACTAACCAAGGTATTTGAAATAAGAAATACCGAAGACCTAACTGAAGAATGGTTACGTGAGAAACTTGGATTTTTTCACTAGTCTAGAACTATAGTGAGTCGTATTACGTAGATCCAGACATGATAAGATACATTGATGAGTTTGGACAAACCACAACTAGAATGCAGTGAAAAAAATGCTTTATTTGTGAAATTTGTGATGCTATTGCTTTATTTGTAACCATTATAAGCTGCAATAAACAAGTTAACAACAACAATTGCATTCATTTTATGTTTCAGGTTCAGGGGGAGGTGTGGGAGGTTTTTTAATTCGCGGCCGCGGCGCCAATGCATTGGGCCCGGTACCCAGCTTTTGTTCCCTTTAGTGAGGGTTAATTGCGCGCTTGGCGTAATCATGGTCATAGCTGTTTCCTGTGTGAAATTGTTATCCGCTCACAATTCCACACAACATACGAGCCGGAAGCATAAAGTGTAAAGCCTGGGGTGCCTAATGAGTGAGCTAACTCACATTAATTGCGTTGCGCTCACTGCCCGCTTTCCAGTCGGGAAACCTGTCGTGCCAGCTGCATTAATGAATCGGCCAACGCGCGGGGAGAGGCGGTTTGCGTATTGGGCGCTCTTCCGCTTCCTCGCTCACTGACTCGCTGCGCTCGGTCGTTCGGCTGCGGCGAGCGGTATCAGCTCACTCAAAGGCGGTAATACGGTTATCCACAGAATCAGGGGATAACGCAGGAAAGAACATGTGAGCAAAAGGCCAGCAAAAGGCCAGGAACCGTAAAAAGGCCGCGTTGCTGGCGTTTTTCCATAGGCTCCGCCCCCCTGACGAGCATCACAAAAATCGACGCTCAAGTCAGAGGTGGCGAAACCCGACAGGACTAT

SUPPLEMENTARY DATA

AAAGATACCAGGCGTTTCCCCCTGGAAGCTCCCTCGTGCGCTCTCCTGTTCCGACCCTGCCGCTTACCGGATACCTGTCCGCCTTTCTCCCTTCGGGAAGCGTGGCGCTTTCTCATAGCTCACGCTGTAGGTATCTCAGTTCG
GTGTAGGTCGTTCGCTCCAAGCTGGGCTGTGTGCACGAACCCCCCGTTCAGCCCGACCGCTGCGCCTTATCCGGTAACTATCGTCTTGAGTCCAACCCGGTAAGACACGACTTATCGCCACTGGCAGCAGCCACTGGTAAC
AGGATTAGCAGAGCGAGGTATGTAGGCGGTGCTACAGAGTTCTTGAAGTGGTGGCCTAACTACGGCTACACTAGAAGGACAGTATTTGGTATCTGCGCTCTGCTGAAGCCAGTTACCTTCGGAAAAAGAGTTGGTAGCTC
TTGATCCGGCAAACAAACCACCGCTGGTAGCGGTGGTTTTTTTGTTTGCAAGCAGCAGATTACGCGCAGAAAAAAAGGATCTCAAGAAGATCCTTTGATCTTTTCTACGGGGTCTGACGCTCAGTGGAACGAAAACTCACG
TTAAGGGATTTTGGTCATGAGATTATCAAAAAGGATCTTCACCTAGATCCTTTTAAATTAAAAATGAAGTTTTAAATCAATCTAAAGTATATATGAGTAAACTTGGTCTGACAGTTACCAATGCTTAATCAGTGAGGCACCT
ATCTCAGCGATCTGTCTATTTCGTTCATCCATAGTTGCCTGACTCCCCGTCGTGTAGATAACTACGATACGGGAGGGCTTACCATCTGGCCCCAGTGCTGCAATGATACCGCGAGACCCACGCTCACCGGCTCCAGATTTAT
CAGCAATAAACCAGCCAGCCGGAAGGGCCGAGCGCAGAAGTGGTCCTGCAACTTTATCCGCCTCCATCCAGTCTATTAATTGTTGCCGGGAAGCTAGAGTAAGTAGTTCGCCAGTTAATAGTTTGCGCAACGTTGTTGCCA
TTGCTACAGGCATCGTGGTGTCACGCTCGTCGTTTGGTATGGCTTCATTCAGCTCCGGTTCCCAACGATCAAGGCGAGTTACATGATCCCCCATGTTGTGCAAAAAAGCGGTTAGCTCCTTCGGTCCTCCGATCGTTGTCAG
AAGTAAGTTGGCCGCAGTGTTATCACTCATGGTTATGGCAGCACTGCATAATTCTCTTACTGTCATGCCATCCGTAAGATGCTTTTCTGTGACTGGTGAGTACTCAACCAAGTCATTCTGAGAATAGTGTATGCGGCGACCG
AGTTGCTCTTGCCCGGCGTCAATACGGGATAATACCGCGCCACATAGCAGAACTTTAAAAGTGCTCATCATTGGAAAACGTTCTTCGGGGCGAAAACTCTCAAGGATCTTACCGCTGTTGAGATCCAGTTCGATGTAACCC
ACTCGTGCACCCAACTGATCTTCAGCATCTTTTACTTTCACCAGCGTTTCTGGGTGAGCAAAAACAGGAAGGCAAAATGCCGCAAAAAAGGGAATAAGGGCGACACGGAAATGTTGAATACTCATACTCTTCCTTTTTCAA
TATTATTGAAGCATTTATCAGGGTTATTGTCTCATGAGCGGATACATATTTGAATGTATTTAGAAAAATAAACAAATAGGGGTTCCGCGCACATTTCCCCGAAAAGTGCCACCTAAATTGTAAGCGTTAATATTTTGTTAAA
ATTCGCGTTAAATTTTTGTTAAATCAGCTCATTTTTTAACCAATAGGCCGAAATCGGCAAAATCCCTTATAAATCAAAAGAATAGACCGAGATAGGGTTGAGTGTTGTTCCAGTTTGGAACAAGAGTCCACTATTAAAGAA
CGTGGACTCCAACGTCAAAGGGCGAAAAACCGTCTATCAGGGCGATGGCCCACTACGTGAACCATCACCCTAATCAAGTTTTTTGGGGTCGAGGTGCCGTAAAGCACTAAATCGGAACCCTAAAGGGAGCCCCCGATTTA
GAGCTTGACGGGGAAAGCCGGCGAACGTGGCGAGAAAGGAAGGGAAGAAAGCGAAAGGAGCGGGCGCTAGGGCGCTGGCAAGTGTAGCGGTCACGCTGCGCGTAACCACCACACCCGCCGCGCTTAATGCGCCGCTAC
AGGGCGCGTCCCATTCGCCATTCAGGCTGCGCAACTGTTGGGAAGGGCGATCGGTGCGGGCCTCTTCGCTATTACGCCAGTCGACCATAGCCAATTCAATATGGCGTATATGGACTCATGCCAATTCAATATGGTGGATCT
GGACCTGTGCCAATTCAATATGGCGTATATGGACTCGTGCCAATTCAATATGGTGGATCTGGACCCCAGCCAATTCAATATGGCGGACTTGGCACCATGCCAATTCAATATGGCGGACTTGGCACTGTGCCAACTGGGGAG
GGGTCTACTTGGCACGGTGCCAAGTTTGAGGAGGGGTCTTGGCCCTGTGCCAAGTCCGCCATATTGAATTGGCATGGTGCCAATAATGGCGGCCATATTGGCTATATGCCAGGATCAATATATAGGCAATATCCAATATGG
CCCTATGCCAATATGGCTATTGGCCAGGTTCAATACTATGTATTGGCCCTATGCCATATAGTATTCCATATATGGGTTTTCCTATTGACGTAGATAGCCCCTCCCAATGGGCGGTCCCATATACCATATATGGGGCTTCCTAA
TACCGCCCATAGCCACTCCCCCATTGACGTCAATGGTCTCTATATATGGTCTTTCCTATTGACGTCATATGGGCGGTCCTATTGACGTATATGGCGCCTCCCCCATTGACGTCAATTACGGTAAATGGCCCGCCTGGCTCAAT
GCCCATTGACGTCAATAGGACCACCCACCATTGACGTCAATGGGATGGCTCATTGCCCATTCATATCCGTTCTCACGCCCCCTATTGACGTCAATGACGGTAAATGGCCCACTTGGCAGTACATCAATATCTATTAATAGTA
ACTTGGCAAGTACATTACTATTGGAAGGACGCCAGGGTACATTGGCAGTACTCCCATTGACGTCAATGGCGGTAAATGGCCCGCGATGGCTGCCAAGTACATCCCCATTGACGTCAATGGGGAGGGGCAATGACGCAAAT
GGGCGTTCCATTGACGTAAATGGGCGGTAGGCGTGCCTAATGGGAGGTCTATATAAGCAATGCTCGTTTAGGGAAC
K20RK2 CGCCATTCTGCCTGGGGACGTCGGAGCAAGCTTGATTTAGGTGACACTATAGAATACAAGCTACTTGTTCTTTTTGCAGGATCCATGTACCCATACGATGTTCCAGATTACGCTTATCCTTATGACGTACCTGACTATGCAT
5R-KD ACCCTTATGATGTACCAGACTACGCTGGCCGGCCAGAATTCATGAGTGAGTCTTTGGTTGTTTGTGATGTTGCCGAAGATTTAGTGGAAAAGCTGAGACGGTTTCGTTTTCGCCGAGAAACGAACAACGCTGCTATTATAA
TGAAGATTGACAAGGATAAACGCCTGGTGGTACTGGATGAGGAGCTTGAGGGCATTTCACCAGATGAACTTAAAGATGAACTACCTGAACGACAACCTCGCTTCATTGTGTATAGTTATAAATATCAACATGATGATGGA
AGAGTTTCATATCCTCTGTGCTTTATTTTCTCCAGTCCTGTTGGATGTAAGCCTGAACAACAGATGATGTATGCTGGAAGTAAGAATAAGCTAGTCCAGACAGCTGAACTAACCAAGGTATTTGAAATAAGAAATACCGAA
GACCTAACTGAAGAATGGTTACGTGAGAAACTTGGATTTTTTCACTAGTCTAGAACTATAGTGAGTCGTATTACGTAGATCCAGACATGATAAGATACATTGATGAGTTTGGACAAACCACAACTAGAATGCAGTGAAAA
AAATGCTTTATTTGTGAAATTTGTGATGCTATTGCTTTATTTGTAACCATTATAAGCTGCAATAAACAAGTTAACAACAACAATTGCATTCATTTTATGTTTCAGGTTCAGGGGGAGGTGTGGGAGGTTTTTTAATTCGCGGC
CGCGGCGCCAATGCATTGGGCCCGGTACCCAGCTTTTGTTCCCTTTAGTGAGGGTTAATTGCGCGCTTGGCGTAATCATGGTCATAGCTGTTTCCTGTGTGAAATTGTTATCCGCTCACAATTCCACACAACATACGAGCCG
GAAGCATAAAGTGTAAAGCCTGGGGTGCCTAATGAGTGAGCTAACTCACATTAATTGCGTTGCGCTCACTGCCCGCTTTCCAGTCGGGAAACCTGTCGTGCCAGCTGCATTAATGAATCGGCCAACGCGCGGGGAGAGGC
GGTTTGCGTATTGGGCGCTCTTCCGCTTCCTCGCTCACTGACTCGCTGCGCTCGGTCGTTCGGCTGCGGCGAGCGGTATCAGCTCACTCAAAGGCGGTAATACGGTTATCCACAGAATCAGGGGATAACGCAGGAAAGAAC
ATGTGAGCAAAAGGCCAGCAAAAGGCCAGGAACCGTAAAAAGGCCGCGTTGCTGGCGTTTTTCCATAGGCTCCGCCCCCCTGACGAGCATCACAAAAATCGACGCTCAAGTCAGAGGTGGCGAAACCCGACAGGACTAT
AAAGATACCAGGCGTTTCCCCCTGGAAGCTCCCTCGTGCGCTCTCCTGTTCCGACCCTGCCGCTTACCGGATACCTGTCCGCCTTTCTCCCTTCGGGAAGCGTGGCGCTTTCTCATAGCTCACGCTGTAGGTATCTCAGTTCG
GTGTAGGTCGTTCGCTCCAAGCTGGGCTGTGTGCACGAACCCCCCGTTCAGCCCGACCGCTGCGCCTTATCCGGTAACTATCGTCTTGAGTCCAACCCGGTAAGACACGACTTATCGCCACTGGCAGCAGCCACTGGTAAC
AGGATTAGCAGAGCGAGGTATGTAGGCGGTGCTACAGAGTTCTTGAAGTGGTGGCCTAACTACGGCTACACTAGAAGGACAGTATTTGGTATCTGCGCTCTGCTGAAGCCAGTTACCTTCGGAAAAAGAGTTGGTAGCTC
TTGATCCGGCAAACAAACCACCGCTGGTAGCGGTGGTTTTTTTGTTTGCAAGCAGCAGATTACGCGCAGAAAAAAAGGATCTCAAGAAGATCCTTTGATCTTTTCTACGGGGTCTGACGCTCAGTGGAACGAAAACTCACG
TTAAGGGATTTTGGTCATGAGATTATCAAAAAGGATCTTCACCTAGATCCTTTTAAATTAAAAATGAAGTTTTAAATCAATCTAAAGTATATATGAGTAAACTTGGTCTGACAGTTACCAATGCTTAATCAGTGAGGCACCT
ATCTCAGCGATCTGTCTATTTCGTTCATCCATAGTTGCCTGACTCCCCGTCGTGTAGATAACTACGATACGGGAGGGCTTACCATCTGGCCCCAGTGCTGCAATGATACCGCGAGACCCACGCTCACCGGCTCCAGATTTAT
CAGCAATAAACCAGCCAGCCGGAAGGGCCGAGCGCAGAAGTGGTCCTGCAACTTTATCCGCCTCCATCCAGTCTATTAATTGTTGCCGGGAAGCTAGAGTAAGTAGTTCGCCAGTTAATAGTTTGCGCAACGTTGTTGCCA
TTGCTACAGGCATCGTGGTGTCACGCTCGTCGTTTGGTATGGCTTCATTCAGCTCCGGTTCCCAACGATCAAGGCGAGTTACATGATCCCCCATGTTGTGCAAAAAAGCGGTTAGCTCCTTCGGTCCTCCGATCGTTGTCAG
AAGTAAGTTGGCCGCAGTGTTATCACTCATGGTTATGGCAGCACTGCATAATTCTCTTACTGTCATGCCATCCGTAAGATGCTTTTCTGTGACTGGTGAGTACTCAACCAAGTCATTCTGAGAATAGTGTATGCGGCGACCG
AGTTGCTCTTGCCCGGCGTCAATACGGGATAATACCGCGCCACATAGCAGAACTTTAAAAGTGCTCATCATTGGAAAACGTTCTTCGGGGCGAAAACTCTCAAGGATCTTACCGCTGTTGAGATCCAGTTCGATGTAACCC
ACTCGTGCACCCAACTGATCTTCAGCATCTTTTACTTTCACCAGCGTTTCTGGGTGAGCAAAAACAGGAAGGCAAAATGCCGCAAAAAAGGGAATAAGGGCGACACGGAAATGTTGAATACTCATACTCTTCCTTTTTCAA
TATTATTGAAGCATTTATCAGGGTTATTGTCTCATGAGCGGATACATATTTGAATGTATTTAGAAAAATAAACAAATAGGGGTTCCGCGCACATTTCCCCGAAAAGTGCCACCTAAATTGTAAGCGTTAATATTTTGTTAAA
ATTCGCGTTAAATTTTTGTTAAATCAGCTCATTTTTTAACCAATAGGCCGAAATCGGCAAAATCCCTTATAAATCAAAAGAATAGACCGAGATAGGGTTGAGTGTTGTTCCAGTTTGGAACAAGAGTCCACTATTAAAGAA
CGTGGACTCCAACGTCAAAGGGCGAAAAACCGTCTATCAGGGCGATGGCCCACTACGTGAACCATCACCCTAATCAAGTTTTTTGGGGTCGAGGTGCCGTAAAGCACTAAATCGGAACCCTAAAGGGAGCCCCCGATTTA
GAGCTTGACGGGGAAAGCCGGCGAACGTGGCGAGAAAGGAAGGGAAGAAAGCGAAAGGAGCGGGCGCTAGGGCGCTGGCAAGTGTAGCGGTCACGCTGCGCGTAACCACCACACCCGCCGCGCTTAATGCGCCGCTAC
AGGGCGCGTCCCATTCGCCATTCAGGCTGCGCAACTGTTGGGAAGGGCGATCGGTGCGGGCCTCTTCGCTATTACGCCAGTCGACCATAGCCAATTCAATATGGCGTATATGGACTCATGCCAATTCAATATGGTGGATCT
GGACCTGTGCCAATTCAATATGGCGTATATGGACTCGTGCCAATTCAATATGGTGGATCTGGACCCCAGCCAATTCAATATGGCGGACTTGGCACCATGCCAATTCAATATGGCGGACTTGGCACTGTGCCAACTGGGGAG
GGGTCTACTTGGCACGGTGCCAAGTTTGAGGAGGGGTCTTGGCCCTGTGCCAAGTCCGCCATATTGAATTGGCATGGTGCCAATAATGGCGGCCATATTGGCTATATGCCAGGATCAATATATAGGCAATATCCAATATGG
CCCTATGCCAATATGGCTATTGGCCAGGTTCAATACTATGTATTGGCCCTATGCCATATAGTATTCCATATATGGGTTTTCCTATTGACGTAGATAGCCCCTCCCAATGGGCGGTCCCATATACCATATATGGGGCTTCCTAA
TACCGCCCATAGCCACTCCCCCATTGACGTCAATGGTCTCTATATATGGTCTTTCCTATTGACGTCATATGGGCGGTCCTATTGACGTATATGGCGCCTCCCCCATTGACGTCAATTACGGTAAATGGCCCGCCTGGCTCAAT
GCCCATTGACGTCAATAGGACCACCCACCATTGACGTCAATGGGATGGCTCATTGCCCATTCATATCCGTTCTCACGCCCCCTATTGACGTCAATGACGGTAAATGGCCCACTTGGCAGTACATCAATATCTATTAATAGTA
ACTTGGCAAGTACATTACTATTGGAAGGACGCCAGGGTACATTGGCAGTACTCCCATTGACGTCAATGGCGGTAAATGGCCCGCGATGGCTGCCAAGTACATCCCCATTGACGTCAATGGGGAGGGGCAATGACGCAAAT
GGGCGTTCCATTGACGTAAATGGGCGGTAGGCGTGCCTAATGGGAGGTCTATATAAGCAATGCTCGTTTAGGGAAC

SUPPLEMENTARY DATA

Supplementary Table 6. Reagents and antibodies used in Flag-tag assay.

Reagent, and antibodies	Source	Catalog number
Flag	Sigma Aldrich	F1804
GMFB	Proteintech	10690-1-AP
pan-me1/2 lysine	PTM Biolabs Inc	PTM-602
HA (0906-1)	HuaBio	0906-1
Hemagglutinin (HA) affinity gel (B23301)	BioTools	B23301
Secondary horseradish peroxidase–conjugated mouse or rabbit	Jackson ImmunoResearch Laboratories	
BIX-01294	Selleck	S8006

Supplementary Table 7. RNA-seq transcripts modified in SAMP8 after UNC0642 treatment.

Gene	logFC	PValue
Ipo7	-11,319704	1,97E-02
Krt80	-8,247241566	5,19E-06
Pla2g4b	-7,866249201	2,83E-05
H2-T9	-7,618812145	9,64E-05
Gm7367	-7,345523261	2,91E-04
Gm3488	-7,26862838	2,91E-04
Xntrpc	-6,880387877	2,00E-03
Nxf7	-6,826410991	2,00E-03
Rxfp2	-6,826410991	2,00E-03
Gm14151	-6,809816111	2,00E-03
Hist2h3c2	-6,739123908	2,86E-03
Tnfsf12Tnfsf13	-6,562838958	4,17E-03
Gm21541	-6,552236283	6,15E-03
Olfr112	-6,45170799	6,15E-03
Gm5803	-6,325081966	9,22E-03
Dynlt1f	-6,323524076	9,22E-03
Hist1h2ai	-6,287220269	1,41E-02
Ppp1r1c	-6,133894589	1,41E-02
Gm13304	-6,083240369	2,19E-02
Gm10409	-6,023084641	2,19E-02
Gm9833	-5,993999277	2,19E-02
Ccl21c	-5,974278078	2,19E-02
1700027H10Rik	-5,944181409	3,48E-02

SUPPLEMENTARY DATA

Allc	-5,944181409	3,48E-02
B230312C02Rik	-5,944181409	3,48E-02
D6Ertd474e	-5,944181409	3,48E-02
Gm4477	-5,852093062	3,48E-02
Gm20594	-5,76820844	4,97E-11
Gm5424	-5,462817922	2,54E-07
Lars2	-5,313590279	1,83E-11
Muc19	-4,215351762	2,30E-03
Myl4	-3,990040848	2,79E-06
Xirp2	-3,889491181	2,95E-06
Hfe2	-3,749401968	1,02E-02
Igfn1	-3,606724192	2,55E-06
Nlrp5-ps	-3,524547518	3,57E-03
Gm5643	-3,517064631	2,13E-05
Gk2	-3,374698968	2,80E-02
Olfr78	-3,331010331	2,09E-03
Mypn	-3,288097436	2,95E-04
Cd244	-3,224526076	4,00E-02
Slc5a7	-3,224526076	4,00E-02
Zglp1	-3,224526076	4,00E-02
Traf5	-3,219913595	1,64E-03
Rasgef1c	-3,218677094	6,26E-05
Ptprv	-3,134695472	1,28E-02
Vmn2r86	-3,115651197	1,68E-02
Kcnh4	-3,02774823	1,40E-04
Dkkl1	-2,983733289	3,82E-04
Abi3bp	-2,957375368	1,11E-04
Gm11549	-2,9476096	7,34E-05
2310014L17Rik	-2,940859601	1,02E-04
Slc22a13b-ps	-2,926047112	2,22E-02
Oprk1	-2,913448134	4,23E-04
Ccl27b	-2,834395366	1,84E-04
Hist1h3h	-2,827623997	1,44E-02
Rpl31-ps12	-2,762788559	2,22E-02
A830009L08Rik	-2,76236876	8,48E-03
Pou6f2	-2,749118946	1,83E-02
1700085C21Rik	-2,723102438	3,97E-02
Krt20	-2,693593754	2,91E-03
Egr2	-2,675449606	8,59E-04
Chrnb3	-2,673037676	2,27E-03

© 2023. Bellver-Sanchis A. et al. Published online at http://www.aginganddisease.org/EN/10.14336/AD.2023.0424-2

Neu2	-2,667198069	2,32E-02
Pappa2	-2,61255917	6,26E-04
Gm3985	-2,606436327	4,03E-03
Atp6ap1l	-2,597319206	3,02E-03
Col24a1	-2,584296822	6,66E-04
Adora2a	-2,580343929	2,97E-02
Gm20063	-2,580343929	2,97E-02
Exph5	-2,575561176	2,54E-04
Pld5	-2,572825251	1,65E-03
Sowahb	-2,567330995	4,80E-04
Rgs6	-2,551217565	3,67E-04
Tmem196	-2,550598717	1,81E-03
Gm3264	-2,507204987	3,97E-02
Xkr7	-2,475724991	5,47E-03
C730002L08Rik	-2,431806711	2,15E-03
Lamp5	-2,427609086	5,05E-04
AU023762	-2,42583759	3,82E-02
4933413L06Rik	-2,389176057	4,93E-02
Trhr2	-2,388753325	1,96E-02
1700007J10Rik	-2,388152841	4,93E-02
Jmjd7	-2,370999351	3,97E-02
St6gal2	-2,369023519	1,20E-03
Tnnc1	-2,3529406	5,91E-03
Samd15	-2,295804216	1,03E-02
Ankrd34c	-2,280873506	2,88E-03
Hs3st2	-2,272717812	1,40E-03
Kcnh5	-2,269901982	1,08E-03
Gpr88	-2,263528494	1,48E-03
Wisp1	-2,229016499	3,55E-03
Arhgap25	-2,226222407	3,13E-03
Arc	-2,221169172	1,21E-03
C130074G19Rik	-2,211405496	1,70E-03
Gm13308	-2,192470097	2,70E-03
Krt12	-2,192462277	4,07E-03
Tspan11	-2,181411363	7,47E-03
BC061194	-2,144416131	1,68E-02
Scn4b	-2,034037856	3,03E-03
Cbln2	-2,02891944	3,92E-03
Serpinb8	-2,021891763	6,98E-03
Stard8	-2,003007685	4,69E-03

© 2023. Bellver-Sanchis A. et al. Published online at http://www.aginganddisease.org/EN/10.14336/AD.2023.0424-2

SUPPLEMENTARY DATA

Adra1b	-1,991631158	6,41E-03
Fos	-1,987042966	5,52E-03
Car10	-1,979766778	3,87E-03
Foxp2	-1,973387257	5,86E-03
Gfra2	-1,961064736	4,30E-03
Osbpl3	-1,941149408	4,90E-03
Cckbr	-1,930930123	5,89E-03
Fhod3	-1,929933506	4,89E-03
4930429B21Rik	-1,91392892	1,12E-02
Hkdc1	-1,910930565	8,97E-03
Gpr3	-1,909737015	1,83E-02
Usp43	-1,900961817	3,23E-02
Mapk11	-1,891566276	7,05E-03
Pou3f2	-1,878678928	6,33E-03
Sema3a	-1,877945207	7,00E-03
Cobl	-1,859680603	6,29E-03
Satb2	-1,858770834	6,35E-03
Htr2a	-1,856972279	8,06E-03
Egr1	-1,854138555	6,21E-03
40238	-1,844633634	3,80E-02
4930447C04Rik	-1,763484395	1,95E-02
Mef2c	-1,761785966	8,89E-03
Kcnh7	-1,758381824	9,32E-03
Efna5	-1,726954354	1,23E-02
Adcyap1	-1,719312428	1,83E-02
Rasgef1b	-1,704204363	1,33E-02
Arpp19	-1,701397637	1,17E-02
Vmn2r87	-1,695084158	1,91E-02
Cpne5	-1,679116495	1,39E-02
Garnl3	-1,677050991	1,37E-02
Rims3	-1,672379039	1,53E-02
Igsf9b	-1,659970076	1,46E-02
Ipcef1	-1,659839925	1,42E-02
Pamr1	-1,657019084	1,77E-02
Camk2n1	-1,653683615	1,37E-02
Stx1a	-1,64574588	1,52E-02
Ptgfrn	-1,63762731	1,70E-02
Arhgap10	-1,625261678	1,87E-02
Bmp3	-1,624552224	2,59E-02
Grip2	-1,622521862	2,04E-02

© 2023. Bellver-Sanchis A. et al. Published online at http://www.aginganddisease.org/EN/10.14336/AD.2023.0424-2

SUPPLEMENTARY DATA

Hr	-1,60952456	1,91E-02
Medag	-1,608585008	2,13E-02
Gm13298	-1,608414111	1,74E-02
Cabyr	-1,599224655	2,86E-02
Rcan2	-1,596887545	1,77E-02
Ngef	-1,594846191	1,78E-02
Fam78a	-1,589164233	3,51E-02
Chrdl1	-1,58633467	2,93E-02
Rassf3	-1,579060866	2,38E-02
Mybpc1	-1,578174729	3,98E-02
Mkx	-1,575380228	2,52E-02
Lrrc75a	-1,574448832	3,05E-02
E130012A19Rik	-1,56836578	2,20E-02
6430584L05Rik	-1,557203672	4,03E-02
Mtcl1	-1,556561004	2,08E-02
9130024F11Rik	-1,553879452	2,54E-02
Scube1	-1,550612397	2,20E-02
Gm12429	-1,542070281	3,14E-02
Igfbp6	-1,534829786	2,80E-02
Plcb4	-1,522338966	2,49E-02
Syt6	-1,521533946	2,97E-02
Rgs4	-1,518343565	2,33E-02
BC030499	-1,515752203	3,71E-02
Lipg	-1,507916775	4,95E-02
Gcnt4	-1,506121576	3,12E-02
AI414108	-1,504741516	2,59E-02
Tmem132d	-1,496932664	2,74E-02
Coro6	-1,49077425	3,35E-02
Herc6	-1,476398811	3,41E-02
Tbr1	-1,469527657	2,88E-02
Gm3893	-1,465632753	2,80E-02
Cabp1	-1,461096113	3,05E-02
Dusp1	-1,456942559	3,47E-02
Nr4a1	-1,455995478	3,15E-02
Gabra3	-1,448176395	3,10E-02
Fam124a	-1,431013821	3,98E-02
Ephb6	-1,425342563	3,40E-02
Oprd1	-1,416318281	3,97E-02
Snurf	-1,415951731	3,66E-02
Frmd6	-1,412650518	3,67E-02

© 2023. Bellver-Sanchis A. et al. Published online at http://www.aginganddisease.org/EN/10.14336/AD.2023.0424-2

Kcnh1	-1,40845046	3,59E-02
Kcnab3	-1,397977232	3,88E-02
Adrb1	-1,397134587	4,49E-02
Cux1	-1,39425797	3,75E-02
Trib1	-1,393503557	4,43E-02
Myo1b	-1,38344639	4,22E-02
Pdzrn3	-1,380468319	4,39E-02
Plxnd1	-1,37860681	4,15E-02
Rorb	-1,371766378	4,09E-02
Arhgap42	-1,370386924	4,55E-02
Asb13	-1,368731406	4,36E-02
Zfp365	-1,361238403	4,10E-02
Cux2	-1,358237863	4,29E-02
Camkk2	-1,350480895	4,29E-02
Olfm2	-1,346795264	4,92E-02
Ccl27a	-1,341650988	4,63E-02
Camk4	-1,327173604	4,62E-02
Laptm4a	-1,22959717	6,50E-03
Cadm1	-1,15159789	2,56E-02
Clta	-1,10074302	1,89E-02
Sumo2	-1,09268279	3,72E-02
Nfib	-1,04139882	5,89E-03
Tmsb4x	-1,00403317	4,42E-02
Gmfb	-0,9570028	3,47E-02
Tmsb4xp8	-0,94680387	4,29E-02
Actr2	-0,93045666	4,39E-02
Znf706	-0,85797147	1,36E-02
Etv1	-0,8356197	1,95E-02
Rab10	-0,62599276	4,50E-03
Eif4g3	-0,58949328	3,93E-02
Ppp3ca	-0,58501205	3,33E-02
Ik	-0,57854631	9,05E-03
Ptbp2	-0,55903414	2,22E-02
Ac046176.1	-0,44269435	4,29E-02
Cacn2d1	0,5567782	2,65E-02
Pak5	0,60365478	4,81E-02
Pias2	0,72110603	4,81E-02
Pcdh7	0,79407177	1,73E-04
Cxxc4	0,81488799	1,68E-02
Camk2g	0,82033783	3,31E-02

© 2023. Bellver-Sanchis A. et al. Published online at http://www.aginganddisease.org/EN/10.14336/AD.2023.0424-2

SUPPLEMENTARY DATA

R3hdm2	0,8249999	1,09E-03
Strbp	0,96156862	4,72E-03
Nlk	0,98139251	2,29E-02
R3hdm1	1,02742384	3,03E-02
Clk3	1,06161232	7,01E-04
Atp2b2	1,10097339	2,88E-02
Snap25	1,17876809	1,75E-02
Kctd12	1,314599306	4,95E-02
Cacna1h	1,321380915	4,93E-02
Arhgef26	1,340309119	4,80E-02
Nos1	1,357523454	4,52E-02
Tgfbr3	1,361723754	4,67E-02
Scn3b	1,36577581	4,13E-02
Rgs14	1,365999949	4,54E-02
Orai2	1,373671602	4,07E-02
Gfap	1,388464381	3,80E-02
Abca8a	1,389298438	4,95E-02
Lrrtm1	1,389856521	4,13E-02
Sh3d19	1,398160688	4,05E-02
Nr4a3	1,404884869	4,45E-02
Adamtsl1	1,410432647	4,85E-02
Aankrd52	1,41658774	5,91E-03
Golm1	1,417604621	3,68E-02
Fabp7	1,420098959	4,35E-02
Ptpn14	1,430279612	4,10E-02
Thsd7b	1,432412584	4,14E-02
Gm13826	1,436431364	3,44E-02
Dock4	1,442448871	3,09E-02
Cyp7b1	1,4527982	3,92E-02
Foxo1	1,461309285	3,26E-02
Clmn	1,46686559	2,88E-02
Zfp608	1,467426171	3,04E-02
Ogn	1,468064174	4,89E-02
Bhlhe22	1,470806899	3,05E-02
Cgnl1	1,474375015	3,28E-02
Gsta4	1,489770552	3,11E-02
Hba-a1	1,490337224	2,64E-02
Ehd1	1,490725238	3,06E-02
Kcng2	1,500711283	4,53E-02
Rac3	1,501518798	4,77E-02

© 2023. Bellver-Sanchis A. et al. Published online at http://www.aginganddisease.org/EN/10.14336/AD.2023.0424-2

Sertm1	1,507861696	2,74E-02
Fat4	1,508719203	2,52E-02
Myof	1,512484109	3,58E-02
Cnih2	1,517113199	2,44E-02
Cd44	1,520885861	4,96E-02
Crim1	1,528166045	2,35E-02
Htr2c	1,532213836	2,48E-02
Wbscr17	1,533268231	2,30E-02
Nrp1	1,536230503	2,27E-02
Hspb8	1,546246462	4,31E-02
Pip5k1b	1,54800703	2,57E-02
Gria1	1,552777109	2,03E-02
Necab2	1,555117414	2,47E-02
Mr1	1,556333133	4,75E-02
Fzd7	1,556823989	2,87E-02
Car12	1,56052631	2,35E-02
Abca4	1,567315877	3,82E-02
Txnip	1,567521635	2,92E-02
Crtac1	1,570075721	2,19E-02
Fbn1	1,572265918	2,32E-02
Marcksl1	1,575096909	2,30E-02
Pm20d2	1,577273437	2,90E-02
Tenm2	1,578060239	1,89E-02
Cntnap5c	1,580978784	2,22E-02
Pcdh19	1,592734257	1,84E-02
Ccbe1	1,594951246	2,40E-02
Cdh23	1,596398234	4,97E-02
Spidr	1,597960964	4,97E-02
Slc17a6	1,598820985	2,11E-02
Kit	1,602852343	1,82E-02
Dbi	1,605602181	1,91E-02
Cdc42bpg	1,607740001	3,98E-02
Il17rd	1,611729411	2,93E-02
Samd11	1,618113219	4,71E-02
Ryr3	1,619086178	1,63E-02
Tcf7l2	1,62572914	2,30E-02
Rpl34	1,627195298	2,85E-02
2610018G03Rik	1,627335681	4,48E-02
Icosl	1,627335681	4,48E-02
Svep1	1,627335681	4,48E-02

© 2023. Bellver-Sanchis A. et al. Published online at http://www.aginganddisease.org/EN/10.14336/AD.2023.0424-2

SUPPLEMENTARY DATA

Ass1	1,628059642	2,77E-02
Mt2	1,630902141	1,80E-02
Gdpd2	1,638842505	4,29E-02
A330050F15Rik	1,645666076	3,03E-02
Rreb1	1,64906498	1,49E-02
Evc	1,667852592	2,64E-02
Pde3a	1,66959951	4,97E-02
Pigr	1,677854352	3,89E-02
Efcab1	1,67920105	4,22E-02
Cdhr1	1,687624519	2,99E-02
Dgkg	1,690630932	1,24E-02
Hist4h4	1,706866561	1,70E-02
Sipa1l3	1,717846622	1,13E-02
Dock10	1,721396947	1,12E-02
Slc16a12	1,725192307	2,86E-02
Fgl2	1,72831582	4,95E-02
Man1a	1,728701905	1,15E-02
Scn5a	1,733695853	4,67E-02
Spef2	1,733695853	4,67E-02
Gm20754	1,738092944	3,95E-02
Pwwp2b	1,741418367	1,93E-02
Mc4r	1,74484941	3,30E-02
Ntng1	1,747251097	1,09E-02
Nrp2	1,748684236	1,04E-02
Arsj	1,751806582	2,36E-02
Mum1l1	1,751806582	2,36E-02
Arhgap12	1,757303884	9,78E-03
Wipf3	1,763923057	8,94E-03
Mycl	1,764278642	1,37E-02
Gpc4	1,764613729	1,21E-02
Gabra5	1,76693662	9,50E-03
Lpl	1,769577149	9,74E-03
Rbp1	1,774204212	2,65E-02
Il1r1	1,779089412	1,38E-02
Gfra1	1,780769921	9,97E-03
Pcdh8	1,793113432	1,02E-02
Car14	1,796370325	2,41E-02
Trpc4	1,796786576	9,05E-03
Efemp2	1,796799947	2,17E-02
Epha6	1,799844348	7,87E-03

© 2023. Bellver-Sanchis A. et al. Published online at http://www.aginganddisease.org/EN/10.14336/AD.2023.0424-2

Baiap3	1,803358199	2,44E-02
Spint2	1,803906809	1,48E-02
Nr2f2	1,804837999	9,85E-03
Efcab7	1,805603867	3,65E-02
Gm13315	1,810498417	1,84E-02
Zmat5	1,820398379	1,20E-02
Vwc2l	1,826482194	1,10E-02
Pde11a	1,8336279	1,38E-02
Bambi	1,835096877	2,81E-02
Kcnj13	1,845257078	1,09E-02
Efs	1,849508765	1,84E-02
Dpyd	1,854885113	1,22E-02
Zic1	1,858078146	9,90E-03
Drd5	1,867400793	1,71E-02
Cpq	1,867729146	1,36E-02
Tshz2	1,869612826	6,42E-03
Rerg	1,884081883	1,12E-02
Lpar4	1,887209102	2,23E-02
Col4a3	1,89595633	3,48E-02
Crispld2	1,89595633	3,48E-02
Iqgap2	1,898823931	5,40E-03
C1qtnf5	1,908380631	1,49E-02
Gm15713	1,911294901	4,22E-02
Myo7a	1,91675504	1,19E-02
Cachd1	1,917071556	6,14E-03
Rpl26	1,917948419	8,06E-03
Ctxn2	1,920270718	2,95E-02
Hist2h3b	1,940981538	2,75E-02
Prdm5	1,941250233	8,79E-03
Cpne2	1,945159093	7,25E-03
St18	1,948072887	5,34E-03
Rem2	1,954552738	1,50E-02
4930509J09Rik	1,956903912	3,63E-02
A4galt	1,959308722	4,48E-02
Thbs3	1,96151831	1,08E-02
Gm17762	1,962432428	3,23E-02
Cd38	1,988274065	1,52E-02
Gm14092	1,998842623	4,57E-02
Ubxn10	1,998842623	4,57E-02
Wdr52	1,99902718	7,76E-03

© 2023. Bellver-Sanchis A. et al. Published online at http://www.aginganddisease.org/EN/10.14336/AD.2023.0424-2

SUPPLEMENTARY DATA

Aloxe3	2,001115126	3,12E-02
Fzd10	2,001115126	3,12E-02
Itga8	2,005414525	4,60E-03
Fbn2	2,011835915	7,35E-03
Ky	2,014164962	2,64E-02
Kctd4	2,019710386	3,66E-03
Prelp	2,021900917	4,98E-03
Slit1	2,025578174	3,06E-03
D630023F18Rik	2,032326604	1,60E-02
Car4	2,044751843	4,03E-03
Zfp503	2,051509255	2,31E-02
Rnf182	2,057150615	3,71E-03
Col9a3	2,058913103	4,50E-03
Cyp2e1	2,05964904	2,02E-02
Crym	2,060589138	2,96E-03
Hsd11b1	2,061971529	9,37E-03
Myo5b	2,062478747	2,84E-03
Raver1-fdx1l	2,062516706	2,02E-02
Fbxl7	2,072336316	3,65E-02
Trpm3	2,072989999	2,48E-03
Nr3c2	2,07923315	2,43E-03
Ccdc78	2,079963501	3,65E-02
Fgf10	2,08017776	4,08E-03
Serpina3n	2,082235886	2,70E-03
Vamp8	2,082689285	3,23E-02
Zic4	2,08566951	2,33E-02
4933412O06Rik	2,087911228	2,02E-02
Zfp185	2,089658684	1,58E-02
Col8a1	2,112600755	6,82E-03
Chst8	2,119106751	3,77E-03
St6galnac5	2,12068174	2,23E-03
Cd248	2,123417269	1,77E-02
Nek10	2,12615815	2,02E-02
Ttc21a	2,12615815	2,02E-02
Cyp2j12	2,129787371	2,75E-02
Zbtb20	2,132784333	2,05E-03
Ust	2,138764639	2,29E-03
Mdga1	2,139746487	2,16E-03
Tnfaip8l3	2,146021151	2,99E-03
Cd14	2,154410855	4,93E-02

© 2023. Bellver-Sanchis A. et al. Published online at http://www.aginganddisease.org/EN/10.14336/AD.2023.0424-2

SUPPLEMENTARY DATA

Dach2	2,154410855	4,93E-02
Efhc1	2,154410855	4,93E-02
Gm20751	2,154410855	4,93E-02
Sdc1	2,154410855	4,93E-02
Serpinb1b	2,154410855	4,93E-02
Glb1l2	2,165541443	2,02E-02
Col6a5	2,165963172	2,93E-02
Hopx	2,174351615	2,67E-03
Ackr4	2,17600078	2,93E-02
Amigo2	2,182406338	2,93E-03
Clec1a	2,188993254	3,35E-02
Prox1	2,198285179	1,67E-03
Fam160a1	2,200677224	6,00E-03
Csrp2	2,219607596	2,01E-02
Pon3	2,224975374	1,20E-02
Aard	2,241166426	3,82E-02
Gm16998	2,241166426	3,82E-02
Npbwr1	2,241166426	3,82E-02
Stk33	2,241166426	3,82E-02
Prokr2	2,241205379	2,81E-02
Mamdc2	2,241232006	8,73E-03
Ace	2,248521334	1,34E-03
Slc9a2	2,256242367	2,36E-03
Gm21949	2,259219636	2,36E-03
Grp	2,269725303	8,73E-03
Thsd4	2,278168163	1,51E-03
Hist1h2ac	2,291269208	1,99E-02
Gpnmb	2,291593724	2,36E-02
Ecel1	2,304161979	1,48E-02
Tmem255a	2,309782987	1,36E-03
Trpv4	2,313130449	1,15E-02
Gm14685	2,314221849	2,97E-02
Lamb1	2,320208453	1,08E-03
E030013I19Rik	2,32299961	2,97E-02
Rasd1	2,341053926	1,81E-03
Card6	2,351976732	6,20E-03
Slc44a5	2,369505493	1,09E-03
Cpne6	2,391071281	5,51E-04
Figf	2,39233661	1,55E-02
Tanc1	2,39576864	5,55E-04

© 2023. Bellver-Sanchis A. et al. Published online at http://www.aginganddisease.org/EN/10.14336/AD.2023.0424-2

Egf	2,400439122	2,32E-02
Sec14l3	2,400439122	2,32E-02
Col11a1	2,427926047	5,93E-04
Ly6a	2,432988505	1,42E-02
Pcdha4	2,440238373	1,53E-03
Enpp2	2,457199985	3,67E-04
Fam161a	2,465307247	5,07E-03
Sema3b	2,478627816	5,91E-03
Msx1	2,486825244	3,56E-03
Rxfp3	2,502796227	1,03E-02
Tgfb2	2,510147495	4,38E-04
Col5a2	2,511182319	1,32E-03
Lum	2,519617242	4,01E-03
Dnah9	2,521046696	3,91E-04
Efhb	2,521932429	3,97E-02
Hoga1	2,521932429	3,97E-02
Myoc	2,521932429	3,97E-02
Ebf3	2,543863377	1,44E-02
Glis3	2,546897161	7,43E-04
Rph3al	2,561754104	8,74E-03
Sec1	2,565135862	1,13E-02
Cdkn1c	2,579962723	9,09E-04
Pkp2	2,598231944	4,97E-04
Adamts9	2,598897692	1,01E-03
9930014A18Rik	2,600109721	1,92E-03
Crhr2	2,610560385	1,14E-02
Zbbx	2,610560385	1,14E-02
Cidea	2,620553043	2,96E-02
Col2a1	2,620553043	2,96E-02
Pcdh20	2,637568267	1,75E-04
Gramd1c	2,641626037	7,46E-03
Npr1	2,647069141	2,28E-03
Ndst4	2,653975606	3,52E-04
Nnat	2,673915291	1,48E-04
Trp73	2,701182468	4,77E-03
Espl1	2,712861293	2,22E-02
Nmbr	2,712861293	2,22E-02
Frem3	2,717307028	5,47E-03
Dqx1	2,726990723	1,68E-02
Tgtp1	2,732129274	3,42E-03

© 2023. Bellver-Sanchis A. et al. Published online at http://www.aginganddisease.org/EN/10.14336/AD.2023.0424-2

Olfml2b	2,733154203	2,24E-04
Wdr96	2,761669883	1,95E-03
Foxj1	2,764169838	1,33E-03
Gstm6	2,789215043	4,05E-03
Cldn26	2,793933109	7,29E-03
A2m	2,80324447	7,33E-04
Klk8	2,808472316	1,20E-03
Lmo1	2,808472316	1,20E-03
Eomes	2,844331315	1,28E-02
Tnxb	2,850918718	1,37E-04
Xist	2,853211356	2,76E-03
Slc2a12	2,855868508	2,75E-04
Igsf1	2,860975203	1,23E-03
Dnaic2	2,881450049	1,28E-02
Lrrc10b	2,896702224	1,68E-04
Myh8	2,920857723	2,27E-03
Gpx8	2,954229962	1,27E-03
Col4a4	2,956604852	3,83E-03
Arhgap28	2,958889561	9,78E-03
Grem1	2,958889561	9,78E-03
Il20rb	2,958889561	9,78E-03
Stpg1	3,032383254	7,53E-03
Ppl	3,045602891	1,31E-03
Gpr161	3,05499536	8,99E-05
Homer3	3,068169327	1,08E-04
Dnah11	3,07807118	4,44E-04
Slc29a4	3,081356135	1,07E-04
Lrrc18	3,101013938	4,00E-02
Plek2	3,101013938	4,00E-02
Slc39a4	3,101013938	4,00E-02
Ttc16	3,101013938	4,00E-02
Zfp345	3,114890244	1,98E-02
Doc2b	3,146985114	1,67E-05
Slc26a4	3,154500402	1,06E-04
Gm2115	3,158260507	2,68E-05
Cpne7	3,189280277	1,09E-05
1700026D08Rik	3,225092539	2,80E-02
Dgkk	3,225092539	2,80E-02
Gck	3,225092539	2,80E-02
Umodl1	3,225092539	2,80E-02

© 2023. Bellver-Sanchis A. et al. Published online at http://www.aginganddisease.org/EN/10.14336/AD.2023.0424-2

SUPPLEMENTARY DATA

Stra6	3,238742355	2,12E-04
Calb2	3,240373842	2,86E-05
Shisa6	3,252776424	6,68E-06
Cd109	3,275206174	1,93E-04
Hbq1a	3,278772013	1,98E-02
Spag16	3,287353315	1,18E-03
Stac	3,293811338	3,57E-03
Prkcd	3,299168623	1,50E-05
Caps2	3,33933976	1,98E-02
Cndp1	3,33933976	1,98E-02
Fhl4	3,33933976	1,98E-02
Ppic	3,33933976	1,98E-02
Prph	3,33933976	1,98E-02
Agt	3,39908875	3,88E-05
Spink8	3,416966559	2,22E-04
Cabp7	3,427139509	1,27E-05
Rbm47	3,444907994	2,22E-04
Ccl6	3,445199846	1,42E-02
Plcz1	3,445199846	1,42E-02
Egfl6	3,449276862	1,42E-02
Mdfic	3,493698643	1,04E-04
Nts	3,51505529	1,42E-03
Popdc3	3,51505529	1,42E-03
Dynlrb2	3,54382046	1,02E-02
Cldn1	3,55155745	1,29E-04
Fermt1	3,566928427	1,14E-03
Gmnc	3,573107949	2,91E-04
Htr4	3,606232028	1,68E-05
Ccdc153	3,63612871	7,45E-03
Tmem255b	3,722884281	5,48E-03
Vit	3,751049631	4,97E-04
Il16	3,778064678	2,02E-06
Fam212a	3,804717466	4,07E-03
Gja4	3,804717466	4,07E-03
Usp51	3,804717466	4,07E-03
Wdr63	3,804717466	4,07E-03
Lgr6	3,831055529	3,29E-05
2310002F09Rik	3,835604015	3,36E-04
Col8a2	3,872111422	2,69E-05
Mfrp	3,873086203	5,01E-05

© 2023. Bellver-Sanchis A. et al. Published online at http://www.aginganddisease.org/EN/10.14336/AD.2023.0424-2

Lct	3,881980931	6,91E-07
Magel2	3,882156978	3,04E-03
Dcn	3,894310231	4,96E-07
Calml4	3,95315395	4,85E-05
Meig1	3,955650671	2,30E-03
Tuft1	3,955650671	2,30E-03
Cebpd	4,002419518	3,70E-05
Aqp1	4,003660879	3,70E-05
Dlk1	4,003660879	3,70E-05
Wdr16	4,025581232	1,74E-03
Cox6b2	4,092278241	1,33E-03
Gpr101	4,122695007	1,94E-05
Dsc3	4,131558388	9,50E-05
Fibcd1	4,175764988	7,82E-08
Npy2r	4,217207525	2,41E-07
Lbp	4,235389899	9,19E-06
Chst9	4,275650965	7,97E-04
Serpina3h	4,372220136	4,88E-04
Capsl	4,386110582	4,88E-04
Prlr	4,461503752	2,16E-08
Ntf3	4,584496823	1,95E-04
C3	4,630105833	1,57E-04
1500015O10Rik	4,661208562	9,29E-07
Crlf1	4,758871432	8,44E-05
C1ql2	4,853266464	2,43E-06
Slc9a4	5,01436128	1,48E-07
Slitrk6	5,020985524	2,70E-05
Epn3	5,087890476	1,89E-05
Kl	5,530406846	2,68E-11
F5	5,596176696	5,23E-10
Dsp	5,615867008	3,66E-11
Clic6	5,621113392	4,41E-10
Arhgef16	5,729836039	3,48E-02
Ccdc33	5,729836039	3,48E-02
Clec4a3	5,729836039	3,48E-02
Cox8b	5,729836039	3,48E-02
Dmrt3	5,729836039	3,48E-02
Gm16551	5,729836039	3,48E-02
Nespas	5,729836039	3,48E-02
Slc16a8	5,729836039	3,48E-02

© 2023. Bellver-Sanchis A. et al. Published online at http://www.aginganddisease.org/EN/10.14336/AD.2023.0424-2

Slc38a4	5,729836039	3,48E-02
Slco1a5	5,729836039	3,48E-02
Tmem102	5,729836039	3,48E-02
Tmem212	5,729836039	3,48E-02
Hist1h2ap	5,751910039	3,48E-02
Ccdc135	5,776533881	5,12E-07
Gm8989	5,863023466	3,48E-02
4933427G17Rik	5,919079041	2,19E-02
Avpr1b	5,919079041	2,19E-02
Cldn19	5,919079041	2,19E-02
Pemt	5,919079041	2,19E-02
Tbx4	5,919079041	2,19E-02
Fam227b	5,961028766	2,19E-02
Tcfl5	5,986641127	2,19E-02
Nup62-il4i1	6,020098623	2,19E-02
Avpr1a	6,086352426	2,19E-02
Enpp3	6,086352426	2,19E-02
Gm4951	6,086352426	2,19E-02
Prom2	6,086352426	2,19E-02
Rab38	6,086352426	2,19E-02
Trp63	6,086352426	2,19E-02
Tmem140	6,089507778	2,19E-02
Folr1	6,101968665	7,86E-08
Naip6	6,234806451	1,41E-02
Gm19299	6,236230711	1,41E-02
Igbp1b	6,236230711	1,41E-02
Iqch	6,236230711	1,41E-02
Klk10	6,236230711	1,41E-02
Lgals3	6,236230711	1,41E-02
Sec14l4	6,236230711	1,41E-02
Pmch	6,301632819	9,22E-03
Mpzl3	6,371993425	9,22E-03
Pifo	6,371993425	9,22E-03
Ripk4	6,371993425	9,22E-03
Steap4	6,371993425	9,22E-03
Gstp2	6,410350159	6,15E-03
Slc4a5	6,496079135	7,70E-09
Catsperg2	6,509091297	6,15E-03
Krt8	6,610319247	4,17E-03
Scin	6,610319247	4,17E-03

© 2023. Bellver-Sanchis A. et al. Published online at http://www.aginganddisease.org/EN/10.14336/AD.2023.0424-2

Hist2h2aa2	6,672660301	2,86E-03
Kcnh8	6,716179333	2,86E-03
Nek5	6,716179333	2,86E-03
Spint1	6,716179333	2,86E-03
Chrm5	6,814799947	2,00E-03
Lrp2	6,814799947	2,00E-03
Hist2h3c1	6,905318745	1,41E-03
Best3	6,907108198	1,41E-03
Serpina3g	6,941566087	1,41E-03
Krt2	6,993863769	1,01E-03
Wdr86	6,993863769	1,01E-03
Apoc1	7,075696953	7,27E-04
Oca2	7,075696953	7,27E-04
Gm11992	7,153136465	5,30E-04
Ccdc146	7,226630158	3,91E-04
Iyd	7,226630158	3,91E-04
Tmprss11a	7,226630158	3,91E-04
Wfdc2	7,226630158	3,91E-04
Zcchc5	7,363257728	2,18E-04
Prr32	7,602917167	9,64E-05
Krt18	7,657090069	7,45E-05
Tdo2	7,709302194	5,79E-05
Steap1	7,808378224	3,57E-05
Rsc1a1	7,878004917	2,83E-05
Lrrc23	8,070339558	9,48E-06
Rdh5	8,291965011	3,54E-06
Gm3317	8,292649654	3,54E-06
Jmjd7-pla2g4b	8,48043344	1,22E-06
Otx2	8,513699706	1,03E-06
Cldn2	8,852358284	1,68E-07
Kcne2	8,985349371	8,64E-08
Rnaset2b	9,294967611	1,47E-08
Lhx9	9,356998414	9,62E-09
Ttr	9,445444196	1,16E-22
Sostdc1	9,638315625	1,74E-09
Tmem72	10,12514828	9,25E-11

Supplementary Table. 8 | H3K9me2 peaks at TSS in AML12 cells treated with UNC0638.

© 2023. Bellver-Sanchis A. et al. Published online at http://www.aginganddisease.org/EN/10.14336/AD.2023.0424-2

SUPPLEMENTARY DATA

Chr	Star	End	Peak	Scor e	TSS
chr1	32172176	32173098	H3K9ME2_UNC0638_peak_1	59	*Khdrbs2*
chr1	42952750	42953467	H3K9ME2_UNC0638_peak_2	32	*Gpr45*
chr1	69826896	69827244	H3K9ME2_UNC0638_peak_3	38	*Spag16*
chr1	86703769	86704122	H3K9ME2_UNC0638_peak_4	32	*Dis3l2*
chr1	109983282	109983585	H3K9ME2_UNC0638_peak_5	35	*Cdh7*
chr1	152955282	152955573	H3K9ME2_UNC0638_peak_6	17	*Nmnat2*
chr1	181352292	181353448	H3K9ME2_UNC0638_peak_7	55	*Cnih3*
chr1	33907545	33908234	H3K9ME2_UNC0638_peak_8	36	*Dst*
chr1	46425308	46425654	H3K9ME2_UNC0638_peak_9	32	*Dnah7c*
chr1	55932595	55932886	H3K9ME2_UNC0638_peak_10	27	*9130227L01Rik*
chr1	61306509	61307159	H3K9ME2_UNC0638_peak_11	44	*Gm11587*
chr1	63272940	63273933	H3K9ME2_UNC0638_peak_12	47	*Zdbf2*
chr1	66467924	66468734	H3K9ME2_UNC0638_peak_13	67	-
chr1	75359938	75360478	H3K9ME2_UNC0638_peak_14	28	*Des*
chr1	78310141	78310538	H3K9ME2_UNC0638_peak_15	27	*Sgpp2*
chr1	88318028	88318504	H3K9ME2_UNC0638_peak_16	50	*Trpm8*
chr1	125911610	125912180	H3K9ME2_UNC0638_peak_17	30	*Nckap5*
chr1	132880196	132881157	H3K9ME2_UNC0638_peak_18	41	*Lrrn2*
chr1	134560228	134560814	H3K9ME2_UNC0638_peak_19	27	*Kdm5b*
chr1	151571373	151571664	H3K9ME2_UNC0638_peak_20	17	*Niban1*
chr1	171558852	171559405	H3K9ME2_UNC0638_peak_21	49	*Cd244a*
chr1	174501679	174501970	H3K9ME2_UNC0638_peak_22	38	*Fmn2*
chr1	178530181	178530552	H3K9ME2_UNC0638_peak_23	27	*Kif26b*
chr1	185455088	185455402	H3K9ME2_UNC0638_peak_24	23	*Slc30a10*
chr1	5588745	5589103	H3K9ME2_UNC0638_peak_25	38	*Oprk1*
chr1	12668530	12669260	H3K9ME2_UNC0638_peak_26	48	*Gm17644*
chr1	13844932	13845233	H3K9ME2_UNC0638_peak_27	40	*Gm31195*
chr1	17097006	17097301	H3K9ME2_UNC0638_peak_28	36	-
chr1	17098085	17098400	H3K9ME2_UNC0638_peak_29	38	-
chr1	17727613	17728196	H3K9ME2_UNC0638_peak_30	42	*Crispld1*
chr1	34432350	34432641	H3K9ME2_UNC0638_peak_31	32	*Mir5103*
chr1	34552393	34552789	H3K9ME2_UNC0638_peak_32	40	*Prss40*
chr1	36778555	36778879	H3K9ME2_UNC0638_peak_33	35	*Zap70*
chr1	39650357	39651172	H3K9ME2_UNC0638_peak_34	50	-
chr1	42697541	42698155	H3K9ME2_UNC0638_peak_35	28	*Pou3f3*
chr1	43092196	43092718	H3K9ME2_UNC0638_peak_36	32	-
chr1	54283281	54283777	H3K9ME2_UNC0638_peak_37	25	-
chr1	75375352	75375752	H3K9ME2_UNC0638_peak_38	27	*Speg*

SUPPLEMENTARY DATA

chr1	79436072	79436448	H3K9ME2_UNC0638_peak_39	27	*Scg2*
chr1	85112336	85112656	H3K9ME2_UNC0638_peak_40	94	-
chr1	85246996	85247295	H3K9ME2_UNC0638_peak_41	20	*C130026I21Rik*
chr1	88205406	88205697	H3K9ME2_UNC0638_peak_42	22	*Dnajb3*
chr1	89931250	89931600	H3K9ME2_UNC0638_peak_43	27	-
chr1	92831081	92832134	H3K9ME2_UNC0638_peak_44	63	*Gpc1*
chr1	92932595	92932886	H3K9ME2_UNC0638_peak_45	44	*9430060I03Rik*
chr1	93804024	93804315	H3K9ME2_UNC0638_peak_46	50	*Ing5*
chr1	104304415	104304706	H3K9ME2_UNC0638_peak_47	25	*Gm41924*
chr1	105780310	105780783	H3K9ME2_UNC0638_peak_48	22	*Tnfrsf11a*
chr1	120114600	120114913	H3K9ME2_UNC0638_peak_49	22	*Dbi*
chr1	120340534	120341649	H3K9ME2_UNC0638_peak_50	29	*C1ql2*
chr1	130422730	130423060	H3K9ME2_UNC0638_peak_51	32	-
chr1	134298844	134299371	H3K9ME2_UNC0638_peak_52	37	-
chr1	134492941	134493408	H3K9ME2_UNC0638_peak_53	27	*Rabif*
chr1	148711936	148712267	H3K9ME2_UNC0638_peak_54	28	*Gm41966*
chr1	150361741	150362280	H3K9ME2_UNC0638_peak_55	50	-
chr1	151344453	151344786	H3K9ME2_UNC0638_peak_56	38	-
chr1	155244012	155245088	H3K9ME2_UNC0638_peak_57	94	*Gm31102*
chr1	171064454	171064772	H3K9ME2_UNC0638_peak_58	88	*Mir6546*
chr1	171517893	171518533	H3K9ME2_UNC0638_peak_59	45	*Itln1*
chr1	180813983	180814274	H3K9ME2_UNC0638_peak_60	27	-
chr1	185363133	185363509	H3K9ME2_UNC0638_peak_61	38	-
chr1	192855211	192855663	H3K9ME2_UNC0638_peak_62	27	*Gm15867*
chr2	19909691	19909983	H3K9ME2_UNC0638_peak_63	29	-
chr2	31314101	31314466	H3K9ME2_UNC0638_peak_64	17	-
chr2	55436192	55436925	H3K9ME2_UNC0638_peak_65	43	-
chr2	156196944	156197268	H3K9ME2_UNC0638_peak_66	72	*Phf20*
chr2	4717531	4717822	H3K9ME2_UNC0638_peak_67	34	*Bend7*
chr2	4718219	4718714	H3K9ME2_UNC0638_peak_68	55	*Bend7*
chr2	11172175	11172547	H3K9ME2_UNC0638_peak_69	32	*Prkcq*
chr2	14513140	14513746	H3K9ME2_UNC0638_peak_70	38	-
chr2	21877679	21877991	H3K9ME2_UNC0638_peak_71	34	*Gm39767*
chr2	24626125	24626421	H3K9ME2_UNC0638_peak_72	21	*Cacna1b*
chr2	49787570	49788011	H3K9ME2_UNC0638_peak_73	20	*Lypd6b*
chr2	50065985	50066545	H3K9ME2_UNC0638_peak_74	28	*Lypd6*
chr2	70509116	70509621	H3K9ME2_UNC0638_peak_75	27	*Erich2*
chr2	71980942	71981612	H3K9ME2_UNC0638_peak_76	51	*Rapgef4*
chr2	75137654	75138154	H3K9ME2_UNC0638_peak_77	27	*2600014E21Rik*
chr2	79255078	79255957	H3K9ME2_UNC0638_peak_78	72	*Itga4*

© 2023. Bellver-Sanchis A. et al. Published online at http://www.aginganddisease.org/EN/10.14336/AD.2023.0424-2

SUPPLEMENTARY DATA

chr2	106693390	106694023	H3K9ME2_UNC0638_peak_79	31	*Mpped2*
chr2	110362142	110362495	H3K9ME2_UNC0638_peak_80	27	*Fibin*
chr2	133552639	133552992	H3K9ME2_UNC0638_peak_81	32	*Bmp2*
chr2	135659643	135659956	H3K9ME2_UNC0638_peak_82	43	*Plcb4*
chr2	156613736	156614494	H3K9ME2_UNC0638_peak_83	42	-
chr2	156721041	156721378	H3K9ME2_UNC0638_peak_84	64	*Dlgap4*
chr2	157913978	157915252	H3K9ME2_UNC0638_peak_85	125	*Vstm2l*
chr2	165111874	165112247	H3K9ME2_UNC0638_peak_86	21	*Cdh22*
chr2	167096733	167097026	H3K9ME2_UNC0638_peak_87	28	*Kcnb1*
chr2	177320997	177321365	H3K9ME2_UNC0638_peak_88	68	-
chr2	177464802	177465255	H3K9ME2_UNC0638_peak_89	27	*Zfp970*
chr2	14417361	14417896	H3K9ME2_UNC0638_peak_90	30	-
chr2	19445260	19445551	H3K9ME2_UNC0638_peak_91	27	*Ptf1a*
chr2	25706651	25707280	H3K9ME2_UNC0638_peak_92	50	*Bmyc*
chr2	28446924	28447360	H3K9ME2_UNC0638_peak_93	26	-
chr2	31541707	31542253	H3K9ME2_UNC0638_peak_94	43	*Gm32952*
chr2	31670018	31670418	H3K9ME2_UNC0638_peak_95	27	*Exosc2*
chr2	42871218	42871601	H3K9ME2_UNC0638_peak_96	23	*Gm39803*
chr2	56786358	56786649	H3K9ME2_UNC0638_peak_97	17	*Mir195b*
chr2	74668618	74669122	H3K9ME2_UNC0638_peak_98	25	*Hoxd13*
chr2	75434723	75435341	H3K9ME2_UNC0638_peak_99	27	*9430019J16Rik*
chr2	84872034	84872802	H3K9ME2_UNC0638_peak_100	48	*Rtn4rl2*
chr2	85136579	85136977	H3K9ME2_UNC0638_peak_101	22	*Aplnr*
chr2	90889285	90889763	H3K9ME2_UNC0638_peak_102	17	*C1qtnf4*
chr2	92600285	92600606	H3K9ME2_UNC0638_peak_103	29	-
chr2	93642614	93642949	H3K9ME2_UNC0638_peak_104	28	*Alx4*
chr2	93956501	93957010	H3K9ME2_UNC0638_peak_105	51	*Gm13889*
chr2	119594351	119594644	H3K9ME2_UNC0638_peak_106	27	*Oip5os1*
chr2	126552899	126553313	H3K9ME2_UNC0638_peak_107	36	*Slc27a2*
chr2	131040889	131041807	H3K9ME2_UNC0638_peak_108	80	*Gfra4*
chr2	144458357	144458654	H3K9ME2_UNC0638_peak_109	32	*Zfp133-ps*
chr2	146221986	146223758	H3K9ME2_UNC0638_peak_110	43	*Insm1*
chr2	147084285	147085664	H3K9ME2_UNC0638_peak_111	76	*Nkx2-4*
chr2	148395112	148395459	H3K9ME2_UNC0638_peak_112	22	*Sstr4*
chr2	150667935	150668232	H3K9ME2_UNC0638_peak_113	46	*E130215H24Rik*
chr2	151701976	151702389	H3K9ME2_UNC0638_peak_114	38	*Tmem74b*
chr2	153031668	153032952	H3K9ME2_UNC0638_peak_115	63	*Xkr7*
chr2	157078794	157079770	H3K9ME2_UNC0638_peak_116	79	*Tldc2*
chr2	163918616	163918951	H3K9ME2_UNC0638_peak_117	51	*Gm35886*
chr2	164168141	164168545	H3K9ME2_UNC0638_peak_118	27	-

© 2023. Bellver-Sanchis A. et al. Published online at http://www.aginganddisease.org/EN/10.14336/AD.2023.0424-2

chr2	164199756	164200268	H3K9ME2_UNC0638_peak_119	50	*Wfdc15a*
chr2	164804818	164805109	H3K9ME2_UNC0638_peak_120	44	*Zswim3*
chr2	164967912	164968491	H3K9ME2_UNC0638_peak_121	38	*Slc12a5*
chr2	174110346	174110681	H3K9ME2_UNC0638_peak_122	38	*Npepl1*
chr2	178414411	178414806	H3K9ME2_UNC0638_peak_123	33	*Fam217b*
chr2	180892914	180893361	H3K9ME2_UNC0638_peak_124	24	*Mir124a-3*
chr3	34561765	34562056	H3K9ME2_UNC0638_peak_125	36	*Sox2ot*
chr3	8510144	8510435	H3K9ME2_UNC0638_peak_126	26	*Stmn2*
chr3	8510900	8511332	H3K9ME2_UNC0638_peak_127	17	*Stmn2*
chr3	37063597	37063902	H3K9ME2_UNC0638_peak_128	27	-
chr3	37348466	37348874	H3K9ME2_UNC0638_peak_129	50	*Fgf2*
chr3	59005789	59006358	H3K9ME2_UNC0638_peak_130	27	*Med12l*
chr3	62603656	62603992	H3K9ME2_UNC0638_peak_131	25	-
chr3	105452355	105452766	H3K9ME2_UNC0638_peak_132	28	-
chr3	145987948	145988470	H3K9ME2_UNC0638_peak_133	38	*Syde2*
chr3	146220815	146221383	H3K9ME2_UNC0638_peak_134	38	*Lpar3*
chr3	18054677	18055448	H3K9ME2_UNC0638_peak_135	35	*Bhlhe22*
chr3	27371835	27372505	H3K9ME2_UNC0638_peak_136	31	*Ghsr*
chr3	31309800	31310370	H3K9ME2_UNC0638_peak_137	23	-
chr3	35932605	35932896	H3K9ME2_UNC0638_peak_138	22	-
chr3	39277391	39278016	H3K9ME2_UNC0638_peak_139	32	*Gm42214*
chr3	41555841	41556362	H3K9ME2_UNC0638_peak_140	90	*Jade1*
chr3	55181750	55182041	H3K9ME2_UNC0638_peak_141	32	*Sohlh2*
chr3	68869807	68870580	H3K9ME2_UNC0638_peak_142	98	*1110032F04Rik*
chr3	82905219	82905668	H3K9ME2_UNC0638_peak_143	26	*Rbm46os*
chr3	85340864	85341219	H3K9ME2_UNC0638_peak_144	22	*Gm31210*
chr3	87412031	87412382	H3K9ME2_UNC0638_peak_145	28	*Gm40084*
chr3	89773327	89773771	H3K9ME2_UNC0638_peak_146	32	*Ube2q1*
chr3	89830948	89831701	H3K9ME2_UNC0638_peak_147	37	*She*
chr3	94363489	94364279	H3K9ME2_UNC0638_peak_148	38	*C2cd4d*
chr3	94412767	94413058	H3K9ME2_UNC0638_peak_149	22	-
chr3	100472586	100472968	H3K9ME2_UNC0638_peak_150	26	*Tent5c*
chr3	102011590	102013060	H3K9ME2_UNC0638_peak_151	55	*Nhlh2*
chr3	103576358	103576832	H3K9ME2_UNC0638_peak_152	16	*Syt6*
chr3	118434492	118434912	H3K9ME2_UNC0638_peak_153	61	*Mir137*
chr3	121590881	121591428	H3K9ME2_UNC0638_peak_154	64	*Gm33708*
chr3	121952957	121953574	H3K9ME2_UNC0638_peak_155	37	*Arhgap29*
chr3	121953739	121954030	H3K9ME2_UNC0638_peak_156	27	*Arhgap29*
chr4	9269371	9269680	H3K9ME2_UNC0638_peak_157	30	*Clvs1*
chr4	17853311	17853817	H3K9ME2_UNC0638_peak_158	50	*Mmp16*

© 2023. Bellver-Sanchis A. et al. Published online at http://www.aginganddisease.org/EN/10.14336/AD.2023.0424-2

chr4	73790885	73791176	H3K9ME2_UNC0638_peak_159	22	*Gm11240*
chr4	86276617	86276912	H3K9ME2_UNC0638_peak_160	44	-
chr4	114405860	114406219	H3K9ME2_UNC0638_peak_161	71	-
chr4	128654445	128655013	H3K9ME2_UNC0638_peak_162	22	*Phc2*
chr4	128688272	128688655	H3K9ME2_UNC0638_peak_163	22	*Phc2*
chr4	134073036	134073327	H3K9ME2_UNC0638_peak_164	26	*Crybg2*
chr4	136835514	136835808	H3K9ME2_UNC0638_peak_165	35	-
chr4	136836292	136836583	H3K9ME2_UNC0638_peak_166	20	-
chr4	9917002	9917391	H3K9ME2_UNC0638_peak_167	22	*4930448K20Rik*
chr4	11191092	11191383	H3K9ME2_UNC0638_peak_168	57	*Ccne2*
chr4	11385547	11385884	H3K9ME2_UNC0638_peak_169	17	*Gm35631*
chr4	21678809	21680027	H3K9ME2_UNC0638_peak_170	38	*Prdm13*
chr4	41194770	41195197	H3K9ME2_UNC0638_peak_171	44	-
chr4	42916900	42917191	H3K9ME2_UNC0638_peak_172	36	-
chr4	42917368	42917659	H3K9ME2_UNC0638_peak_173	34	-
chr4	87100836	87101307	H3K9ME2_UNC0638_peak_174	26	-
chr4	91371585	91372221	H3K9ME2_UNC0638_peak_175	38	*Mir6402*
chr4	99656561	99657243	H3K9ME2_UNC0638_peak_176	26	*Foxd3*
chr4	101068884	101069239	H3K9ME2_UNC0638_peak_177	50	*Raver2*
chr4	108217335	108217868	H3K9ME2_UNC0638_peak_178	32	-
chr4	114907608	114908038	H3K9ME2_UNC0638_peak_179	22	*Foxd2*
chr4	114925044	114925530	H3K9ME2_UNC0638_peak_180	22	*Foxe3*
chr4	114925685	114926329	H3K9ME2_UNC0638_peak_181	38	*Foxe3*
chr4	119072889	119073309	H3K9ME2_UNC0638_peak_182	44	*Gm35510*
chr4	120854625	120855205	H3K9ME2_UNC0638_peak_183	38	*Rims3*
chr4	122996554	122997014	H3K9ME2_UNC0638_peak_184	48	*Mycl*
chr4	123104957	123105424	H3K9ME2_UNC0638_peak_185	27	*Bmp8b*
chr4	124658377	124658947	H3K9ME2_UNC0638_peak_186	27	*Pou3f1*
chr4	129239044	129239347	H3K9ME2_UNC0638_peak_187	38	-
chr5	117413730	117414317	H3K9ME2_UNC0638_peak_188	44	*Ksr2*
chr5	9266000	9266621	H3K9ME2_UNC0638_peak_189	43	-
chr5	9381168	9381459	H3K9ME2_UNC0638_peak_190	32	-
chr5	14514991	14515450	H3K9ME2_UNC0638_peak_191	38	-
chr5	19226254	19226885	H3K9ME2_UNC0638_peak_192	61	-
chr5	22746374	22746893	H3K9ME2_UNC0638_peak_193	47	-
chr5	36490757	36491339	H3K9ME2_UNC0638_peak_194	22	*Tbc1d14*
chr5	63648773	63649519	H3K9ME2_UNC0638_peak_195	32	*Nwd2*
chr5	63650077	63650608	H3K9ME2_UNC0638_peak_196	36	*Nwd2*
chr5	66745534	66746395	H3K9ME2_UNC0638_peak_197	51	*Limch1*
chr5	71700358	71701151	H3K9ME2_UNC0638_peak_198	58	*Gabrb1*

SUPPLEMENTARY DATA

chr5	123076207	123076540	H3K9ME2_UNC0638_peak_199	22	*Tmem120b*
chr5	125532722	125533117	H3K9ME2_UNC0638_peak_200	28	*Tmem132b*
chr5	130384371	130384822	H3K9ME2_UNC0638_peak_201	28	*Caln1*
chr5	147957165	147957486	H3K9ME2_UNC0638_peak_202	42	*Mtus2*
chr5	15470190	15470564	H3K9ME2_UNC0638_peak_203	48	-
chr5	15512179	15512639	H3K9ME2_UNC0638_peak_204	230	*Gm21847*
chr5	15678922	15679504	H3K9ME2_UNC0638_peak_205	43	*Speer4cos*
chr5	22358375	22358685	H3K9ME2_UNC0638_peak_206	30	*Gm42223*
chr5	24473986	24474824	H3K9ME2_UNC0638_peak_207	42	*Agap3*
chr5	24730098	24730683	H3K9ME2_UNC0638_peak_208	62	*Gm42231*
chr5	26006909	26007620	H3K9ME2_UNC0638_peak_209	56	-
chr5	28071231	28071636	H3K9ME2_UNC0638_peak_210	27	*Insig1*
chr5	30587843	30589077	H3K9ME2_UNC0638_peak_211	50	*Kcnk3*
chr5	36967255	36967758	H3K9ME2_UNC0638_peak_212	32	*Wfs1*
chr5	45639315	45639750	H3K9ME2_UNC0638_peak_213	54	*9630001P10Rik*
chr5	64803469	64803773	H3K9ME2_UNC0638_peak_214	38	-
chr5	67391205	67391559	H3K9ME2_UNC0638_peak_215	27	*Bend4*
chr5	72564033	72564607	H3K9ME2_UNC0638_peak_216	22	*Gm34144*
chr5	75075840	75076278	H3K9ME2_UNC0638_peak_217	43	*Gsx2*
chr5	87850743	87851385	H3K9ME2_UNC0638_peak_218	27	*Gm7337*
chr5	98854472	98855174	H3K9ME2_UNC0638_peak_219	36	*Bmp3*
chr5	100728097	100728477	H3K9ME2_UNC0638_peak_220	24	*Gm36619*
chr5	110544851	110545173	H3K9ME2_UNC0638_peak_221	32	*Galnt9*
chr5	110841155	110841446	H3K9ME2_UNC0638_peak_222	44	-
chr5	111558652	111558953	H3K9ME2_UNC0638_peak_223	27	*Gm36274*
chr5	112342974	112343312	H3K9ME2_UNC0638_peak_224	50	*Hps4*
chr5	114130626	114131025	H3K9ME2_UNC0638_peak_225	88	*Ung*
chr5	122092570	122092911	H3K9ME2_UNC0638_peak_226	38	-
chr5	137179118	137179650	H3K9ME2_UNC0638_peak_227	57	-
chr5	139908016	139909036	H3K9ME2_UNC0638_peak_228	84	*Elfn1*
chr5	140607450	140607758	H3K9ME2_UNC0638_peak_229	17	*Lfng*
chr5	142701840	142702724	H3K9ME2_UNC0638_peak_230	96	*Slc29a4*
chr5	142960854	142961174	H3K9ME2_UNC0638_peak_231	37	*Fscn1*
chr5	145231828	145232119	H3K9ME2_UNC0638_peak_232	27	*Zfp655*
chr5	146492781	146493077	H3K9ME2_UNC0638_peak_233	57	-
chr5	150952676	150953104	H3K9ME2_UNC0638_peak_234	34	*Kl*
chr6	125710564	125711128	H3K9ME2_UNC0638_peak_235	31	-
chr6	29347863	29348450	H3K9ME2_UNC0638_peak_236	72	*Calu*
chr6	31399011	31399380	H3K9ME2_UNC0638_peak_237	72	*Mkln1*
chr6	45059969	45060299	H3K9ME2_UNC0638_peak_238	34	*Cntnap2*

© 2023. Bellver-Sanchis A. et al. Published online at http://www.aginganddisease.org/EN/10.14336/AD.2023.0424-2

chr6	48137855	48138234	H3K9ME2_UNC0638_peak_239	26	-
chr6	61180566	61181124	H3K9ME2_UNC0638_peak_240	37	*Ccser1*
chr6	104491346	104491652	H3K9ME2_UNC0638_peak_241	27	*Cntn6*
chr6	110645671	110646145	H3K9ME2_UNC0638_peak_242	36	*Grm7*
chr6	114282731	114283114	H3K9ME2_UNC0638_peak_243	53	*Slc6a1*
chr6	127887683	127888092	H3K9ME2_UNC0638_peak_244	43	-
chr6	145746148	145746578	H3K9ME2_UNC0638_peak_245	50	-
chr6	3764142	3764680	H3K9ME2_UNC0638_peak_246	41	*Gm33576*
chr6	5725688	5726571	H3K9ME2_UNC0638_peak_247	46	*Dync1i1*
chr6	8949438	8949919	H3K9ME2_UNC0638_peak_248	27	*Nxph1*
chr6	14901333	14901782	H3K9ME2_UNC0638_peak_249	36	*Foxp2*
chr6	16525711	16526048	H3K9ME2_UNC0638_peak_250	39	*Gm36503*
chr6	25931131	25931727	H3K9ME2_UNC0638_peak_251	21	*Gm20756*
chr6	30048114	30048412	H3K9ME2_UNC0638_peak_252	32	*Nrf1*
chr6	37331787	37332452	H3K9ME2_UNC0638_peak_253	38	*Creb3l2*
chr6	37871087	37871472	H3K9ME2_UNC0638_peak_254	32	*Trim24*
chr6	38662678	38663527	H3K9ME2_UNC0638_peak_255	44	*Clec2l*
chr6	65381259	65381659	H3K9ME2_UNC0638_peak_256	48	*Qrfprl*
chr6	65778732	65779113	H3K9ME2_UNC0638_peak_257	37	*Prdm5*
chr6	82946263	82947281	H3K9ME2_UNC0638_peak_258	65	*M1ap*
chr6	90309220	90309577	H3K9ME2_UNC0638_peak_259	20	*Chst13*
chr6	91684033	91684391	H3K9ME2_UNC0638_peak_260	32	-
chr6	97616897	97617808	H3K9ME2_UNC0638_peak_261	43	*Gm32247*
chr6	100704602	100705171	H3K9ME2_UNC0638_peak_262	33	*Gxylt2*
chr6	113748876	113749283	H3K9ME2_UNC0638_peak_263	38	*Atp2b2*
chr6	114131114	114131762	H3K9ME2_UNC0638_peak_264	32	*Slc6a11*
chr6	114397637	114398279	H3K9ME2_UNC0638_peak_265	39	*Hrh1*
chr6	115134241	115135711	H3K9ME2_UNC0638_peak_266	131	*Syn2*
chr6	115601269	115601609	H3K9ME2_UNC0638_peak_267	50	*Mkrn2*
chr6	120666218	120667220	H3K9ME2_UNC0638_peak_268	32	*Cecr2*
chr6	125009207	125009541	H3K9ME2_UNC0638_peak_269	64	*4930557K07Rik*
chr6	129459138	129459598	H3K9ME2_UNC0638_peak_270	43	-
chr6	134444870	134445161	H3K9ME2_UNC0638_peak_271	38	*Lrp6*
chr6	141249822	141250172	H3K9ME2_UNC0638_peak_272	32	*Pde3a*
chr6	146888038	146888367	H3K9ME2_UNC0638_peak_273	32	*Ppfibp1*
chr6	148047167	148047772	H3K9ME2_UNC0638_peak_274	37	-
chr6	4003872	4004498	H3K9ME2_UNC0638_peak_275	33	*Gng11*
chr6	4505101	4505665	H3K9ME2_UNC0638_peak_276	82	*Col1a2*
chr6	4600865	4601665	H3K9ME2_UNC0638_peak_277	44	-
chr6	7554906	7555396	H3K9ME2_UNC0638_peak_278	46	*Tac1*

© 2023. Bellver-Sanchis A. et al. Published online at http://www.aginganddisease.org/EN/10.14336/AD.2023.0424-2

SUPPLEMENTARY DATA

chr6	8331870	8332343	H3K9ME2_UNC0638_peak_279	50	*Gm46952*
chr6	10893600	10894003	H3K9ME2_UNC0638_peak_280	41	-
chr6	17065138	17065504	H3K9ME2_UNC0638_peak_281	65	-
chr6	22288382	22288703	H3K9ME2_UNC0638_peak_282	26	*Wnt16*
chr6	26740391	26740939	H3K9ME2_UNC0638_peak_283	57	-
chr6	29031846	29032151	H3K9ME2_UNC0638_peak_284	42	*Lnclep*
chr6	29060154	29060496	H3K9ME2_UNC0638_peak_285	40	-
chr6	30957378	30958411	H3K9ME2_UNC0638_peak_286	79	*Klf14*
chr6	31010145	31010764	H3K9ME2_UNC0638_peak_287	36	*Gm40359*
chr6	35176673	35176964	H3K9ME2_UNC0638_peak_288	32	*Nup205*
chr6	38551437	38551922	H3K9ME2_UNC0638_peak_289	38	*Luc7l2*
chr6	39063330	39063944	H3K9ME2_UNC0638_peak_290	17	*1700025N23Rik*
chr6	39118520	39118910	H3K9ME2_UNC0638_peak_291	44	*4930599N23Rik*
chr6	47121034	47121462	H3K9ME2_UNC0638_peak_292	50	-
chr6	47650566	47651559	H3K9ME2_UNC0638_peak_293	58	-
chr6	47943025	47943522	H3K9ME2_UNC0638_peak_294	21	*Zfp783*
chr6	48024618	48024932	H3K9ME2_UNC0638_peak_295	25	*Zfp777*
chr6	48402813	48403104	H3K9ME2_UNC0638_peak_296	25	*Krba1*
chr6	48765307	48765819	H3K9ME2_UNC0638_peak_297	23	*Gimap3*
chr6	49822185	49822690	H3K9ME2_UNC0638_peak_298	41	*Npy*
chr6	50110304	50110618	H3K9ME2_UNC0638_peak_299	32	*Pals2*
chr6	51432567	51433488	H3K9ME2_UNC0638_peak_300	46	*Nfe2l3*
chr6	51469272	51469698	H3K9ME2_UNC0638_peak_301	22	-
chr6	52190871	52191541	H3K9ME2_UNC0638_peak_302	81	*Hoxa4*
chr6	52246213	52246556	H3K9ME2_UNC0638_peak_303	31	*Hoxa11os*
chr6	52259746	52260296	H3K9ME2_UNC0638_peak_304	37	*Hoxa13*
chr6	53845021	53845361	H3K9ME2_UNC0638_peak_305	31	*Gm16499*
chr6	54429828	54430195	H3K9ME2_UNC0638_peak_306	71	*Wipf3*
chr6	55451783	55452384	H3K9ME2_UNC0638_peak_307	33	*Adcyap1r1*
chr6	55676382	55677250	H3K9ME2_UNC0638_peak_308	53	*Neurod6*
chr6	57022911	57023327	H3K9ME2_UNC0638_peak_309	18	*Vmn1r7*
chr6	57359927	57360284	H3K9ME2_UNC0638_peak_310	24	*Vmn1r17*
chr6	58906705	58907415	H3K9ME2_UNC0638_peak_311	31	*Nap1l5*
chr6	64729706	64730169	H3K9ME2_UNC0638_peak_312	57	*Atoh1*
chr6	67267311	67267602	H3K9ME2_UNC0638_peak_313	38	*Serbp1*
chr6	70956312	70957287	H3K9ME2_UNC0638_peak_314	97	*Foxi3*
chr6	71456720	71457437	H3K9ME2_UNC0638_peak_315	47	*Gm30582*
chr6	71707600	71707961	H3K9ME2_UNC0638_peak_316	51	*Reep1*
chr6	71708531	71708822	H3K9ME2_UNC0638_peak_317	22	*Reep1*
chr6	72439582	72439879	H3K9ME2_UNC0638_peak_318	32	*Particl*

© 2023. Bellver-Sanchis A. et al. Published online at http://www.aginganddisease.org/EN/10.14336/AD.2023.0424-2

chr6	72958365	72958686	H3K9ME2_UNC0638_peak_319	32	*Tmsb10*
chr6	81900013	81900365	H3K9ME2_UNC0638_peak_320	23	*1700009C05Rik*
chr6	81932513	81932841	H3K9ME2_UNC0638_peak_321	32	*Gcfc2*
chr6	83033453	83033800	H3K9ME2_UNC0638_peak_322	38	*Loxl3*
chr6	83069629	83069927	H3K9ME2_UNC0638_peak_323	22	*Tlx2*
chr6	83077801	83078174	H3K9ME2_UNC0638_peak_324	44	*Pcgf1*
chr6	85511889	85512289	H3K9ME2_UNC0638_peak_325	47	*Egr4*
chr6	86526076	86526836	H3K9ME2_UNC0638_peak_326	35	*1600020E01Rik*
chr6	87638341	87638656	H3K9ME2_UNC0638_peak_327	43	*Gm46964*
chr6	87730732	87731097	H3K9ME2_UNC0638_peak_328	35	-
chr6	87980462	87981850	H3K9ME2_UNC0638_peak_329	41	*H1f10*
chr6	88142147	88142705	H3K9ME2_UNC0638_peak_330	61	*Gm38708*
chr6	88166127	88166758	H3K9ME2_UNC0638_peak_331	29	*Gm38708*
chr6	90594291	90595210	H3K9ME2_UNC0638_peak_332	31	-
chr6	100467952	100468343	H3K9ME2_UNC0638_peak_333	31	*Gm38868*
chr6	107529641	107530353	H3K9ME2_UNC0638_peak_334	71	*Lrrn1*
chr6	108828857	108829319	H3K9ME2_UNC0638_peak_335	27	*Edem1*
chr6	113426108	113426575	H3K9ME2_UNC0638_peak_336	32	-
chr6	113494232	113494856	H3K9ME2_UNC0638_peak_337	50	*Prrt3*
chr6	114094350	114094907	H3K9ME2_UNC0638_peak_338	37	*Gm36023*
chr6	115305795	115306345	H3K9ME2_UNC0638_peak_339	32	*Gm38877*
chr6	115526742	115527110	H3K9ME2_UNC0638_peak_340	17	*Gm36355*
chr6	116580122	116580585	H3K9ME2_UNC0638_peak_341	44	*Olfr215*
chr6	117843270	117843725	H3K9ME2_UNC0638_peak_342	57	*Zfp637*
chr6	119175120	119175833	H3K9ME2_UNC0638_peak_343	75	*Dcp1b*
chr6	119479499	119479797	H3K9ME2_UNC0638_peak_344	32	*Fbxl14*
chr6	122873427	122874129	H3K9ME2_UNC0638_peak_345	57	*Necap1*
chr6	124303330	124303629	H3K9ME2_UNC0638_peak_346	23	*Cd163*
chr6	124531016	124531307	H3K9ME2_UNC0638_peak_347	31	*C1s1*
chr6	124625539	124625830	H3K9ME2_UNC0638_peak_348	22	*C1s2*
chr6	124829824	124830541	H3K9ME2_UNC0638_peak_349	27	*Cdca3*
chr6	126939635	126939926	H3K9ME2_UNC0638_peak_350	50	*D6Wsu163e*
chr6	132114656	132115168	H3K9ME2_UNC0638_peak_351	57	-
chr6	132207781	132208078	H3K9ME2_UNC0638_peak_352	33	*Prb1*
chr6	132312250	132312699	H3K9ME2_UNC0638_peak_353	89	*Prpmp5*
chr6	132361404	132362093	H3K9ME2_UNC0638_peak_354	45	*Gm8882*
chr6	132439390	132439874	H3K9ME2_UNC0638_peak_355	86	*Gm5154*
chr6	132710153	132710542	H3K9ME2_UNC0638_peak_356	45	*Tas2r122*
chr6	134397535	134398151	H3K9ME2_UNC0638_peak_357	29	*Bcl2l14*
chr6	135309804	135310096	H3K9ME2_UNC0638_peak_358	26	*Pbp2*

© 2023. Bellver-Sanchis A. et al. Published online at http://www.aginganddisease.org/EN/10.14336/AD.2023.0424-2

chr6	136326955	136327253	H3K9ME2_UNC0638_peak_359	27	*Eif4a3l1*
chr6	136804096	136804389	H3K9ME2_UNC0638_peak_360	38	*H4f16*
chr6	142387009	142387300	H3K9ME2_UNC0638_peak_361	38	-
chr6	143683260	143683555	H3K9ME2_UNC0638_peak_362	33	-
chr6	146725039	146725572	H3K9ME2_UNC0638_peak_363	106	*Stk38l*
chr7	92060984	92061292	H3K9ME2_UNC0638_peak_364	44	*Dlg2*
chr7	57590571	57591187	H3K9ME2_UNC0638_peak_365	22	*Gabrb3*
chr7	87584140	87584624	H3K9ME2_UNC0638_peak_366	47	*Grm5*
chr7	122670857	122671227	H3K9ME2_UNC0638_peak_367	23	*Cacng3*
chr7	4844109	4844976	H3K9ME2_UNC0638_peak_368	85	*Isoc2b*
chr7	10088275	10089170	H3K9ME2_UNC0638_peak_369	27	*Vmn2r51*
chr7	19118080	19118754	H3K9ME2_UNC0638_peak_370	57	*Fbxo46*
chr7	25009963	25010460	H3K9ME2_UNC0638_peak_371	29	*Grik5*
chr7	29064410	29064781	H3K9ME2_UNC0638_peak_372	30	-
chr7	29065849	29066157	H3K9ME2_UNC0638_peak_373	40	-
chr7	46396362	46397093	H3K9ME2_UNC0638_peak_374	39	*Kcnc1*
chr7	49908958	49909249	H3K9ME2_UNC0638_peak_375	46	*Slc6a5*
chr7	54835607	54836122	H3K9ME2_UNC0638_peak_376	35	*Luzp2*
chr7	57386585	57386927	H3K9ME2_UNC0638_peak_377	36	*Gm9962*
chr7	63099224	63099697	H3K9ME2_UNC0638_peak_378	37	*Chrna7*
chr7	63444808	63445159	H3K9ME2_UNC0638_peak_379	35	*Otud7a*
chr7	81213098	81213601	H3K9ME2_UNC0638_peak_380	42	*Pde8a*
chr7	84529026	84529317	H3K9ME2_UNC0638_peak_381	50	-
chr7	86737276	86737567	H3K9ME2_UNC0638_peak_382	27	*Folh1*
chr7	97580120	97580567	H3K9ME2_UNC0638_peak_383	27	-
chr7	101577333	101577837	H3K9ME2_UNC0638_peak_384	49	*Art2b*
chr7	102209624	102210186	H3K9ME2_UNC0638_peak_385	44	*Pgap2*
chr7	105481832	105482322	H3K9ME2_UNC0638_peak_386	32	*Gm36847*
chr7	112679045	112679871	H3K9ME2_UNC0638_peak_387	38	*Tead1*
chr7	113764098	113764538	H3K9ME2_UNC0638_peak_388	22	*Spon1*
chr7	120420885	120421334	H3K9ME2_UNC0638_peak_389	49	*Abca16*
chr7	121864855	121865182	H3K9ME2_UNC0638_peak_390	30	*Scnn1b*
chr7	127512284	127512641	H3K9ME2_UNC0638_peak_391	38	*Srcap*
chr7	128842827	128843212	H3K9ME2_UNC0638_peak_392	47	*Gm39093*
chr7	139977797	139978298	H3K9ME2_UNC0638_peak_393	45	*Adam8*
chr7	141950166	141950645	H3K9ME2_UNC0638_peak_394	59	*Brsk2*
chr7	142967963	142968362	H3K9ME2_UNC0638_peak_395	43	-
chr7	143106948	143107768	H3K9ME2_UNC0638_peak_396	64	*Kcnq1*
chr7	4993604	4994479	H3K9ME2_UNC0638_peak_397	34	*Zfp579*
chr7	5146571	5147163	H3K9ME2_UNC0638_peak_398	17	*Vmn1r55*

© 2023. Bellver-Sanchis A. et al. Published online at http://www.aginganddisease.org/EN/10.14336/AD.2023.0424-2

SUPPLEMENTARY DATA

chr7	6201130	6201612	H3K9ME2_UNC0638_peak_399	27	-
chr7	6440036	6440368	H3K9ME2_UNC0638_peak_400	31	*Olfr1344*
chr7	6488669	6489089	H3K9ME2_UNC0638_peak_401	26	*Olfr1347*
chr7	7300006	7300428	H3K9ME2_UNC0638_peak_402	27	*Mir5620*
chr7	8368787	8369078	H3K9ME2_UNC0638_peak_403	44	*Vmn2r44*
chr7	10578115	10578406	H3K9ME2_UNC0638_peak_404	26	*Vmn1r69*
chr7	12258184	12258490	H3K9ME2_UNC0638_peak_405	31	*Vmn1r81*
chr7	13043765	13044361	H3K9ME2_UNC0638_peak_406	26	*Mzf1*
chr7	16061286	16062005	H3K9ME2_UNC0638_peak_407	76	*Zfp541*
chr7	16816285	16816648	H3K9ME2_UNC0638_peak_408	45	*Strn4*
chr7	18319334	18319836	H3K9ME2_UNC0638_peak_409	37	-
chr7	18674185	18674476	H3K9ME2_UNC0638_peak_410	16	*Psg20*
chr7	18674754	18675139	H3K9ME2_UNC0638_peak_411	32	*Psg20*
chr7	19507218	19507644	H3K9ME2_UNC0638_peak_412	17	*Bloc1s3*
chr7	23384026	23384445	H3K9ME2_UNC0638_peak_413	23	*Nlrp5*
chr7	24432150	24432592	H3K9ME2_UNC0638_peak_414	49	-
chr7	24585532	24586416	H3K9ME2_UNC0638_peak_415	68	*Zfp575*
chr7	24586622	24586954	H3K9ME2_UNC0638_peak_416	19	*Ethe1*
chr7	24869999	24870290	H3K9ME2_UNC0638_peak_417	50	*Dmrtc2*
chr7	25282436	25282735	H3K9ME2_UNC0638_peak_418	25	*Cic*
chr7	28283199	28284063	H3K9ME2_UNC0638_peak_419	283	*Selenov*
chr7	28808807	28809100	H3K9ME2_UNC0638_peak_420	53	*Hnrnpl*
chr7	29773976	29774267	H3K9ME2_UNC0638_peak_421	28	*Mir1964*
chr7	30854931	30855235	H3K9ME2_UNC0638_peak_422	32	*Ffar3*
chr7	30882402	30882905	H3K9ME2_UNC0638_peak_423	49	*D7Ertd128e*
chr7	34522000	34522313	H3K9ME2_UNC0638_peak_424	25	-
chr7	35185143	35185754	H3K9ME2_UNC0638_peak_425	32	-
chr7	35185936	35186791	H3K9ME2_UNC0638_peak_426	59	-
chr7	43768952	43769346	H3K9ME2_UNC0638_peak_427	73	*Klk12*
chr7	44123035	44123326	H3K9ME2_UNC0638_peak_428	21	*Klk1b16*
chr7	45525852	45526190	H3K9ME2_UNC0638_peak_429	32	*Plekha4*
chr7	45628350	45629057	H3K9ME2_UNC0638_peak_430	63	*Rasip1*
chr7	45832536	45834160	H3K9ME2_UNC0638_peak_431	67	-
chr7	46796060	46796409	H3K9ME2_UNC0638_peak_432	22	*Gtf2h1*
chr7	47588372	47589603	H3K9ME2_UNC0638_peak_433	178	*Mrgpra3*
chr7	47981011	47981674	H3K9ME2_UNC0638_peak_434	32	*Mrgpra4*
chr7	59332800	59333238	H3K9ME2_UNC0638_peak_435	22	-
chr7	60155480	60155797	H3K9ME2_UNC0638_peak_436	50	*Gm7367*
chr7	62348882	62349173	H3K9ME2_UNC0638_peak_437	23	*Ndn*
chr7	62377574	62378019	H3K9ME2_UNC0638_peak_438	35	*Magel2*

© 2023. Bellver-Sanchis A. et al. Published online at http://www.aginganddisease.org/EN/10.14336/AD.2023.0424-2

chr7	62378360	62378835	H3K9ME2_UNC0638_peak_439	35	*Magel2*
chr7	66733705	66734331	H3K9ME2_UNC0638_peak_440	22	*Cers3*
chr7	73740203	73741049	H3K9ME2_UNC0638_peak_441	75	*Fam174b*
chr7	79515404	79515849	H3K9ME2_UNC0638_peak_442	44	*Mir9-3hg*
chr7	81933751	81934362	H3K9ME2_UNC0638_peak_443	46	-
chr7	84409490	84410423	H3K9ME2_UNC0638_peak_444	50	*Gm39045*
chr7	91013574	91014410	H3K9ME2_UNC0638_peak_445	57	*Gm39048*
chr7	93079718	93080031	H3K9ME2_UNC0638_peak_446	23	*Fam181b*
chr7	93080435	93081053	H3K9ME2_UNC0638_peak_447	28	*Fam181b*
chr7	99625368	99626962	H3K9ME2_UNC0638_peak_448	115	*Tpbgl*
chr7	101818093	101818682	H3K9ME2_UNC0638_peak_449	33	*Phox2a*
chr7	103938021	103938331	H3K9ME2_UNC0638_peak_450	34	*Olfr632*
chr7	104012427	104012765	H3K9ME2_UNC0638_peak_451	17	*Olfr639*
chr7	104129213	104129842	H3K9ME2_UNC0638_peak_452	20	*Ubqln5*
chr7	105425814	105426227	H3K9ME2_UNC0638_peak_453	22	*Cckbr*
chr7	106752665	106752980	H3K9ME2_UNC0638_peak_454	25	*Olfr698*
chr7	106886166	106886883	H3K9ME2_UNC0638_peak_455	27	*Olfr706*
chr7	109010597	109011430	H3K9ME2_UNC0638_peak_456	69	*Tub*
chr7	109438702	109439290	H3K9ME2_UNC0638_peak_457	61	-
chr7	112186679	112187229	H3K9ME2_UNC0638_peak_458	55	*Gm39071*
chr7	112953683	112954814	H3K9ME2_UNC0638_peak_459	67	*Rassf10*
chr7	112955170	112955587	H3K9ME2_UNC0638_peak_460	53	*Rassf10*
chr7	120917906	120918197	H3K9ME2_UNC0638_peak_461	17	-
chr7	126583783	126584096	H3K9ME2_UNC0638_peak_462	44	*Apobr*
chr7	127026024	127026544	H3K9ME2_UNC0638_peak_463	21	*Kif22*
chr7	127287507	127287831	H3K9ME2_UNC0638_peak_464	17	-
chr7	127967253	127967641	H3K9ME2_UNC0638_peak_465	37	-
chr7	131543268	131544191	H3K9ME2_UNC0638_peak_466	36	*Hmx3*
chr7	132596921	132597783	H3K9ME2_UNC0638_peak_467	31	*Nkx1-2*
chr7	135410178	135410773	H3K9ME2_UNC0638_peak_468	32	*Foxi2*
chr7	135853334	135853755	H3K9ME2_UNC0638_peak_469	22	-
chr7	136352166	136352457	H3K9ME2_UNC0638_peak_470	19	-
chr7	139086053	139086990	H3K9ME2_UNC0638_peak_471	38	*Dpysl4*
chr7	140070223	140070604	H3K9ME2_UNC0638_peak_472	17	*Caly*
chr7	141061364	141061655	H3K9ME2_UNC0638_peak_473	27	*B4galnt4*
chr7	142094662	142095555	H3K9ME2_UNC0638_peak_474	38	-
chr7	143459498	143459998	H3K9ME2_UNC0638_peak_475	53	*Cdkn1c*
chr7	144861242	144862171	H3K9ME2_UNC0638_peak_476	50	*Fgf4*
chr8	33516437	33516789	H3K9ME2_UNC0638_peak_477	38	*Tex15*
chr8	33516968	33517426	H3K9ME2_UNC0638_peak_478	22	*Tex15*

© 2023. Bellver-Sanchis A. et al. Published online at http://www.aginganddisease.org/EN/10.14336/AD.2023.0424-2

SUPPLEMENTARY DATA

chr8	10153761	10154236	H3K9ME2_UNC0638_peak_479	41	*Myo16*
chr8	14096107	14096767	H3K9ME2_UNC0638_peak_480	35	*Dlgap2*
chr8	37564444	37564739	H3K9ME2_UNC0638_peak_481	32	*Sgcz*
chr8	40862364	40862655	H3K9ME2_UNC0638_peak_482	44	*Slc7a2*
chr8	11009054	11009494	H3K9ME2_UNC0638_peak_483	50	*9530052E02Rik*
chr8	36248404	36248923	H3K9ME2_UNC0638_peak_484	43	*Gm35712*
chr8	38660413	38660787	H3K9ME2_UNC0638_peak_485	38	*Gm40493*
chr8	62951457	62952021	H3K9ME2_UNC0638_peak_486	38	*Spock3*
chr8	77723807	77724262	H3K9ME2_UNC0638_peak_487	31	*LOC108167319*
chr8	90248383	90248992	H3K9ME2_UNC0638_peak_488	32	*Tox3*
chr8	95677551	95677988	H3K9ME2_UNC0638_peak_489	45	-
chr8	117498434	117498729	H3K9ME2_UNC0638_peak_490	28	*Plcg2*
chr8	125054058	125054639	H3K9ME2_UNC0638_peak_491	36	*Disc1*
chr8	3089289	3089894	H3K9ME2_UNC0638_peak_492	44	*Gm30220*
chr8	19210000	19210297	H3K9ME2_UNC0638_peak_493	50	*Defa-ps12*
chr8	27227725	27228016	H3K9ME2_UNC0638_peak_494	17	-
chr8	27295310	27295875	H3K9ME2_UNC0638_peak_495	29	*Gm9731*
chr8	31089487	31089834	H3K9ME2_UNC0638_peak_496	31	*Dusp26*
chr8	55347211	55347597	H3K9ME2_UNC0638_peak_497	106	-
chr8	55347761	55348298	H3K9ME2_UNC0638_peak_498	176	-
chr8	66511679	66511970	H3K9ME2_UNC0638_peak_499	29	*Tktl2*
chr8	69902132	69902771	H3K9ME2_UNC0638_peak_500	38	*Tssk6*
chr8	70374696	70375105	H3K9ME2_UNC0638_peak_501	32	*Comp*
chr8	78044688	78045212	H3K9ME2_UNC0638_peak_502	36	*Gm29895*
chr8	83290241	83290545	H3K9ME2_UNC0638_peak_503	29	*Ucp1*
chr8	83389668	83390010	H3K9ME2_UNC0638_peak_504	36	-
chr8	85700696	85701036	H3K9ME2_UNC0638_peak_505	38	*Mir8109*
chr8	88272298	88272714	H3K9ME2_UNC0638_peak_506	32	*Adcy7*
chr8	89042667	89042962	H3K9ME2_UNC0638_peak_507	50	-
chr8	92855361	92855652	H3K9ME2_UNC0638_peak_508	43	*Lpcat2*
chr8	93098761	93099052	H3K9ME2_UNC0638_peak_509	27	*Ces1c*
chr8	95753520	95753929	H3K9ME2_UNC0638_peak_510	32	*Mir7073*
chr8	110101400	110101752	H3K9ME2_UNC0638_peak_511	25	-
chr8	110142434	110142888	H3K9ME2_UNC0638_peak_512	31	*Calb2*
chr8	123041939	123042230	H3K9ME2_UNC0638_peak_513	27	*2810013P06Rik*
chr8	123996726	123997017	H3K9ME2_UNC0638_peak_514	22	-
chr8	129044300	129044616	H3K9ME2_UNC0638_peak_515	21	*Ccdc7a*
chr9	46012759	46013050	H3K9ME2_UNC0638_peak_516	32	*Sik3*
chr9	111118060	111118508	H3K9ME2_UNC0638_peak_517	43	*Lrrfip2*
chr9	3532283	3532834	H3K9ME2_UNC0638_peak_518	24	*Gucy1a2*

© 2023. Bellver-Sanchis A. et al. Published online at http://www.aginganddisease.org/EN/10.14336/AD.2023.0424-2

chr9	3533018	3533453	H3K9ME2_UNC0638_peak_519	27	*Gucy1a2*
chr9	8544084	8544702	H3K9ME2_UNC0638_peak_520	42	*Trpc6*
chr9	8900319	8901440	H3K9ME2_UNC0638_peak_521	44	*Pgr*
chr9	45430648	45431206	H3K9ME2_UNC0638_peak_522	54	*Dscaml1*
chr9	51765248	51765957	H3K9ME2_UNC0638_peak_523	71	*Arhgap20*
chr9	52678958	52679867	H3K9ME2_UNC0638_peak_524	29	*Gm1715*
chr9	58823093	58823508	H3K9ME2_UNC0638_peak_525	17	*Hcn4*
chr9	105774178	105774512	H3K9ME2_UNC0638_peak_526	42	*Col6a6*
chr9	106822011	106822316	H3K9ME2_UNC0638_peak_527	50	*Dcaf1*
chr9	107400305	107400596	H3K9ME2_UNC0638_peak_528	54	*Cacna2d2*
chr9	118606862	118607327	H3K9ME2_UNC0638_peak_529	27	*Itga9*
chr9	4795614	4796420	H3K9ME2_UNC0638_peak_530	59	-
chr9	5156699	5157017	H3K9ME2_UNC0638_peak_531	27	*Gm31429*
chr9	8221655	8222083	H3K9ME2_UNC0638_peak_532	71	*1700128F08Rik*
chr9	8245849	8246199	H3K9ME2_UNC0638_peak_533	20	*Mir1899*
chr9	10904359	10905046	H3K9ME2_UNC0638_peak_534	104	*Gm32710*
chr9	14859956	14860695	H3K9ME2_UNC0638_peak_535	39	*Gpr83*
chr9	19359217	19359824	H3K9ME2_UNC0638_peak_536	38	*Olfr846*
chr9	22188027	22188318	H3K9ME2_UNC0638_peak_537	17	*Zfp872*
chr9	36135335	36135644	H3K9ME2_UNC0638_peak_538	31	*Gm3434*
chr9	39215839	39216186	H3K9ME2_UNC0638_peak_539	37	*Olfr944*
chr9	39548887	39549178	H3K9ME2_UNC0638_peak_540	25	*Olfr958*
chr9	39956144	39956499	H3K9ME2_UNC0638_peak_541	22	-
chr9	40136691	40137136	H3K9ME2_UNC0638_peak_542	37	*Gm35123*
chr9	41890649	41891055	H3K9ME2_UNC0638_peak_543	23	-
chr9	43608144	43608492	H3K9ME2_UNC0638_peak_544	29	*Gm29909*
chr9	46997815	46998317	H3K9ME2_UNC0638_peak_545	19	*Gm4791*
chr9	49340248	49340876	H3K9ME2_UNC0638_peak_546	58	*Drd2*
chr9	54286112	54286620	H3K9ME2_UNC0638_peak_547	25	*Gldn*
chr9	54698944	54699582	H3K9ME2_UNC0638_peak_548	38	*Dnaja4*
chr9	54981400	54981878	H3K9ME2_UNC0638_peak_549	22	*Chrna5*
chr9	55518144	55518435	H3K9ME2_UNC0638_peak_550	22	-
chr9	56796460	56797060	H3K9ME2_UNC0638_peak_551	30	*Gm31586*
chr9	58488186	58488496	H3K9ME2_UNC0638_peak_552	16	*Insyn1*
chr9	58488870	58489245	H3K9ME2_UNC0638_peak_553	25	*Insyn1*
chr9	59577934	59578570	H3K9ME2_UNC0638_peak_554	32	*Celf6*
chr9	60712950	60713576	H3K9ME2_UNC0638_peak_555	39	*Larp6*
chr9	62341161	62341452	H3K9ME2_UNC0638_peak_556	22	*Anp32a*
chr9	62536593	62537119	H3K9ME2_UNC0638_peak_557	60	*Gm39352*
chr9	62980419	62980710	H3K9ME2_UNC0638_peak_558	22	*Gm35574*

© 2023. Bellver-Sanchis A. et al. Published online at http://www.aginganddisease.org/EN/10.14336/AD.2023.0424-2

chr9	65630388	65630927	H3K9ME2_UNC0638_peak_559	27	*Rbpms2*
chr9	73102971	73103489	H3K9ME2_UNC0638_peak_560	29	*Khdc3*
chr9	73103734	73104025	H3K9ME2_UNC0638_peak_561	33	*Khdc3*
chr9	78395885	78396196	H3K9ME2_UNC0638_peak_562	36	*Ddx43*
chr9	85843767	85844536	H3K9ME2_UNC0638_peak_563	47	*Tpbg*
chr9	87015121	87016076	H3K9ME2_UNC0638_peak_564	50	-
chr9	90906202	90906546	H3K9ME2_UNC0638_peak_565	36	*Gm31114*
chr9	91923430	91923721	H3K9ME2_UNC0638_peak_566	17	*Gm31409*
chr9	98902913	98903550	H3K9ME2_UNC0638_peak_567	27	*7420426K07Rik*
chr9	102506099	102506491	H3K9ME2_UNC0638_peak_568	22	*Ky*
chr9	106464903	106465259	H3K9ME2_UNC0638_peak_569	22	*Gpr62*
chr9	106886955	106887246	H3K9ME2_UNC0638_peak_570	26	*Manf*
chr9	108306442	108306733	H3K9ME2_UNC0638_peak_571	27	*Rhoa*
chr9	108826356	108826647	H3K9ME2_UNC0638_peak_572	22	*Celsr3*
chr9	110303926	110304288	H3K9ME2_UNC0638_peak_573	17	*Elp6*
chr9	110532642	110533015	H3K9ME2_UNC0638_peak_574	22	-
chr9	110857629	110858377	H3K9ME2_UNC0638_peak_575	17	-
chr9	111311737	111312265	H3K9ME2_UNC0638_peak_576	95	*Trank1*
chr9	116525829	116526218	H3K9ME2_UNC0638_peak_577	27	*Gm39449*
chr9	118478906	118479591	H3K9ME2_UNC0638_peak_578	57	*Eomes*
chr9	119221865	119222326	H3K9ME2_UNC0638_peak_579	41	*Slc22a13b*
chr9	121777944	121778479	H3K9ME2_UNC0638_peak_580	21	-
chr9	122746927	122747729	H3K9ME2_UNC0638_peak_581	38	*Topaz1*
chr9	123963624	123964131	H3K9ME2_UNC0638_peak_582	17	*Ccr1*
chr9	124439926	124440283	H3K9ME2_UNC0638_peak_583	41	-
chrM	2303	2950	H3K9ME2_UNC0638_peak_584	55	-
chrM	3940	4276	H3K9ME2_UNC0638_peak_585	81	-
chrM	5547	6368	H3K9ME2_UNC0638_peak_586	78	-
chrM	12075	13244	H3K9ME2_UNC0638_peak_587	111	-
chrM	14121	15277	H3K9ME2_UNC0638_peak_588	106	-
chrX	6092126	6092679	H3K9ME2_UNC0638_peak_589	44	-
chrX	6779787	6780078	H3K9ME2_UNC0638_peak_590	17	*Dgkk*
chrX	21714826	21715126	H3K9ME2_UNC0638_peak_591	44	*Slc6a14*
chrX	31243437	31243764	H3K9ME2_UNC0638_peak_592	90	*Gm31556*
chrX	31253984	31254829	H3K9ME2_UNC0638_peak_593	113	*Spin2f*
chrX	33057449	33057846	H3K9ME2_UNC0638_peak_594	62	*Gm2837*
chrX	37571675	37571966	H3K9ME2_UNC0638_peak_595	27	*Rhox2f*
chrX	140061455	140061783	H3K9ME2_UNC0638_peak_596	32	*Dnaaf6b*
chrX	169980503	169980977	H3K9ME2_UNC0638_peak_597	70	*Mid1*
chrY	90742078	90743150	H3K9ME2_UNC0638_peak_598	239	-

SUPPLEMENTARY DATA

chr10	4712592	4712965	H3K9ME2_UNC0638_peak_599	29	*Esr1*
chr10	39369954	39370436	H3K9ME2_UNC0638_peak_600	18	*Fyn*
chr10	40222604	40223082	H3K9ME2_UNC0638_peak_601	44	*Rpf2*
chr10	52690652	52691213	H3K9ME2_UNC0638_peak_602	22	*Slc35f1*
chr10	70599076	70600077	H3K9ME2_UNC0638_peak_603	54	-
chr10	84760470	84760761	H3K9ME2_UNC0638_peak_604	21	*Rfx4*
chr10	107271846	107272447	H3K9ME2_UNC0638_peak_605	25	*Lin7a*
chr10	112271435	112271759	H3K9ME2_UNC0638_peak_606	19	*Kcnc2*
chr10	19850243	19851648	H3K9ME2_UNC0638_peak_607	57	*Slc35d3*
chr10	22184524	22184960	H3K9ME2_UNC0638_peak_608	50	-
chr10	26673837	26674223	H3K9ME2_UNC0638_peak_609	27	*Gm8709*
chr10	33222550	33222841	H3K9ME2_UNC0638_peak_610	32	*D830005E20Rik*
chr10	33623977	33624369	H3K9ME2_UNC0638_peak_611	64	-
chr10	39933314	39933767	H3K9ME2_UNC0638_peak_612	50	-
chr10	41071164	41071643	H3K9ME2_UNC0638_peak_613	33	-
chr10	76961469	76962177	H3K9ME2_UNC0638_peak_614	67	*Gm35664*
chr10	77605979	77606282	H3K9ME2_UNC0638_peak_615	16	*Sumo3*
chr10	79716512	79717278	H3K9ME2_UNC0638_peak_616	47	-
chr10	79977550	79978182	H3K9ME2_UNC0638_peak_617	26	-
chr10	82484848	82485139	H3K9ME2_UNC0638_peak_618	27	*Gm1553*
chr10	97692901	97693761	H3K9ME2_UNC0638_peak_619	47	*Ccer1*
chr10	104196750	104197063	H3K9ME2_UNC0638_peak_620	57	*Gm4340*
chr10	127961477	127961813	H3K9ME2_UNC0638_peak_621	32	-
chr10	128035649	128035993	H3K9ME2_UNC0638_peak_622	22	*Naca*
chr11	4947378	4948427	H3K9ME2_UNC0638_peak_623	96	*Ap1b1*
chr11	42420027	42420328	H3K9ME2_UNC0638_peak_624	35	*Gabrb2*
chr11	43775584	43776413	H3K9ME2_UNC0638_peak_625	75	*Adra1b*
chr11	45851979	45852403	H3K9ME2_UNC0638_peak_626	17	-
chr11	49901531	49902344	H3K9ME2_UNC0638_peak_627	62	*Rasgef1c*
chr11	52764451	52765098	H3K9ME2_UNC0638_peak_628	50	*Fstl4*
chr11	54140419	54140847	H3K9ME2_UNC0638_peak_629	336	-
chr11	59012566	59012935	H3K9ME2_UNC0638_peak_630	31	*Obscn*
chr11	60932133	60932424	H3K9ME2_UNC0638_peak_631	17	*Map2k3*
chr11	67454720	67455497	H3K9ME2_UNC0638_peak_632	50	*Gas7*
chr11	67455750	67456150	H3K9ME2_UNC0638_peak_633	36	*Gas7*
chr11	68691665	68692471	H3K9ME2_UNC0638_peak_634	39	*Myh10*
chr11	83752277	83752641	H3K9ME2_UNC0638_peak_635	44	*Heatr6*
chr11	4746156	4747006	H3K9ME2_UNC0638_peak_636	43	*Gm35102*
chr11	5061532	5061950	H3K9ME2_UNC0638_peak_637	37	*Gas2l1*
chr11	6415613	6415904	H3K9ME2_UNC0638_peak_638	40	*Ppia*

© 2023. Bellver-Sanchis A. et al. Published online at http://www.aginganddisease.org/EN/10.14336/AD.2023.0424-2

chr11	11461976	11462531	H3K9ME2_UNC0638_peak_639	38	*Spata48*
chr11	14559582	14560143	H3K9ME2_UNC0638_peak_640	39	*1700046C09Rik*
chr11	16257663	16258069	H3K9ME2_UNC0638_peak_641	33	-
chr11	21992768	21993761	H3K9ME2_UNC0638_peak_642	50	*Otx1*
chr11	24952160	24952641	H3K9ME2_UNC0638_peak_643	44	*Gm39613*
chr11	31965604	31965982	H3K9ME2_UNC0638_peak_644	64	*4930524B15Rik*
chr11	35215366	35215818	H3K9ME2_UNC0638_peak_645	80	-
chr11	35797679	35798081	H3K9ME2_UNC0638_peak_646	44	*Fbll1*
chr11	38492494	38493411	H3K9ME2_UNC0638_peak_647	50	*Gm12130*
chr11	49160360	49160651	H3K9ME2_UNC0638_peak_648	44	*Olfr1394*
chr11	49169041	49169600	H3K9ME2_UNC0638_peak_649	32	*Btnl9*
chr11	49443886	49444440	H3K9ME2_UNC0638_peak_650	64	*Olfr1388*
chr11	49590049	49590565	H3K9ME2_UNC0638_peak_651	32	-
chr11	51048150	51048443	H3K9ME2_UNC0638_peak_652	37	*Olfr1375*
chr11	52098861	52099152	H3K9ME2_UNC0638_peak_653	50	*Ppp2ca*
chr11	52397922	52398284	H3K9ME2_UNC0638_peak_654	27	*9530068E07Rik*
chr11	53350576	53351140	H3K9ME2_UNC0638_peak_655	27	*Aff4*
chr11	58640496	58640831	H3K9ME2_UNC0638_peak_656	36	*Trim58*
chr11	58778537	58778915	H3K9ME2_UNC0638_peak_657	32	*Olfr315*
chr11	60699721	60700093	H3K9ME2_UNC0638_peak_658	22	-
chr11	61022027	61022607	H3K9ME2_UNC0638_peak_659	22	*Kcnj12*
chr11	69088559	69089032	H3K9ME2_UNC0638_peak_660	27	*Vamp2*
chr11	69817021	69817372	H3K9ME2_UNC0638_peak_661	28	*Spem2*
chr11	69837368	69837692	H3K9ME2_UNC0638_peak_662	32	*Tmem256*
chr11	69935798	69936351	H3K9ME2_UNC0638_peak_663	17	-
chr11	69966188	69966792	H3K9ME2_UNC0638_peak_664	24	*Cldn7*
chr11	70086665	70087113	H3K9ME2_UNC0638_peak_665	27	-
chr11	74365242	74365646	H3K9ME2_UNC0638_peak_666	32	*Olfr412*
chr11	74619986	74620385	H3K9ME2_UNC0638_peak_667	27	*Ccdc92b*
chr11	75173259	75173617	H3K9ME2_UNC0638_peak_668	46	*Mir212*
chr11	75795013	75796389	H3K9ME2_UNC0638_peak_669	112	*Gm12339*
chr11	77930932	77931624	H3K9ME2_UNC0638_peak_670	27	*Sez6*
chr11	78165048	78165400	H3K9ME2_UNC0638_peak_671	29	*Nek8*
chr11	78178381	78178672	H3K9ME2_UNC0638_peak_672	27	*Tlcd1*
chr11	84879690	84880075	H3K9ME2_UNC0638_peak_673	38	-
chr11	85885760	85886552	H3K9ME2_UNC0638_peak_674	32	-
chr11	87616718	87617946	H3K9ME2_UNC0638_peak_675	50	*Hsf5*
chr11	95824196	95824496	H3K9ME2_UNC0638_peak_676	50	-
chr11	95862043	95862534	H3K9ME2_UNC0638_peak_677	21	*B4galnt2*
chr11	96345730	96346144	H3K9ME2_UNC0638_peak_678	22	-

© 2023. Bellver-Sanchis A. et al. Published online at http://www.aginganddisease.org/EN/10.14336/AD.2023.0424-2

chr11	96365573	96365864	H3K9ME2_UNC0638_peak_679	43	*Hoxb1*
chr11	96977592	96977883	H3K9ME2_UNC0638_peak_680	32	-
chr11	97280088	97280520	H3K9ME2_UNC0638_peak_681	32	*Gm11592*
chr11	97628404	97629363	H3K9ME2_UNC0638_peak_682	52	*Epop*
chr11	99837432	99837723	H3K9ME2_UNC0638_peak_683	50	*2300003K06Rik*
chr11	99914622	99914945	H3K9ME2_UNC0638_peak_684	29	*Gm11565*
chr11	102665353	102666458	H3K9ME2_UNC0638_peak_685	110	*Meioc*
chr11	103649266	103650108	H3K9ME2_UNC0638_peak_686	31	*Rprml*
chr11	104131776	104132081	H3K9ME2_UNC0638_peak_687	46	*Crhr1*
chr11	104133029	104133352	H3K9ME2_UNC0638_peak_688	34	*Crhr1*
chr11	115053046	115053599	H3K9ME2_UNC0638_peak_689	22	*Cd300e*
chr11	115419874	115420278	H3K9ME2_UNC0638_peak_690	32	-
chr11	116918387	116919576	H3K9ME2_UNC0638_peak_691	67	*Mgat5b*
chr11	119942821	119943159	H3K9ME2_UNC0638_peak_692	34	*Baiap2*
chr11	121146182	121146726	H3K9ME2_UNC0638_peak_693	22	*Tex19.1*
chr11	121259727	121260505	H3K9ME2_UNC0638_peak_694	32	*Foxk2*
chr12	16672293	16672826	H3K9ME2_UNC0638_peak_695	22	*Greb1*
chr12	81781038	81781346	H3K9ME2_UNC0638_peak_696	44	-
chr12	20312537	20312828	H3K9ME2_UNC0638_peak_697	27	-
chr12	24831366	24831978	H3K9ME2_UNC0638_peak_698	64	*Mboat2*
chr12	36381795	36382142	H3K9ME2_UNC0638_peak_699	32	-
chr12	102128702	102129152	H3K9ME2_UNC0638_peak_700	57	-
chr12	102948928	102949219	H3K9ME2_UNC0638_peak_701	32	-
chr12	3236530	3236997	H3K9ME2_UNC0638_peak_702	36	*Rab10os*
chr12	3237149	3237543	H3K9ME2_UNC0638_peak_703	22	*Rab10os*
chr12	11881588	11882057	H3K9ME2_UNC0638_peak_704	31	*Tubb2a-ps2*
chr12	16653335	16653707	H3K9ME2_UNC0638_peak_705	27	-
chr12	30884105	30884963	H3K9ME2_UNC0638_peak_706	38	-
chr12	64472512	64472865	H3K9ME2_UNC0638_peak_707	42	*Fscb*
chr12	72890428	72890725	H3K9ME2_UNC0638_peak_708	38	-
chr12	80518354	80518664	H3K9ME2_UNC0638_peak_709	27	-
chr12	82069705	82069996	H3K9ME2_UNC0638_peak_710	32	*Gm30799*
chr12	110278250	110278748	H3K9ME2_UNC0638_peak_711	21	*Mir1247*
chr12	114513232	114513576	H3K9ME2_UNC0638_peak_712	44	*Mir7094-2*
chr13	15463686	15464501	H3K9ME2_UNC0638_peak_713	49	*Gli3*
chr13	76384010	76384333	H3K9ME2_UNC0638_peak_714	20	*Mctp1*
chr13	119488203	119489292	H3K9ME2_UNC0638_peak_715	161	*Tmem267*
chr13	18948219	18948627	H3K9ME2_UNC0638_peak_716	31	*Amph*
chr13	38345604	38346211	H3K9ME2_UNC0638_peak_717	27	*Bmp6*
chr13	46502050	46502578	H3K9ME2_UNC0638_peak_718	42	*Cap2*

© 2023. Bellver-Sanchis A. et al. Published online at http://www.aginganddisease.org/EN/10.14336/AD.2023.0424-2

chr13	55210231	55210806	H3K9ME2_UNC0638_peak_719	22	*Nsd1*
chr13	109116284	109116953	H3K9ME2_UNC0638_peak_720	44	*Pde4d*
chr13	117602481	117603005	H3K9ME2_UNC0638_peak_721	22	*Hcn1*
chr13	118714985	118715490	H3K9ME2_UNC0638_peak_722	22	*Fgf10*
chr13	3117120	3117610	H3K9ME2_UNC0638_peak_723	72	*Gm46400*
chr13	3478281	3478593	H3K9ME2_UNC0638_peak_724	37	*2810429I04Rik*
chr13	21658666	21659124	H3K9ME2_UNC0638_peak_725	19	*Olfr1361*
chr13	26769262	26769621	H3K9ME2_UNC0638_peak_726	22	*Hdgfl1*
chr13	31625759	31626050	H3K9ME2_UNC0638_peak_727	38	*Foxf2*
chr13	31626601	31626964	H3K9ME2_UNC0638_peak_728	50	*Foxf2*
chr13	47193513	47193874	H3K9ME2_UNC0638_peak_729	32	*Rnf144b*
chr13	49187547	49187838	H3K9ME2_UNC0638_peak_730	43	*Ninj1*
chr13	50657249	50657540	H3K9ME2_UNC0638_peak_731	22	*Gm32038*
chr13	52582815	52583471	H3K9ME2_UNC0638_peak_732	64	*Syk*
chr13	54372102	54372431	H3K9ME2_UNC0638_peak_733	47	*Cplx2*
chr13	54948850	54949671	H3K9ME2_UNC0638_peak_734	78	*Unc5a*
chr13	61157210	61158033	H3K9ME2_UNC0638_peak_735	32	*Ctsr*
chr13	68597463	68597812	H3K9ME2_UNC0638_peak_736	44	*1700001L19Rik*
chr13	68998916	68999327	H3K9ME2_UNC0638_peak_737	33	*Gm35161*
chr13	73846766	73847584	H3K9ME2_UNC0638_peak_738	52	*Gm30303*
chr13	74121190	74121608	H3K9ME2_UNC0638_peak_739	32	*Slc9a3*
chr13	83497186	83497547	H3K9ME2_UNC0638_peak_740	33	*Gm33274*
chr13	92425980	92426385	H3K9ME2_UNC0638_peak_741	21	*Ankrd34b*
chr13	92426707	92427208	H3K9ME2_UNC0638_peak_742	40	*Ankrd34b*
chr13	94876918	94877386	H3K9ME2_UNC0638_peak_743	50	*Otp*
chr13	98354366	98354901	H3K9ME2_UNC0638_peak_744	21	*Foxd1*
chr13	98355344	98355904	H3K9ME2_UNC0638_peak_745	22	*Foxd1*
chr13	103920565	103920899	H3K9ME2_UNC0638_peak_746	17	*D130037M23Rik*
chr13	109903018	109903503	H3K9ME2_UNC0638_peak_747	38	*Mir1904*
chr13	112993625	112994017	H3K9ME2_UNC0638_peak_748	39	*Mcidas*
chr14	40812951	40813374	H3K9ME2_UNC0638_peak_749	17	*Sh2d4b*
chr14	119137721	119138170	H3K9ME2_UNC0638_peak_750	37	*Hs6st3*
chr14	13284468	13285368	H3K9ME2_UNC0638_peak_751	41	*Synpr*
chr14	30715948	30716348	H3K9ME2_UNC0638_peak_752	27	-
chr14	56450420	56450748	H3K9ME2_UNC0638_peak_753	38	*Rnf17*
chr14	63606099	63606876	H3K9ME2_UNC0638_peak_754	74	*Xkr6*
chr14	63607207	63607498	H3K9ME2_UNC0638_peak_755	32	*Xkr6*
chr14	70485973	70486264	H3K9ME2_UNC0638_peak_756	27	-
chr14	70486566	70486935	H3K9ME2_UNC0638_peak_757	57	-
chr14	70889673	70890109	H3K9ME2_UNC0638_peak_758	21	*Gfra2*

© 2023. Bellver-Sanchis A. et al. Published online at http://www.aginganddisease.org/EN/10.14336/AD.2023.0424-2

SUPPLEMENTARY DATA

chr14	103650616	103650998	H3K9ME2_UNC0638_peak_759	52	-
chr14	104460296	104460894	H3K9ME2_UNC0638_peak_760	31	*Pou4f1*
chr14	6497521	6497812	H3K9ME2_UNC0638_peak_761	48	-
chr14	19041996	19042356	H3K9ME2_UNC0638_peak_762	34	*Gm41099*
chr14	24073130	24074084	H3K9ME2_UNC0638_peak_763	93	-
chr14	32158523	32158904	H3K9ME2_UNC0638_peak_764	38	*Ncoa4*
chr14	32377794	32378122	H3K9ME2_UNC0638_peak_765	54	*1700024G13Rik*
chr14	32597788	32598082	H3K9ME2_UNC0638_peak_766	32	-
chr14	33923228	33923652	H3K9ME2_UNC0638_peak_767	19	*Gdf10*
chr14	39471874	39472228	H3K9ME2_UNC0638_peak_768	27	*LOC432842*
chr14	39473465	39473756	H3K9ME2_UNC0638_peak_769	35	*LOC432842*
chr14	44988580	44989705	H3K9ME2_UNC0638_peak_770	40	*Ptger2*
chr14	47472790	47473081	H3K9ME2_UNC0638_peak_771	27	*Fbxo34*
chr14	50345461	50345847	H3K9ME2_UNC0638_peak_772	17	*Olfr735*
chr14	56115872	56116243	H3K9ME2_UNC0638_peak_773	44	*Gzme*
chr14	57035942	57036852	H3K9ME2_UNC0638_peak_774	58	*Gja3*
chr14	59625283	59626255	H3K9ME2_UNC0638_peak_775	55	*Shisa2*
chr14	60379036	60379627	H3K9ME2_UNC0638_peak_776	20	-
chr14	62555572	62556372	H3K9ME2_UNC0638_peak_777	51	*Fam124a*
chr14	68084061	68084720	H3K9ME2_UNC0638_peak_778	40	*Nefl*
chr14	69190590	69191050	H3K9ME2_UNC0638_peak_779	47	*Nkx3-1*
chr14	70443010	70443301	H3K9ME2_UNC0638_peak_780	27	*Mir320*
chr14	70924936	70925358	H3K9ME2_UNC0638_peak_781	33	*Gm34588*
chr14	75845315	75845606	H3K9ME2_UNC0638_peak_782	21	*Tpt1*
chr14	79213251	79213693	H3K9ME2_UNC0638_peak_783	47	*Zfp957*
chr14	102817412	102817852	H3K9ME2_UNC0638_peak_784	26	*Gm34589*
chr14	118012271	118012606	H3K9ME2_UNC0638_peak_785	22	-
chr15	92051491	92052063	H3K9ME2_UNC0638_peak_786	30	*Cntn1*
chr15	66004960	66005422	H3K9ME2_UNC0638_peak_787	25	*Kcnq3*
chr15	4375526	4375978	H3K9ME2_UNC0638_peak_788	17	*Plcxd3*
chr15	10350735	10351050	H3K9ME2_UNC0638_peak_789	19	*Gm41269*
chr15	18077696	18077987	H3K9ME2_UNC0638_peak_790	32	*4921515E04Rik*
chr15	37233901	37234636	H3K9ME2_UNC0638_peak_791	106	*Grhl2*
chr15	39197218	39199162	H3K9ME2_UNC0638_peak_792	58	*Rims2*
chr15	80287651	80288139	H3K9ME2_UNC0638_peak_793	17	*Cacna1i*
chr15	82147176	82147755	H3K9ME2_UNC0638_peak_794	26	*Srebf2*
chr15	82298627	82299377	H3K9ME2_UNC0638_peak_795	43	*Wbp2nl*
chr15	88982798	88983157	H3K9ME2_UNC0638_peak_796	50	*Mov10l1*
chr15	89498923	89499297	H3K9ME2_UNC0638_peak_797	26	*Shank3*
chr15	89499945	89500312	H3K9ME2_UNC0638_peak_798	43	*Shank3*

© 2023. Bellver-Sanchis A. et al. Published online at http://www.aginganddisease.org/EN/10.14336/AD.2023.0424-2

SUPPLEMENTARY DATA

chr15	100038594	100039116	H3K9ME2_UNC0638_peak_799	32	*Dip2b*
chr15	103271722	103272013	H3K9ME2_UNC0638_peak_800	32	-
chr15	7810022	7810515	H3K9ME2_UNC0638_peak_801	38	*Gdnf*
chr15	7810777	7811068	H3K9ME2_UNC0638_peak_802	32	*Gdnf*
chr15	11904407	11905142	H3K9ME2_UNC0638_peak_803	34	*Gm5144*
chr15	12117586	12117877	H3K9ME2_UNC0638_peak_804	27	-
chr15	25364238	25364775	H3K9ME2_UNC0638_peak_805	85	*Basp1*
chr15	29714029	29714460	H3K9ME2_UNC0638_peak_806	72	*Mir3964*
chr15	34838020	34838388	H3K9ME2_UNC0638_peak_807	28	*Kcns2*
chr15	34837387	34837771	H3K9ME2_UNC0638_peak_808	22	*Kcns2*
chr15	36315645	36315948	H3K9ME2_UNC0638_peak_809	27	*Gm41294*
chr15	39006513	39006823	H3K9ME2_UNC0638_peak_810	29	-
chr15	39076943	39077371	H3K9ME2_UNC0638_peak_811	17	-
chr15	44748726	44749017	H3K9ME2_UNC0638_peak_812	22	-
chr15	44787167	44788045	H3K9ME2_UNC0638_peak_813	31	*2310069G16Rik*
chr15	46622018	46622309	H3K9ME2_UNC0638_peak_814	45	*4930548G14Rik*
chr15	66285947	66286540	H3K9ME2_UNC0638_peak_815	33	*Gm27242*
chr15	72968234	72968835	H3K9ME2_UNC0638_peak_816	21	-
chr15	73724653	73725055	H3K9ME2_UNC0638_peak_817	17	-
chr15	75565955	75567034	H3K9ME2_UNC0638_peak_818	89	*Ly6h*
chr15	75746857	75747826	H3K9ME2_UNC0638_peak_819	32	*Mafa*
chr15	76457803	76458094	H3K9ME2_UNC0638_peak_820	32	*Scx*
chr15	78776548	78776859	H3K9ME2_UNC0638_peak_821	48	-
chr15	79109140	79109574	H3K9ME2_UNC0638_peak_822	38	-
chr15	79229325	79229672	H3K9ME2_UNC0638_peak_823	57	-
chr15	80255205	80255729	H3K9ME2_UNC0638_peak_824	102	*Atf4*
chr15	80671357	80672334	H3K9ME2_UNC0638_peak_825	66	*Fam83f*
chr15	84192958	84193539	H3K9ME2_UNC0638_peak_826	32	*Samm50*
chr15	85131347	85132220	H3K9ME2_UNC0638_peak_827	94	-
chr15	86033027	86033444	H3K9ME2_UNC0638_peak_828	27	*Gm34764*
chr15	95653967	95654771	H3K9ME2_UNC0638_peak_829	42	-
chr15	98256259	98257279	H3K9ME2_UNC0638_peak_830	60	*H1f7*
chr15	98313243	98313737	H3K9ME2_UNC0638_peak_831	22	*Olfr285*
chr15	98497268	98498250	H3K9ME2_UNC0638_peak_832	53	*Olfr279*
chr15	99294953	99295244	H3K9ME2_UNC0638_peak_833	27	-
chr15	99393227	99393588	H3K9ME2_UNC0638_peak_834	50	*Tmbim6*
chr15	99590692	99591299	H3K9ME2_UNC0638_peak_835	38	*Aqp5*
chr15	100280690	100280987	H3K9ME2_UNC0638_peak_836	36	*Tmprss12*
chr15	100615639	100615949	H3K9ME2_UNC0638_peak_837	57	*Dazap2*
chr15	100728635	100729874	H3K9ME2_UNC0638_peak_838	88	*I730030J21Rik*

© 2023. Bellver-Sanchis A. et al. Published online at http://www.aginganddisease.org/EN/10.14336/AD.2023.0424-2

chr15	102203744	102204035	H3K9ME2_UNC0638_peak_839	44	*Zfp740*
chr15	102921189	102922206	H3K9ME2_UNC0638_peak_840	117	*Hoxc13*
chr15	102954599	102955567	H3K9ME2_UNC0638_peak_841	52	*Hoxc11*
chr15	102966908	102967214	H3K9ME2_UNC0638_peak_842	22	*Hoxc10*
chr15	103502541	103503623	H3K9ME2_UNC0638_peak_843	66	*Pde1b*
chr16	36693863	36694216	H3K9ME2_UNC0638_peak_844	27	*Ildr1*
chr16	35155848	35156630	H3K9ME2_UNC0638_peak_845	35	*Adcy5*
chr16	92498347	92499061	H3K9ME2_UNC0638_peak_846	22	*Clic6*
chr16	15488452	15488806	H3K9ME2_UNC0638_peak_847	38	*Gm41424*
chr16	16818398	16818851	H3K9ME2_UNC0638_peak_848	17	*Rpl31-ps12*
chr16	18127505	18128182	H3K9ME2_UNC0638_peak_849	44	*Rtn4r*
chr16	36687247	36687765	H3K9ME2_UNC0638_peak_850	64	*Gm41451*
chr16	37916421	37916795	H3K9ME2_UNC0638_peak_851	22	*Gpr156*
chr17	69156709	69157111	H3K9ME2_UNC0638_peak_852	46	*Epb41l3*
chr17	8526791	8527082	H3K9ME2_UNC0638_peak_853	34	*Pde10a*
chr17	14279331	14279804	H3K9ME2_UNC0638_peak_854	51	*Smoc2*
chr17	26715699	26716237	H3K9ME2_UNC0638_peak_855	44	-
chr17	30901675	30902334	H3K9ME2_UNC0638_peak_856	20	*Glp1r*
chr17	42316011	42316313	H3K9ME2_UNC0638_peak_857	31	*Ptchd4*
chr17	43801656	43802336	H3K9ME2_UNC0638_peak_858	101	-
chr17	52601189	52601602	H3K9ME2_UNC0638_peak_859	28	*Kcnh8*
chr17	52601955	52602246	H3K9ME2_UNC0638_peak_860	17	*Kcnh8*
chr17	56751460	56751960	H3K9ME2_UNC0638_peak_861	48	*Nrtn*
chr17	63050512	63051009	H3K9ME2_UNC0638_peak_862	44	*Fbxl17*
chr17	68273531	68273951	H3K9ME2_UNC0638_peak_863	29	*L3mbtl4*
chr17	69439329	69439766	H3K9ME2_UNC0638_peak_864	23	*Akain1*
chr17	8341244	8342227	H3K9ME2_UNC0638_peak_865	50	*Prr18*
chr17	13206400	13206705	H3K9ME2_UNC0638_peak_866	56	*Gm10512*
chr17	24470313	24470604	H3K9ME2_UNC0638_peak_867	22	*Pgp*
chr17	27205009	27205546	H3K9ME2_UNC0638_peak_868	27	*Gm34736*
chr17	34021936	34022624	H3K9ME2_UNC0638_peak_869	39	*Ring1*
chr17	34646431	34646773	H3K9ME2_UNC0638_peak_870	22	*Atf6b*
chr17	34671150	34671465	H3K9ME2_UNC0638_peak_871	31	*Tnxb*
chr17	39847575	39848760	H3K9ME2_UNC0638_peak_872	492	*AY036118*
chr17	39843186	39846706	H3K9ME2_UNC0638_peak_873	544	*AY036118*
chr17	55445692	55446001	H3K9ME2_UNC0638_peak_874	22	-
chr17	55593744	55594162	H3K9ME2_UNC0638_peak_875	27	-
chr17	56123940	56124275	H3K9ME2_UNC0638_peak_876	37	*Sema6b*
chr17	56123190	56123498	H3K9ME2_UNC0638_peak_877	25	*Sema6b*
chr17	56776769	56777359	H3K9ME2_UNC0638_peak_878	38	*Rfx2*

© 2023. Bellver-Sanchis A. et al. Published online at http://www.aginganddisease.org/EN/10.14336/AD.2023.0424-2

SUPPLEMENTARY DATA

chr17	78595649	78596247	H3K9ME2_UNC0638_peak_879	32	-
chr17	80373159	80374055	H3K9ME2_UNC0638_peak_880	64	*Gm10190*
chr17	80944752	80945067	H3K9ME2_UNC0638_peak_881	34	*Tmem178*
chr17	84676046	84676372	H3K9ME2_UNC0638_peak_882	38	*Abcg8*
chr18	12643539	12644009	H3K9ME2_UNC0638_peak_883	36	*Ttc39c*
chr18	12973517	12973808	H3K9ME2_UNC0638_peak_884	21	*Impact*
chr18	22344392	22344739	H3K9ME2_UNC0638_peak_885	64	*Asxl3*
chr18	23310505	23310796	H3K9ME2_UNC0638_peak_886	40	*Dtna*
chr18	31634479	31634807	H3K9ME2_UNC0638_peak_887	44	-
chr18	32938908	32939374	H3K9ME2_UNC0638_peak_888	46	*Camk4*
chr18	36018006	36018594	H3K9ME2_UNC0638_peak_889	26	-
chr18	37356499	37356828	H3K9ME2_UNC0638_peak_890	14	*Pcdhb8*
chr18	65580987	65581585	H3K9ME2_UNC0638_peak_891	49	*Zfp532*
chr18	84209152	84209478	H3K9ME2_UNC0638_peak_892	19	*Zfp407*
chr18	6490169	6490697	H3K9ME2_UNC0638_peak_893	44	*Epc1*
chr18	9213223	9213514	H3K9ME2_UNC0638_peak_894	13	*Fzd8*
chr18	9213811	9215081	H3K9ME2_UNC0638_peak_895	64	*Fzd8*
chr18	19165834	19166276	H3K9ME2_UNC0638_peak_896	27	*Gm31086*
chr18	22334188	22334479	H3K9ME2_UNC0638_peak_897	32	*Gm41676*
chr18	23216677	23217110	H3K9ME2_UNC0638_peak_898	23	*Gm7788*
chr18	27810314	27810635	H3K9ME2_UNC0638_peak_899	61	*Gm33674*
chr18	33241199	33241632	H3K9ME2_UNC0638_peak_900	57	*Gm41683*
chr18	37335407	37335972	H3K9ME2_UNC0638_peak_901	49	*Pcdhb6*
chr18	39773523	39774177	H3K9ME2_UNC0638_peak_902	50	*Pabpc2*
chr18	42395236	42395809	H3K9ME2_UNC0638_peak_903	34	*Pou4f3*
chr18	50220046	50220686	H3K9ME2_UNC0638_peak_904	72	*Gm46613*
chr18	67133345	67133643	H3K9ME2_UNC0638_peak_905	27	*Gnal*
chr18	70568561	70568852	H3K9ME2_UNC0638_peak_906	22	*Mbd2*
chr18	76475901	76476335	H3K9ME2_UNC0638_peak_907	17	-
chr18	80986164	80986597	H3K9ME2_UNC0638_peak_908	23	-
chr19	8356215	8356560	H3K9ME2_UNC0638_peak_909	57	*Slc22a30*
chr19	40893997	40894844	H3K9ME2_UNC0638_peak_910	74	*Zfp518a*
chr19	47178672	47179451	H3K9ME2_UNC0638_peak_911	87	*Neurl1a*
chr19	57610626	57611386	H3K9ME2_UNC0638_peak_912	107	*Atrnl1*
chr19	3767848	3768684	H3K9ME2_UNC0638_peak_913	86	*Kmt5b*
chr19	4712351	4712668	H3K9ME2_UNC0638_peak_914	57	*Sptbn2*
chr19	5106584	5106977	H3K9ME2_UNC0638_peak_915	51	*Klc2*
chr19	8252368	8252974	H3K9ME2_UNC0638_peak_916	30	-
chr19	11912368	11913053	H3K9ME2_UNC0638_peak_917	43	*Patl1*
chr19	14398391	14398744	H3K9ME2_UNC0638_peak_918	53	-

© 2023. Bellver-Sanchis A. et al. Published online at http://www.aginganddisease.org/EN/10.14336/AD.2023.0424-2

chr19	22138798	22139266	H3K9ME2_UNC0638_peak_919	33	*Trpm3*
chr19	23687120	23687663	H3K9ME2_UNC0638_peak_920	27	*Ptar1*
chr19	25236653	25237097	H3K9ME2_UNC0638_peak_921	41	*Kank1*
chr19	28834728	28836174	H3K9ME2_UNC0638_peak_922	77	*Slc1a1*
chr19	32757709	32758165	H3K9ME2_UNC0638_peak_923	38	*Pten*
chr19	36348173	36348575	H3K9ME2_UNC0638_peak_924	50	-
chr19	36554218	36554754	H3K9ME2_UNC0638_peak_925	27	*Hectd2*
chr19	36925885	36926307	H3K9ME2_UNC0638_peak_926	32	*Btaf1*
chr19	37017628	37018539	H3K9ME2_UNC0638_peak_927	31	*Cpeb3*
chr19	41482556	41483573	H3K9ME2_UNC0638_peak_928	43	-
chr19	44147050	44147364	H3K9ME2_UNC0638_peak_929	31	*Pkd2l1*
chr19	44930808	44931498	H3K9ME2_UNC0638_peak_930	36	*Slf2*
chr19	46761499	46761790	H3K9ME2_UNC0638_peak_931	27	-
chr19	53600079	53600792	H3K9ME2_UNC0638_peak_932	72	*Smc3*
chr19	53676716	53677876	H3K9ME2_UNC0638_peak_933	122	*Rbm20*
chr19	53944890	53945560	H3K9ME2_UNC0638_peak_934	57	*Shoc2*
chr19	3388575	3389206	H3K9ME2_UNC0638_peak_935	61	*Tesmin*
chr19	3686128	3686557	H3K9ME2_UNC0638_peak_936	36	-
chr19	3987952	3988306	H3K9ME2_UNC0638_peak_937	18	*Acy3*
chr19	4756321	4756621	H3K9ME2_UNC0638_peak_938	38	*Rbm4b*
chr19	5098245	5098599	H3K9ME2_UNC0638_peak_939	69	*Rab1b*
chr19	5306615	5308113	H3K9ME2_UNC0638_peak_940	160	-
chr19	5367777	5368085	H3K9ME2_UNC0638_peak_941	28	*Eif1ad*
chr19	6194688	6194979	H3K9ME2_UNC0638_peak_942	32	*Majin*
chr19	6364228	6364520	H3K9ME2_UNC0638_peak_943	27	*Sf1*
chr19	6830696	6831050	H3K9ME2_UNC0638_peak_944	26	*Rps6ka4*
chr19	6998328	6998781	H3K9ME2_UNC0638_peak_945	51	*Fermt3*
chr19	7612320	7612999	H3K9ME2_UNC0638_peak_946	144	*Plaat5*
chr19	7671494	7671813	H3K9ME2_UNC0638_peak_947	22	*Slc22a19*
chr19	8618171	8618580	H3K9ME2_UNC0638_peak_948	24	*Slc22a6*
chr19	8757165	8757457	H3K9ME2_UNC0638_peak_949	43	*1700023D09Rik*
chr19	8793371	8793735	H3K9ME2_UNC0638_peak_950	31	*Polr2g*
chr19	8820039	8820470	H3K9ME2_UNC0638_peak_951	23	*Hnrnpul2*
chr19	8966443	8966734	H3K9ME2_UNC0638_peak_952	26	*Eef1g*
chr19	10203820	10204179	H3K9ME2_UNC0638_peak_953	32	*Tmem258*
chr19	10388746	10390069	H3K9ME2_UNC0638_peak_954	84	*Syt7*
chr19	10525537	10525995	H3K9ME2_UNC0638_peak_955	22	*Cpsf7*
chr19	10688837	10689128	H3K9ME2_UNC0638_peak_956	38	-
chr19	11770111	11770550	H3K9ME2_UNC0638_peak_957	38	*Mrpl16*
chr19	11868488	11869191	H3K9ME2_UNC0638_peak_958	24	*Olfr1419*

© 2023. Bellver-Sanchis A. et al. Published online at http://www.aginganddisease.org/EN/10.14336/AD.2023.0424-2

SUPPLEMENTARY DATA

chr19	11965898	11966232	H3K9ME2_UNC0638_peak_959	89	*Osbp*
chr19	12119261	12119626	H3K9ME2_UNC0638_peak_960	41	*Olfr76*
chr19	12119864	12120386	H3K9ME2_UNC0638_peak_961	51	*Olfr76*
chr19	12794965	12795598	H3K9ME2_UNC0638_peak_962	97	-
chr19	12904256	12904584	H3K9ME2_UNC0638_peak_963	27	*Gm5512*
chr19	13837225	13837607	H3K9ME2_UNC0638_peak_964	40	*Olfr1501*
chr19	16872811	16873172	H3K9ME2_UNC0638_peak_965	33	*Foxb2*
chr19	17394132	17394423	H3K9ME2_UNC0638_peak_966	27	*Rfk*
chr19	18671054	18671345	H3K9ME2_UNC0638_peak_967	22	-
chr19	18713097	18713454	H3K9ME2_UNC0638_peak_968	50	-
chr19	21653205	21653496	H3K9ME2_UNC0638_peak_969	32	*Abhd17b*
chr19	24042998	24043332	H3K9ME2_UNC0638_peak_970	46	*Gm41823*
chr19	24897236	24897766	H3K9ME2_UNC0638_peak_971	54	*Foxd4*
chr19	24900093	24900500	H3K9ME2_UNC0638_peak_972	27	*Foxd4*
chr19	27323175	27324058	H3K9ME2_UNC0638_peak_973	78	*Kcnv2*
chr19	29068252	29068569	H3K9ME2_UNC0638_peak_974	38	*Gm9895*
chr19	29251457	29252095	H3K9ME2_UNC0638_peak_975	36	*Jak2*
chr19	29521931	29522603	H3K9ME2_UNC0638_peak_976	49	*Ric1*
chr19	30030620	30031241	H3K9ME2_UNC0638_peak_977	29	-
chr19	31083622	31084308	H3K9ME2_UNC0638_peak_978	61	*Cstf2t*
chr19	32387883	32388294	H3K9ME2_UNC0638_peak_979	24	*2700046G09Rik*
chr19	33122868	33123386	H3K9ME2_UNC0638_peak_980	24	*Gm29946*
chr19	33497327	33497652	H3K9ME2_UNC0638_peak_981	61	*Lipo4*
chr19	36834169	36834696	H3K9ME2_UNC0638_peak_982	73	*Tnks2*
chr19	38124616	38125186	H3K9ME2_UNC0638_peak_983	57	*Gm32440*
chr19	38837069	38837383	H3K9ME2_UNC0638_peak_984	50	*Tbc1d12*
chr19	40830990	40831497	H3K9ME2_UNC0638_peak_985	106	*Ccnj*
chr19	41980751	41981042	H3K9ME2_UNC0638_peak_986	32	*Ubtd1*
chr19	42035973	42036264	H3K9ME2_UNC0638_peak_987	22	*Ankrd2*
chr19	42090506	42090797	H3K9ME2_UNC0638_peak_988	27	*Pi4k2a*
chr19	42779811	42780190	H3K9ME2_UNC0638_peak_989	35	*Gm16244*
chr19	43440220	43441651	H3K9ME2_UNC0638_peak_990	58	-
chr19	43752990	43753326	H3K9ME2_UNC0638_peak_991	44	*Cutc*
chr19	44107004	44107415	H3K9ME2_UNC0638_peak_992	31	*Cwf19l1*
chr19	45150677	45151494	H3K9ME2_UNC0638_peak_993	73	*Tlx1*
chr19	45749112	45749522	H3K9ME2_UNC0638_peak_994	27	*Npm3*
chr19	46305031	46305322	H3K9ME2_UNC0638_peak_995	20	-
chr19	46328826	46329149	H3K9ME2_UNC0638_peak_996	43	-
chr19	47014257	47015815	H3K9ME2_UNC0638_peak_997	55	*Ina*
chr19	47067856	47068405	H3K9ME2_UNC0638_peak_998	57	*Taf5*

© 2023. Bellver-Sanchis A. et al. Published online at http://www.aginganddisease.org/EN/10.14336/AD.2023.0424-2

SUPPLEMENTARY DATA

chr19	47579553	47580083	H3K9ME2_UNC0638_peak_999	44	*Slk*
chr19	47747758	47748304	H3K9ME2_UNC0638_peak_1000	93	*Cfap43*
chr19	52264349	52264978	H3K9ME2_UNC0638_peak_1001	77	*Ins1*
chr19	53903547	53903897	H3K9ME2_UNC0638_peak_1002	27	*Pdcd4*
chr19	54153349	54153698	H3K9ME2_UNC0638_peak_1003	33	*Gm41865*
chr19	56191398	56192114	H3K9ME2_UNC0638_peak_1004	60	*Gm32028*
chr19	56548514	56548954	H3K9ME2_UNC0638_peak_1005	44	*Nhlrc2*
chr19	56722191	56723345	H3K9ME2_UNC0638_peak_1006	94	*Adrb1*
chr19	56826017	56826564	H3K9ME2_UNC0638_peak_1007	61	*Tdrd1*
chr19	57361204	57361561	H3K9ME2_UNC0638_peak_1008	27	*Fhip2a*
chr19	57508051	57508853	H3K9ME2_UNC0638_peak_1009	53	*6720468P15Rik*
chr19	58511770	58512487	H3K9ME2_UNC0638_peak_1010	69	*Ccdc172*
chr19	58606035	58606545	H3K9ME2_UNC0638_peak_1011	33	*1810007D17Rik*
chr19	58729847	58730256	H3K9ME2_UNC0638_peak_1012	25	*Pnliprp1*
chr19	59171835	59172272	H3K9ME2_UNC0638_peak_1013	31	-
chr19	59219176	59220196	H3K9ME2_UNC0638_peak_1014	55	*Kcnk18*
chr19	59260722	59261313	H3K9ME2_UNC0638_peak_1015	25	*Slc18a2*
chr19	59660678	59661121	H3K9ME2_UNC0638_peak_1016	33	*Gm19956*
chr19	61216041	61216505	H3K9ME2_UNC0638_peak_1017	44	*Csf2ra*
chr19	61225150	61226700	H3K9ME2_UNC0638_peak_1018	50	*Csf2ra*
chrUn_JH584304	3801	4165	H3K9ME2_UNC0638_peak_1019	50	*LOC100861749*
chrUn_JH584304	15737	16315	H3K9ME2_UNC0638_peak_1020	154	-
chrUn_JH584304	53233	54410	H3K9ME2_UNC0638_peak_1021	308	*Pisd-ps3*
chr1_GL456211_random	73338	73629	H3K9ME2_UNC0638_peak_1022	38	*LOC102636478*
chr1_GL456211_random	112172	113228	H3K9ME2_UNC0638_peak_1023	114	*LOC100503923*
chr1_GL456211_random	174693	174984	H3K9ME2_UNC0638_peak_1024	59	*LOC100041057*
chr1_GL456212_random	128651	128973	H3K9ME2_UNC0638_peak_1025	44	-
chr1_GL456212_random	67719	68407	H3K9ME2_UNC0638_peak_1026	220	-

© 2023. Bellver-Sanchis A. et al. Published online at http://www.aginganddisease.org/EN/10.14336/AD.2023.0424-2

SUPPLEMENTARY DATA

Supplementary Table. 9 | Prediction of lysine methylation sites in GMFB using the GPS-MSP algorithm.

ID	Position	Peptide	Met-types	Score	Cutoff
AAP35430.1 glia maturation factor, beta [Homo sapiens]	17	VAEDLVE**K**LRKFRFR	K. all	3.45	0.00
AAP35430.1 glia maturation factor, beta [Homo sapiens]	20	DLVEKLR**K**FRFRKET	K. all	0.66	0.00
AAP35430.1 glia maturation factor, beta [Homo sapiens]	25	LRKFRFR**K**ETNNAAI	K. all	2.88	0.00
AAP35430.1 glia maturation factor, beta [Homo sapiens]	35	NNAAIIM**K**IDKDKRL	K. all	0.20	0.00
AAP35430.1 glia maturation factor, beta [Homo sapiens]	38	AIIMKID**K**DKRLVVL	K. all	0.42	0.00
AAP35430.1 glia maturation factor, beta [Homo sapiens]	40	IMKIDKD**K**RLVVLDE	K. all	0.51	0.00
AAP35430.1 glia maturation factor, beta [Homo sapiens]	58	GISPDEL**K**DELPERQ	K. all	1.92	0.00
AAP35430.1 glia maturation factor, beta [Homo sapiens]	74	RFIVYSY**K**YQHDDGR	K. all	0.83	0.00
AAP35430.1 glia maturation factor, beta [Homo sapiens]	97	FSSPVGC**K**PEQQMMY	K. all	0.34	0.00
AAP35430.1 glia maturation factor, beta [Homo sapiens]	108	QMMYAGS**K**NKLVQTA	K. all	1.61	0.00
AAP35430.1 glia maturation factor, beta [Homo sapiens]	110	MYAGSKN**K**LVQTAEL	K. all	0.89	0.00
AAP35430.1 glia maturation factor, beta [Homo sapiens]	119	VQTAELT**K**VFEIRNT	K. all	1.27	0.00
AAP35430.1 glia maturation factor, beta [Homo sapiens]	137	TEEWLRE**K**LGFFTNV	K. all	0.05	0.00
AAP35430.1 glia maturation factor, beta [Homo sapiens]	150	NVNFCVS**K**VFMY***	K. all	0.01	0.00

Reference

1. Dahiya, Y. *et al.* Differential regulation of innate and learned behavior by Creb1/Crh-1 in Caenorhabditis elegans. *J. Neurosci.* **39**, 7934–7946 (2019).
2. Brena, D., Bertran, J., Porta-de-la-Riva, M. *et al.* Ancestral function of Inhibitors-of-kappaB regulates *Caenorhabditis elegans* development. *Sci Rep* **10,** 16153 (2020). https://doi.org/10.1038/s41598-020-73146-5

© 2023. Bellver-Sanchis A. et al. Published online at http://www.aginganddisease.org/EN/10.14336/AD.2023.0424-2
